# The genome sequence of the commercially cultivated mushroom *Agrocybe aegerita* reveals a conserved repertoire of fruiting-related genes and a versatile suite of biopolymer-degrading enzymes

**DOI:** 10.1186/s12864-017-4430-y

**Published:** 2018-01-15

**Authors:** Deepak K. Gupta, Martin Rühl, Bagdevi Mishra, Vanessa Kleofas, Martin Hofrichter, Robert Herzog, Marek J. Pecyna, Rahul Sharma, Harald Kellner, Florian Hennicke, Marco Thines

**Affiliations:** 1 Senckenberg Biodiversity and Climate Research Centre (BiK-F), Frankfurt a. M., Germany; 2 Junior Research Group Genetics and Genomics of Fungi, Senckenberg Gesellschaft für Naturforschung, Frankfurt a. M., Germany; 3 Institute of Ecology, Evolution and Diversity, Goethe University Frankfurt, Frankfurt a. M., Germany; 4 Institute of Food Chemistry and Food Biotechnology, Justus Liebig University Giessen, Giessen, Germany; 5 LOEWE Cluster of Integrative Fungal Research (IPF), Frankfurt a. M., Germany; 6 International Institute (IHI) Zittau, Technische Universität Dresden, Zittau, Germany; 7 Project Group “Bioresources”, Fraunhofer IME, Giessen, Germany; 8 University of Applied Sciences Zittau/Görlitz, Zittau, Germany; 9 Department of Biology, Microbiology, Utrecht University, Utrecht, The Netherlands

**Keywords:** Agaricales, Basidiomycetes, Carbohydrate active enzymes, Comparative genomics, Developmental biology, Fruit body, Mushroom, White-rot

## Abstract

**Background:**

*Agrocybe aegerita* is an agaricomycete fungus with typical mushroom features, which is commercially cultivated for its culinary use. In nature, it is a saprotrophic or facultative pathogenic fungus causing a white-rot of hardwood in forests of warm and mild climate. The ease of cultivation and fructification on solidified media as well as its archetypal mushroom fruit body morphology render *A. aegerita* a well-suited model for investigating mushroom developmental biology.

**Results:**

Here, the genome of the species is reported and analysed with respect to carbohydrate active genes and genes known to play a role during fruit body formation. In terms of fruit body development, our analyses revealed a conserved repertoire of fruiting-related genes, which corresponds well to the archetypal fruit body morphology of this mushroom. For some genes involved in fruit body formation, paralogisation was observed, but not all fruit body maturation-associated genes known from other agaricomycetes seem to be conserved in the genome sequence of *A. aegerita*. In terms of lytic enzymes, our analyses suggest a versatile arsenal of biopolymer-degrading enzymes that likely account for the flexible life style of this species. Regarding the amount of genes encoding CAZymes relevant for lignin degradation, *A. aegerita* shows more similarity to white-rot fungi than to litter decomposers, including 18 genes coding for unspecific peroxygenases and three dye-decolourising peroxidase genes expanding its lignocellulolytic machinery.

**Conclusions:**

The genome resource will be useful for developing strategies towards genetic manipulation of *A. aegerita*, which will subsequently allow functional genetics approaches to elucidate fundamentals of fruiting and vegetative growth including lignocellulolysis.

**Electronic supplementary material:**

The online version of this article (10.1186/s12864-017-4430-y) contains supplementary material, which is available to authorized users.

## Background

The Black Poplar or Sword-belt Mushroom, *Agrocybe aegerita*, is a representative of the Agaricales [1], to which also the button mushroom (*Agaricus bisporus*) belongs. Causing a white-rot, *A. aegerita* is a wood-inhabiting agaric primarily degrading dead wood of deciduous trees, especially poplar and willow [2–4]. However, it has also been described as a pathogen in declining individuals of these species, as well as in some other tree species including elm, maple and oak [4]. Although *A. aegerita sensu lato* has been reported from all continents except Antarctica [4], it seems to prefer warm or mild temperate climates as it is commonly found in Southern Europe [3, 5, 6], the south-eastern United States, as well as similar climatic zones in Asia [7]. *Agrocybe aegerita sensu lato* is a commercially grown choice edible mushroom [8–10] and is reportedly used as a medicinal fungus, especially in Asia [11].

Alongside the model mushrooms *Coprinopsis cinerea* and *Schizophyllum commune*, *A. aegerita* has also been investigated to study the fundamentals of agaricomycete fruit body (mushroom) formation [5, 12–28]. Apart from the normal dikaryotic fruit body formation, *A. aegerita* is also capable of monokaryotic fruiting. This phenomenon has been analysed by classical microbiology and genetics approaches, revealing a wide spectrum of monokaryotic fruiting types, including some that complete the development until basidiospore formation [5, 12, 14, 27]. Monokaryons offer advantages for the analysis of genes involved in fruit body formation, but monokaryotic fruiter strains are also of potential interest for edible mushroom production, as they usually produce only low amounts of spores, a desired trait in mushroom farming [27, 29, 30].

To produce culinary fruit bodies, *A. aegerita* is cultivated on various lignocellulosic substrates. In most cases straw is used as a basis, which can be supplemented with sidestream products of agriculture and food production, e.g. black tea pomace, carrot mesh, citrus pellets, cocoa shells, bran powder, sawdust, cotton waste, orange peels and grape stalks [9, 10, 31–33]. Biomass production by *A. aegerita* was evaluated not only for human nutrition [10] and medical applications [34, 35], but also for recycling of abundant agro-industrial residues like olive mill wastewater [36] or poultry litter [33]. The capability of *A. aegerita* to chemically modify and degrade lignin is due to several peroxidases, H_2_O_2_-generating enzymes and laccases. So far, none of the typical lignin degrading class II peroxidases, i.e. manganese peroxidases (MnPs), lignin peroxidases, or versatile peroxidases have been found to be expressed during growth on lignocellulose or in liquid media [11]. However, *A. aegerita* produces an unspecific peroxygenase (UPO) belonging to the heme-thiolate peroxidase superfamily, which is capable of catalysing peroxidation (one-electron oxidations) and peroxygenation (oxyfunctionalisations) of a wide array of substrates [37, 38]. The actual role of this enzyme in lignocellulose disintegration is still unknown, though it has been shown to cleave recalcitrant dimeric lignin moieties [39]. Lignin-modifying laccase activity was detected both in liquid media and in lignocellulose-based solid-state cultures [33, 40]. Even though a transcriptomics study revealed some genes coding for cellulolytic enzymes [11], a comprehensive genomic analysis of *A. aegerita* and its lignocellulose-degrading enzymes is still missing.

Thus, it was the aim of the present study to sequence and annotate the genome of the dikaryotic strain *A. aegerita* AAE-3 and of a pair of mating-compatible monokaryotic sibling strains derived from it, the strains *A. aegerita* AAE-3-13 and *A. aegerita* AAE-3-32, previously characterised by Herzog et al. [27]. This strain pair represents the extremes of the monokaryotic fruiting spectrum ranging from no production of fruiting stages (‘mycelium type’) in *A. aegerita* AAE-3-13 to the production of monokaryotic low-sporulation fruit bodies (‘fruiter type’) in *A. aegerita* AAE-3-32.

## Methods

### Strains, culture conditions and isolation of nucleic acids

Strain cultivation and maintenance as well as fruit body induction of *A. aegerita* AAE-3 was done as previously described [27]. Genomic DNA of *A. aegerita* AAE-3 was extracted using *A. aegerita* AAE-3 cultures grown in liquid YMG medium (4 g yeast extract, 10 g malt extract, and 4 g glucose in 1 L of ddH_2_O) for 8 days at 24 °C in darkness at 150 rpm on an Orbitron shaker (Infors HT, Bottmingen, Switzerland). Fungal mycelium was separated from the culture medium and rinsed with sterile water. The mycelium was ground in liquid nitrogen using a mortar and pestle. DNA was isolated from the resulting powder using the method of McKinney et al. [41] with some modifications. First, the powder was distributed to several 2 mL reaction vials. For this, a tip of a spatula of the ground sample was re-suspended in 200 μL extraction buffer (50 mM Tris pH 8.0, 200 mM NaCl, 0.2 mM EDTA, 0.5% SDS, 0.1 mg/mL Proteinase K) and incubated for 30 min at 37 °C. An incubation step of 15 min followed after adding 10 μL RNAse. Afterwards, 200 μL of a phenol:chloroform:isoamyl alcohol mixture (25:24:1) was added and samples were gently mixed. Phases were separated by centrifugation and the aqueous layer was transferred to a new 2 mL reaction vial. It was then supplemented with 18 μL 3 M sodium acetate and 400 μL 99% ethanol, followed by gentle mixing. The precipitated DNA was centrifuged 60 min at 6000 *g*. The DNA pellet was washed with 70% ethanol, air-dried and then solved in RNAse free ddH_2_O. All samples were pooled and used for DNA sequencing. In the case of *A. aegerita* AAE-3-13 and *A. aegerita* AAE-3-32, a modified CTAB method [42] was applied to extract 80 μg of genomic DNA per strain. The DNA concentration was measured using a Qubit Fluorometer (Invitrogen, Carlsbad, CA, USA) using the Qubit dsDNA HS Assay Kit (Life Technologies GmbH, Darmstadt, Germany) according to the manufacturer’s instructions. RNA was isolated and then pooled from three developmental stages of *A. aegerita* AAE-3: from fruit bodies, vegetative mycelium grown on 2% malt extract agar plates, and vegetative mycelium derived from liquid YMG cultures as mentioned above. The RNeasy Plant Mini Kit (Qiagen, Hilden, Germany) was used according to the manufacturer’s protocol to isolate RNA, which was stored at −80 °C until further use. Quality analysis of genomic DNA and total RNA before sequencing, library construction, and sequencing on PacBio (RS II) and Illumina (HiSeq 2500) instruments, was carried out by the commercial sequencing provider Eurofins Genomics (Ebersberg, Germany).

### Hybrid assembly of the *A. aegerita* AAE-3 genome sequence using Illumina and PacBio data

A total of 11.37 GB of raw sequence data of *A. aegerita* AAE-3 (read length 100 nt, insert sizes 300, 800 and 3000 bp) were generated on an Illumina Hi-Seq-2500 sequencer. Vector trimmed data of all insert libraries were filtered on the basis of Phred quality scores [43]. The minimum overall read quality was set to 25 and the minimum read length was set to 90 nt, using Sickle [44]. The two monokaryotic mating-compatible sibling strains *A. aegerita* AAE-3-13 and *A. aegerita* AAE-3-32 were also sequenced using a PacBio RS II sequencing platform. PacBio reads of both the sibling strains were corrected with the help of Illumina paired end reads using Proovread [45]. Illumina-corrected reads were further self-corrected using CANU [46]. Corrected PacBio reads of strain *A. aegerita* AAE-3-13 were assembled using CANU [46] with an error rate set to 2.5%. The resulting scaffolds were merged with Illumina paired end reads using SSPACE [47]. The resulting assembly was further improved by scaffolding using PacBio reads with the SSPACE Long-read hybrid assembler [48]. This step was repeated once again on the resulting scaffolds. Subsequently, Illumina reads were used to fill gaps between the scaffolds using the SSPACE Long-read hybrid assembler. The scaffolds resulting from this were then again assembled with PacBio reads using the SSPACE Long-read hybrid assembler which slightly reduced the number of scaffolds but did not increase the genome size. Keeping this final hybrid assembly as a reference, Illumina reads of all three libraries were mapped on the reference to generate a reference-based assembly of the genome of *A. aegerita* AAE-3 using MAQ [49]. To remove ambiguities, an error correction of the reference genome was performed with the help of Illumina reads using Proovread [45]. All scaffolds having more than 90% of Ns were discarded. To estimate the completeness of the genome sequence resulting from the hybrid assembly, a CEGMA analysis [50] of the reference genome was performed.

### Reconstruction of the *A. aegerita* AAE-3-13 and *A. aegerita* AAE-3-32 monokaryon genomes

To reconstruct the genomes of *A. aegerita* AAE-3-13 and *A. aegerita* AAE-3-32, PacBio reads from both strains were mapped individually onto the reference genome of the dikaryon *A. aegerita* AAE-3 using BLASR [51] and alignments were stored in SAM file format [52]. SAM files were converted to sorted BAM files using SAMTOOLS [52]. The genome sequence of each of the monokaryons was generated by converting sorted BAM files into consensus fastq files using the BAM2CNS module of Proovread.

### Gene finding and annotation

RNASeq data from *A. aegerita* AAE-3 with a read length of 100 nt and a Phred quality score > 25 were aligned to the genome sequence using TopHat [53]. De novo transcript assembly of RNASeq data was also performed using Trinity [54]. Intron-hints were generated from the TopHat alignment using a perl script. Genemark [55–58] was used to generate a transcript-guided gene model. A spliced alignment of Trinity de novo transcripts with the genome of *A. aegerita* AAE-3 and a spliced alignment based on transcript assemblies were generated using PASA [59]. PASA transcripts were used to generate a training set using Transdecoder. This set was used to train the Augustus gene prediction tool [60]. Using the trained Augustus tool and an exon-intron hint file generated by BLAT [61], another gene model for *A. aegerita* AAE-3 was constructed. Gene models generated by Augustus, GeneMark-ET, PASA transcript assemblies, the Transdecoder training gene set, and a spliced transcript alignment generated by BLAT and GMAP [62] were used to generate a consensus gene set using the Evidence Modeler (EVM) software [63] with equal weight to all gene models.

To predict genes in the genome sequences of the monokaryotic strains *A. aegerita* AAE-3-13 and *A. aegerita* AAE-3-32, cDNA sequences of *A. aegerita* AAE-3 were mapped onto the genome sequences of the two monokaryons using GMAP with the parameters to map end-to-end cDNA. The resulting gene boundaries for each of the monokaryon genome sequence were saved in GFF file format. Protein-coding genes and protein sequences were extracted from the genome sequences and the GFF files using gffread as implemented in Cufflinks-2.2.1 [64, 65].

### Functional annotation of protein-coding genes

Amino acid sequences of all protein-coding genes from the dikaryotic strain *A. aegerita* AAE-3 were searched against the translated NCBI non-redundant nucleotide database using BLASTP [66]. Functional annotation of fruiting-related genes (FRGs) was done using InterProScan [67, 68], Blast2Go [69, 70] and the NCBI CDD database [71–75] Functional annotation of carbohydrate active enzymes (CAZymes) was carried out by using a HMMER search (Version 3.1b2; http://hmmer.org/). A database of translated sequences of all genes present in the GFF file of the dikaryotic strain *A. aegerita* AAE-3 was generated and searched for CAZymes applying the Hidden Markov Models (HMMs) for CAZymes [76]. *Agrocybe aegerita* proteins allocated to more than one CAZyme group where specified to the CAZyme group with the highest expected value (E-value). In addition, a threshold of 10^−17^ for independent E-values was set to dispose false positive hits as recommended by Yin et al. [76]. Functional annotations were added to the GFF file and unique gene IDs were assigned to each gene using in-house developed Perl scripts.

### Data access

To represent the genome and genomic features of all three strains of *A. aegerita*, a web-based genome browser was developed using JBrowse version 1.12.1 [77, 78]. Fasta sequences of genomes and GFF files for gene features for all three strains were used to visualise the genes and their distribution over the genomes of each strain. Mapping results of RNASeq reads of the dikaryotic strain *A. aegerita* AAE-3 in BAM format was also used to represent the transcriptomic support to the genes in the respective strain. All analysed data were also put in the format of a BLAST database to facilitate BLAST searches. Nucleotide sequences of all scaffolds of all three sequenced strains of *A. aegerita* have been deposited in the European Nucleotide Archive (ENA) database under the BioProject accession number PRJEB21917. Phylogeny data have been deposited in the TreeBASE repository via submission ID 22045: http://purl.org/phylo/treebase/phylows/study/TB2:S22045

## Results

### The genome sequence of *A. aegerita*

The hybrid assembly using the Illumina data of the dikaryotic strain *A. aegerita* AAE-3 and the PacBio data of one of the monokaryotic sibling strains, *A. aegerita* AAE-3-13, resulted into 127 scaffolds of 44,852,333 bp combined length. The preliminary reference-based assembly of the genome sequence of only the dikaryon *A. aegerita* AAE-3 also consisted of 127 scaffolds covering 44,852,333 bp of the genome. After removing the scaffolds with more than 90% of Ns, the final genome of *A. aegerita* AAE-3 was represented by 122 scaffolds covering 44,790,776 bp, with 3.17% of Ns. The longest scaffold was of 2,759,836 bp and the shortest scaffold was of 2417 bp. N50 and L50 of the final genome of *A. aegerita* AAE-3 was 768,404 bp and 20 respectively (Table 1).

**Table 1:** Final genome assembly statistics for *A. aegerita* AAE-3, *A. aegerita* AAE-3-13 and *A. aegerita* AAE-3-32

	*A. aegerita AAE-3*	*A. aegerita AAE-3-13*	*A. aegerita AAE-3-32*
Number of scaffolds	122	120	120
Total size of scaffolds	44,790,776	44,744,304	44,730,133
Longest scaffold	2,759,836	2,758,410	2,756,827
Shortest scaffold	2417	3316	3318
Mean scaffold size	367,138	3,722,869	372,751
Median scaffold size	233,203	240,936	240,523
N50 scaffold length	768,404	768,344	768,333
L50 scaffold count	20	20	20
scaffold %A	23.83%	24.12%	24.10%
scaffold %C	24.61%	24.82%	24.69%
scaffold %G	24.61%	24.84%	24.73%
scaffold %T	23.78%	24.03%	24.00%
scaffold %N	3.17%	2.18%	2.48%

A CEGMA analysis revealed more than 97% complete coverage of core eukaryotic genes (Additional file 1: Figure S1). The genomes of the monokaryons *A. aegerita* AAE-3-13 and *A. aegerita* AAE-3-32 were represented by 44,744,304 bp (120 scaffolds) and 44,730,133 bp (120 scaffolds), respectively (Table 1). CEGMA analyses revealed that the genome completeness in the case of *A. aegerita* AAE-3-13 and *A. aegerita* AAE-3-32 was above 97% and 94%, respectively (Additional file 1: Figure S2).

In total, 14,200 protein-coding genes were initially identified within the *A. aegerita* AAE-3 genome (for details see material and methods). Of these, 87 were incomplete and thus discarded. The final gene set for the *A. aegerita* AAE-3 genome thus consisted of 14,113 complete protein-coding genes. These were mapped onto the genome sequences of the sibling monokaryons *A. aegerita* AAE-3-13 and *A. aegerita* AAE-3-32. All 14,113 genes mapped onto *A. aegerita* AAE-3-13, and 13,611 genes could be mapped completely. In *A. aegerita* AAE-3-32, 12,951 genes could be mapped completely.

All 14,113 protein-coding genes of *A. aegerita* AAE-3 had hits to InterProScan databases, and 13,872 genes exhibited a significant match to the NCBI nt (nucleotide) database. Of the 14,113 genes, 8081 had a functional annotation in InterProScan. Referring to the GO (gene ontology) terms molecular function, biological processes and cellular components, a majority of genes were annotated to play role in metabolic processes (Additional file 2) and binding (Additional file 3) as well as to structurally contribute to general cell structure (Additional file 4). Using InterProScan, the majority of genes were annotated as coding for Cytochrome P450 proteins, dehydrogenases/reductases, and members of the major facilitator superfamily (Additional file 5).

### Fruiting-related genes (FRGs)

**Fig. 1:**
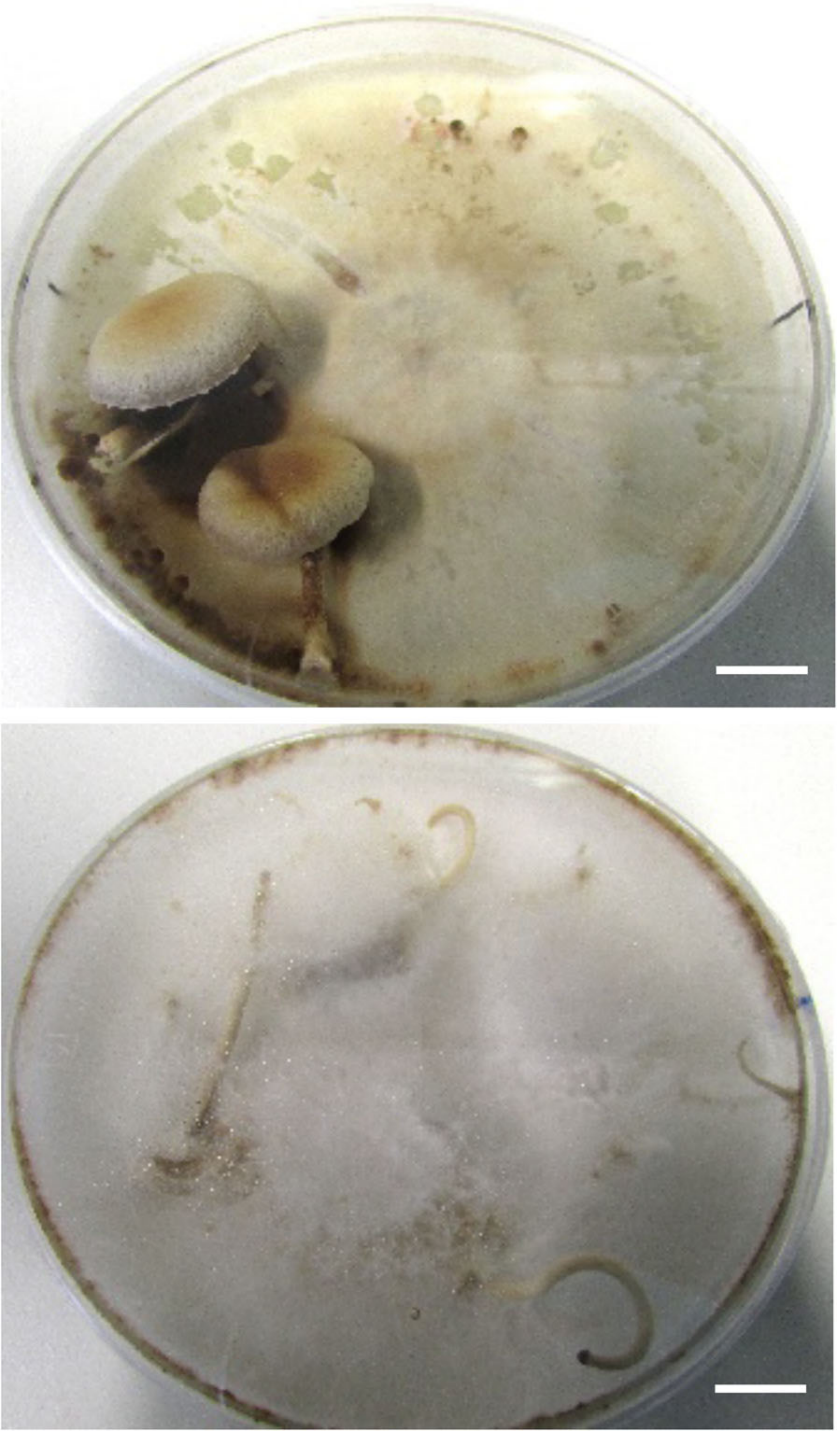
Fruit body formation of *A. aegerita* AAE-3 on 2% malt extract agar after 10 days at 25 °C in the dark followed by incubation for 13 days at 20 °C in a 12 h light/12 h darkness regime (upper picture) or in the dark (lower picture). The scale bar represents 1 cm

**Fig. 2:**
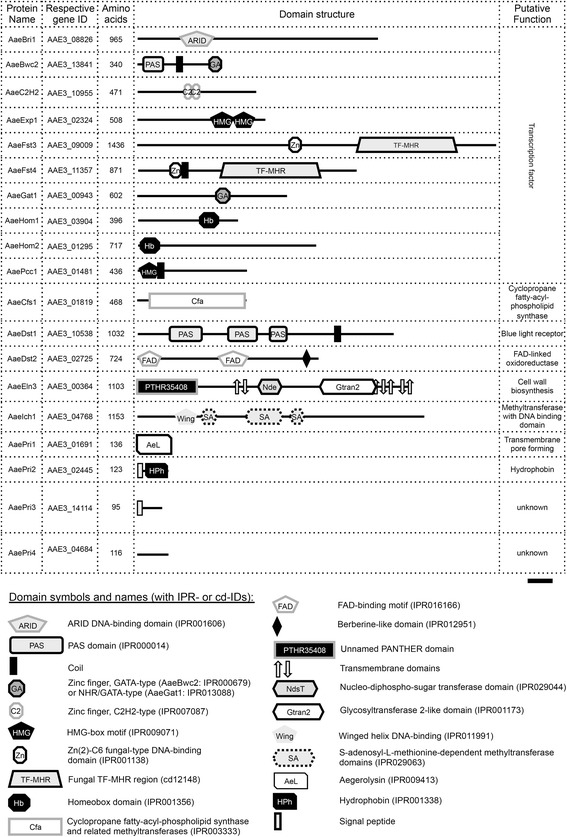
Putative homologs of agaricomycete fruiting-related genes in the genome of *A. aegerita* AAE-3 were identified by the basic local alignment search tool (BLAST; https://blast.ncbi.nlm.nih.gov/Blast.cgi) using published amino acid sequences of these genes from *A. aegerita* SM51 (=WT-1), *Coprinopsis cinerea* okayama7#130, *C. cinerea* AmutBmut pab1–1 and *Schizophyllum commune* H4–8 as query sequences (Additional file 6). Annotations of known functional elements and domains of the respective amino acid sequences were derived from InterProScan (http://www.ebi.ac.uk/interpro) and the NCBI Conserved Domain Database (CDD; https://www.ncbi.nlm.nih.gov/cdd). The scale bar represents 100 amino acids and the following abbreviations of domain names were used: ARID (AT-rich interaction domain) type DNA-binding domain; PAS (Per-Arnt-Sim) domain; HMG (high mobility group) box motif; FAD (flavin adenine dinucleotide); Fungal TF-MHR (transcription factor regulatory middle homology region)

*A. aegerita* AAE-3 is able to complete its life cycle by the formation of typical agaric fruit bodies on agar medium after three weeks, depending on light. In the absence of light, a ‘dark stipe’ phenotype occurs (Fig. 1). The genome sequence of *A. aegerita* AAE-3 was searched for putative *A. aegerita* AAE-3 homologs of genes that had previously been shown to play a role during fruit body (mushroom) formation in *A. aegerita* or other agaricomycetes (Table 2). These genes were termed fruiting-related genes (FRGs). Structural and functional annotation was done using InterProScan and NCBI CDD (Fig. 2) applying the same order of listing the FRGs both in Fig. 2 and Table 2, starting with transcription factor-encoding FRGs and ending with FRGs that have been described from *A. aegerita* SM51 previously. For each known agaricomycete FRG, the gene ID of the *A. aegerita* AAE-3 gene displaying the highest sequence homology is given. Where applicable, an InterProScan (starting with ‘IPR’ or ‘PTHR’)- or NCBI CDD-ID (starting with ‘cd’) was provided.

**Table 2:** Putative homologs of fruiting-related genes (FRGs) in *A. aegerita* AAE-3

FRG	Reference	Gene IDs (=protein IDs) of putative homolog(s) in *A. aegerita* AAE-3
*BRI1*	Ohm et al. [25]	AAE3_08826
*BWC2*	Idnurm and Heitman [107]	AAE3_13841
*C2H2*	Ohm et al. [25]	AAE3_10955, AAE3_10959, AAE3_10962
*EXP1*	Muraguchi et al. [98]	AAE3_02324
*FST3*	Ohm et al. [25]	AAE3_09009
*FST4*	Ohm et al. [25]	AAE3_11357
*GAT1*	Ohm et al. [25]	AAE3_00943
*HOM1*	Ohm et al. [25]	AAE3_03904
*HOM2*	Ohm et al. [25]	AAE3_01295
*PCC1*	Murata et al. [88]	AAE3_01481
*CFS1*	Liu et al. [89]	AAE3_01819
*DST1*	Terashima et al. [91]	AAE3_10538
*DST2*	Kamada et al. [22]	AAE3_02725
*ELN3*	Arima et al. [99]	AAE3_00364, AAE3_06792, AAE3_13318
*ICH1*	Muraguchi and Kamada [90]	AAE3_04768
*AaPRI1*	Fernandez Espinar and Labarère [93]	AAE3_01691
*AaPRI2*	Santos and Labarère [94]	AAE3_02445
*AaPRI3*	Sirand-Pugnet and Labarère [79]	AAE3_14114, AAE3_14115, AAE3_13258, AAE3_14116, AAE3_13242, AAE3_13216, AAE3_14117, AAE3_14118
*AaPRI4*	Sirand-Pugnet et al. [19]	AAE3_04684, AAE3_04675, AAE3_04665 AAE3_04667

The largest group of genes related to fruit body formation encodes the transcription factors Bri1, Bwc2, C2H2, Exp1, Fst3, Fst4, Gat1, Hom1, Hom2 and Pcc1 (Table 2), originally described from *Schizophyllum commune* and *Coprinopsis cinerea*. For all of these genes, we could identify putative *A. aegerita* AAE-3 homologs. In case of C2H2, three putative *A. aegerita* homologs were present as paralogous copies (Table 2)*.*

The second group of genes involved in fruit body formation encodes a group of functionally diverse proteins, i.e. Cfs1, Dst1, Dst2, Eln3 and Ich1 (Table 2), which have been characterised based on homology to the orthologous genes of *C. cinerea*. For Eln3, three putative *A. aegerita* paralogs were identified (Table 2). Analysing the domain structure of AaeEln3, a so far unnamed N-terminal conserved domain (PTHR35408, amino acids 1–240) was detected in addition to a Nucleotide-diphospho-sugar transferase domain (IPR029044, amino acids 475–574), and a glycosyltransferase 2-like domain (IPR001173, amino acids 712–936), framed by trans-membrane helices, both N-terminally (amino acids 382–438) and C-terminally (amino acids 914–1095) (Fig. 2).

The last group of FRGs is made up by four genes (Table 2), which were transcriptionally induced during fruit body formation of the dikaryotic *A. aegerita* wild type strain SM51 (=WT-1) [79]. According to the prediction *A. aegerita* AAE-3 has homologs for each of these FRGs (Table 2). However, in case of AaPri3 [79] and AaPri4 [19] these have undergone paralogisation into eight putative AaPri3-like and four AaPri4-like protein sequences in the *A. aegerita* AAE-3 genome. The *A. aegerita* AAE-3 homologs of AaPri3 and AaPri4 with highest similarity scores are shown in Fig. 2, encoded by AAE3_14114 and AAE3_04684, respectively. Moreover, a conserved C-terminal sequence motif (CNxxxxxxCxxGGGxCxYNxxTKRCSxxxxMRGxxxPxxCxxCxC) was observed when comparing AaePri3 to its putative homologs in *S. commune* H4–8 (gene IDs XP_003033809 and XP_003027699) and *C. cinerea* okayama7#130 (gene ID XP_001841531).

### Secreted enzymes with focus on oxidoreductases

**Fig. 3:**
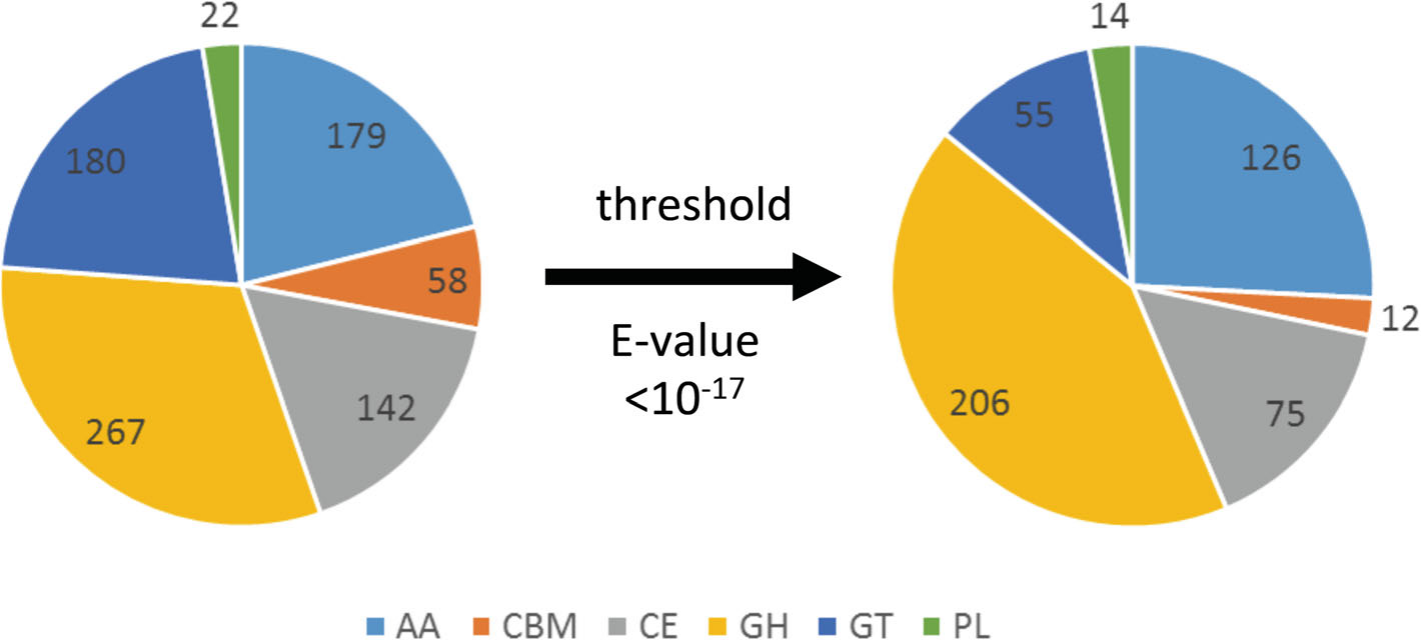
Distribution of the different CAZymes within the *A. aegerita* genome as obtained after HMMER search without (left) and with (right) an E-value threshold of 10^−17^. The following abbreviations were used: AA (auxiliary activity), CBM (carbohydrate-binding module), CE (carbohydrate esterase), GH (glycoside hydrolase), GT (glycosyl transferase) and PL (polysaccharide lyase)

A search for CAZymes using standard settings resulted in 824 genes coding for potential enzymes and carbohydrate-binding modules (CBMs) involved in degradation of polysaccharides and other plant materials (Fig. 3). When applying a more stringent threshold for the E-value (10^−17^) the total number was reduced to 476 genes including 206 putative glycoside hydrolases and 126 putative enzymes of the AA family (auxiliary activities of oxidoreductases that act in conjunction with CAZymes), and 12 CBMs. To further annotate this set in more detail, AA families were manually assigned by aligning the deduced amino acid sequence of all proteins of each subgroup to one or more reference sequences (http://www.cazy.org/Auxiliary-Activities.html), including AA1_1 and AA1_2 (Additional file 1: Figure S3), AA2 (Additional file 1: Figure S4), AA3_2, AA3_3 and AA8-AA3_1 (Additional file 1: Figure S5), AA8-AA3_1 and AA12 (Additional file 1: Figure S6), AA5_1 (Additional file 1: Figure S7), AA6 (Additional file 1: Figure S8), AA7 (Additional file 1: Figure S9) and AA9 (Additional file 1: Figure S10). This resulted in a reduction of genes coding for AA proteins from 126 to 86 (Table 3). Representatives of two other secretory peroxidase families in the *A. aegerita* genome, i.e. dye-decolourising peroxidases (DyP) and unspecific peroxygenases (UPO), were identified by BLASTP searches using reference sequences. Three DyP genes (AAE3_06734, AAE3_09015, AAE3_12346) of the subfamily D [80] are present in the genome (Additional file 1: Figure S11). Altogether 18 UPO genes were identified (Additional file 1: Figure S12), 16 of which belong to the so-called “long” UPOs (AAE3_00227 to AAE3_00237, AAE3_04643, AAE3_05814, AAE3_10946, AAE3_11945, AAE3_12863), and two to the “short” UPOs (AAE3_06358, AAE3_08521). Of the UPO-encoding genes, AAE3_00227 to AAE3_00232 and AAE3_00234 to AAE3_00237 were found to be located in close proximity on scaffold 1 in a successive orientation suggesting co-functionality.

**Table 3:** Number of genes assigned to the CAZy families by reviewing and prediction (HMM)

CAZy family AA^a^	Name	reviewed	predicted
AA1_1	laccase *sensu stricto*	14	15
AA1_2	ferroxidases	1	
AA1_3	laccase-like multi-copper oxidases	0	
AA2	class II fungal peroxidases	4	8
AA3_1	cellobiose dehydrogenase	0	28
AA3_2	aryl-alcohol oxidase/ glucose-1-oxidase	25	
AA3_3	alcohol oxidase	3	
AA3_4	pyranose oxidase	0	
AA4	vanillyl-alcohol oxidase	0	2
AA5_1	glyoxal oxidase	8	14
AA5_2	galactose oxidase	0	
AA6	1,4-benzoquinone reductase	3	3
AA7	glucooligosaccharide oxidase	2	30
AA8-AA3_1	iron reductase domain	1	1
AA9	lytic polysaccharide monooxygenase	21	21
AA12	pyrroloquinoline quinone-dependent oxidoreductase	4	4

^a^AA (auxiliary activities of redox active enzymes in conjunction with CAZymes)

Two genes (AAE3_08832 and AAE3_13544) have been grouped into the AA4 family containing vanillyl-alcohol oxidases (VAO) by HMMER search. However, they do not contain the specific FAD-binding motif present in already-characterised VAO [81, 82], and thus have been unassigned. The predicted class II peroxidases, i.e. members of the AA2 family, were subdivided after a phylogenetic analysis (Additional file 1: Figure S4) into one typical manganese peroxidase (AAE3_01449) and three atypical manganese peroxidases, missing either one (AAE3_06269, AAE3_10529) or two (AAE3_08350) glutamic acid residues involved in Mn^2+^-binding. In addition, the HMMER search assigned three intracellular ascorbate peroxidases (APx) and one cytochrome c peroxidase (CcP) to the AA2 family.

Other AA families, i.e. AA1, AA6, AA8, AA9 and AA12, were grouped to reference genes as expected.

The 15 predicted multicopper-oxidases (MCO) split into the AA1 family with 14 genes assigned to the subfamily AA1_1 (laccases *sensu stricto*) and one gene assigning to the subfamily AA1_2 (ferroxidases). Of the 14 laccase genes, two protein sequences seemed incomplete, missing either the C-terminus (AAE_12795) or N-terminus (AAE3_12295), thus not containing all expected copper-binding residues.

The gene AAE3_04795 has been assigned to the mixed cellobiose dehydrogenase (CDH) family AA3_1/AA8, due to the presence of an iron reductase domain within its sequence (Additional file 1: Figure S5 and Additional file 1: Figure S6, respectively). Nevertheless, AAE3_04795 was grouped into the AA8 family (Table 3) with an E-value of 10^−292^ in the HMMER search. Some closely related enzymes group into the AA12 family.

The four genes assigned to the pyrroloquinoline quinone-dependent (PQQ) oxidoreductases (AA12) family combining the iron reductase domain of the AA8 family with a PQQ-dependent dehydrogenase domain [83], divided into two long and two short ones. The long AA12 proteins (AAE3_04971 and AAE3_04974) showed the highest similarity to the PQQ-dependent sugar dehydrogenase (PQQ-SDH) from *C. cinerea* [83]. The shorter two genes are lacking the iron reductase domain, but do have the PQQ-binding residues. However, AAE3_05242 is lacking the histidine residue proposed to be involved in catalytic activity, present in all other *A. aegerita* AA12 proteins. Similarly to the *C. cinerea* PQQ-SDH (*Cc*SDH [83]), AAE3_04974 has a CBM1 module at its C-terminus, which is missing in AAE3_04971.

Within the AA6 family, all the three predicted 1,4-benzoquinone reductases (BQR), showed a high similarity to a characterised BQR of the brown-rot fungus *Gloeophyllum trabeum* with E-values between 10^−82^ and 10^−85^ in BLAST searches (Additional file 1: Figure S8). All 21 AA9-related sequences of *A. aegerita* showed the copper-binding residues (two histidines and one tyrosine) present in lytic polysaccharide monooxygenases (LPMOs) in accordance to Li et al. [84]. The only exception was AAE3_03716, with a lysine and an asparagine instead of the two histidine residues and, thus, suggesting a different function. Two of the predicted AA9 members (AAE3_05891 and AAE3_05893) clustered apart from the other LPMOs. They show the typical copper-binding residues, but have only three exons compared to the other LMPOs, which have five to ten exons.

## Discussion

### General genome features

The genome assembly of *A. aegerita* AAE-3 is comparable in fragmentation and continuity to other high quality mushroom genomes [23, 24, 85]. In terms of coding capacity, *A. aegerita* is similar to other well-known agaricomycetes such as *Agaricus bisporus*, *Coprinopsis cinerea*, and *Schizophyllum commune* [23, 24, 85]. The genome assembly is about 44 Mb in size comparable to the assemblies for related members of the Agaricales, which range from 35 Mb to 65 Mb [23, 24, 85–87].

### Putative *A. aegerita* AAE-3 homologs of fruiting-related genes

Ten putative homologs of known fruiting genes (FRGs) encoding transcription factors have been found. The high mobility group (HMG)-box transcription factor Pcc1 from *C. cinerea*, which has been reported to be distinct from *A* and *B* mating type genes, was shown to permit fruiting already from monokaryotic mycelium when carrying a particular recessive mutation [88]. It is not known, if a mutation in the putative *A. aegerita* homolog AaePcc1 might also be sufficient to provoke the pseudo-homokaryotic fruiting (PHF) phenotype, which has been previously reported for *A. aegerita* as a result of mating type switching [5]. In dikaryotic mycelium, the initial steps of fruiting in *S. commune* are mediated by transcription factors such as Bri1, Hom2 and Fst4, where they are crucial for the initiation of fruiting by asymmetrical colony growth and aggregation leading to fruit body initial formation [25]. An ortholog of the Cfs1 protein, described from *C. cinerea*, is also present in *A. aegerita*. In *C. cinerea*, it plays an important role in fruit body development, as it has been reported to trigger the formation of the light-induced fruit body initials from undifferentiated hyphae. In addition, Cfs1 also seemed to be acting at later stages of fruit body development in this mushroom [89]. The next step of fruit body development is the differentiation of primordia from fruit body initials. This step was reported to be influenced by C2H2 and Fst3 [25]. Interestingly, three putative homologs of *S. commune* C2H2 could be found within the *A. aegerita* AAE-3 genome including the typical adjacent C2H2 zinc-finger domains. While this implies that all three paralogs could be functional transcription factors, their function und potential redundancy needs to be evaluated in future studies.

With respect to the subsequent step of fruit body development, the development of a primordial cap and stipe, *ich1*, for which a homolog is present in *A. aegerita* as well, seems to play an important role in *C. cinerea*, as a spontaneous mutant in the progeny of a normal *C. cinerea* fruit body collected from nature exhibited fig-shaped primordia that are unable to develop further into mushrooms [90]. After the formation of the primordial cap at the apex of the primordial shaft, under proper light conditions, the primordial stipe and cap further enlarge and develop during the blue light-controlled development of fruit body primordia into young fruit bodies in *C. cinerea*. Essential to this are the genes Dst1 and Dst2, for which we could also detect putative homologs in *A. aegerita*. They are responsible for the ‘dark stipe’ or ‘etiolated stipe’ phenotype of *C. cinerea*, which occurs, e.g. when primordia are kept in darkness [22, 91, 92]. Such a ‘dark stipe’ phenotype could also be observed in the wild type dikaryon *A. aegerita* AAE-3 in case of light deprivation during early fruit body development [27]. The transcriptional induction of four additional genes during primordium development supposedly encoding different factors implicated in fruit body formation [19, 79, 93, 94], was observed in *A. aegerita*. The first of these genes, corresponding to AaePri1, has been discussed to show structural similarity to a fungal hemolysin from *Aspergillus fumigatus* [93] and is nearly identical to the sequence of the *A. aegerita* hemolysin aegerolysin [95]. Aegerolysins are a unique family of pore-forming proteins with haemolytic activity [95, 96], which have also been described from bacteria, moulds, the oyster mushroom *Pleurotus ostreatus,* and from plants. Aegerolysins themselves were reported to enhance the formation of primordia and to also stimulate the development of primordia to young fruit bodies, which suggests them to play an important role in fruiting [97]. The second of those genes, which is represented by *AaePRI2*, was characterised as a fruiting-specific hydrophobin and described to feature eight hydrophobin-characteristic cysteine residues as well as a putative signal peptide for secretion [94]. In agreement with the protein sequence features of the third primordium-induced gene characterised by Sirand-Pugnet and Labarère [79], all eight putative *A. aegerita* AAE-3 homologs of this gene (see Table 2) have a predicted N-terminal secretion signal peptide and are rich in cysteine and glycine. As there are so many potential paralogs, functional redundancy among them is conceivable, but stage-specificity or other regulatory functions cannot be ruled out at present.

In contrast to the high number of genes associated to primordium development, only a small number of genes has been described from *C. cinerea* that play a role during fruit body maturation [98, 99]. These genes were characterised from two different *C. cinerea* ‘elongationless’ mutants, the *eln2* and *eln3* mutant, which exhibit an arrest in stipe elongation [99, 100], and two *C. cinerea* ‘expansionless’ mutants carrying a mutation in the gene coding for Exp1, which triggers cap expansion [98]. However, putative *A. aegerita* homologs could only be predicted in the case of Eln3 and Exp1, suggesting a lower degree of conservation of fruit body maturation-associated genes.

Summarising the repertoire of FRGs in *A. aegerita* AAE-3 in comparison with other model mushrooms, two aspects might be promising to pursue in future studies. Among the FRGs of *A. aegerita* AAE-3, *AaePRI1* might be a most interesting candidate FRG for prospective characterisation by functional genetics approaches. The putative aegerolysin protein encoded by Aae*PRI1* might be an important regulator of agaricomycete fruiting productivity since the aegerolysin protein family member ostreolysin from *P. ostreatus* has been shown to enhance fruit body development [97]. Furthermore, a paralogisation was observed for a number of *A. aegerita* AAE-3 FRGs, i.e. *AaeELN3*, *AaeC2H2*, *AaePRI3* and *AaePRI4*, which suggests a more complex genetic regulation of fruit body development in *A. aegerita* in comparison to other model mushrooms.

### Enzymes of the AA family

Apart from exhibiting an archetypal mushroom phenotype and thus providing a potential model for fruit body development, *A. aegerita* is well equipped with enzymes for degrading biopolymers and thus may also provide insights into the decomposition of wood and other plant substrates. *Agrocybe aegerita* is usually cultured on lignocellulose-containing substrates. It was, however, reported to be an unspecific white-rot fungus according to its rotting patterns [40]. Comparing the amount of genes coding for CAZymes relevant for lignin degradation reveals more similarity of *A. aegerita* to white-rot fungi than to litter decomposers, such as *C. cinerea* and *A. bisporus* possessing only one and two genes of the AA2 family, respectively, as well as less UPO genes (Additional file 1: Figure S13). The ligninolytic ability of *A. aegerita* was documented for wheat straw and beech wood as well as for side streams of the food industry, but was lower as compared to specialised white-rot fungi [31, 40]. When cultivated on beech wood, *A. aegerita* showed similar laccase activities as typical white-rot fungi, but was lacking MnP activity [40]. However, genes coding for this peroxidase were found to be present in its genome in the present study, suggesting regulated expression. This may be explained by compensation through the production of unspecific peroxygenases (UPOs) that were described for the first time in *A. aegerita* [37, 101] and might be the basis for the high versatility of the fungus in terms of the oxyfunctionalisation reactions. This is in accordance with a transcriptomic approach [11], in which only low expression levels of MnP genes on complex media were found, but high levels of long UPO transcripts. It is noteworthy that Isikhuemhen et al. [33] detected peroxidase activity when growing *A. aegerita* on a wheat-straw-based substrate, even if laccase activity exceeded that of peroxidase up to ten times in this setting. The relatively high laccase activity of *A. aegerita* is comparable to other white-rot fungi and may reflect the number of MCO genes found in its genome. Most white-rot fungi have five to 15 MCO genes [102], most of which belong to the CAZy family AA1_1, also known as the *sensu*-*stricto*-laccases [103]. *Agrocybe aegerita* has 13 genes coding for *sensu*-*stricto-*laccases of subfamily 1 and one gene coding for a putative laccase of the subfamily 2. Interestingly, the latter gene (AAE3_12281) is more similar to a laccase of the litter decomposer *Coprinopsis cinerea* and not with the respective laccase of the white-rotter *Pleurotus ostreatus* LACC2 (also known as POXA3).

Other enzymes of *A. aegerita* belonging to the AA family were reported to oxidise aromatic alcohols, such as veratryl alcohol and benzyl alcohol, with molecular oxygen as co-substrate while delivering H_2_O_2_ [37, 40]. The AA3 group is the largest group amongst the H_2_O_2_-generating enzymes in *A. aegerita*. H_2_O_2_ is needed for the functioning of UPOs, DyPs and class II peroxidases of the AA2 family, all present in the *A. aegerita* genome. In the proteomic approach of Wang et al. [11], peptides fitting to *A. aegerita* AAE-3 DyP-type peroxidase AAE3_12346 were detected. Furthermore, in that study, sequences fitting to H_2_O_2_-producing glyoxal oxidase AAE3_06793 of this study were found. In comparison to other basidiomycete genomes, the number of genes grouping into the AA3_2 subfamily seems to be rather high [85, 104] (Additional file 1: Figure S13). Overall, genes of the AA3_2 subfamily seem to be more pronounced in white-rot than in brown-rot fungi, which is consistent with the fact that brown-rot fungi do not have genes of the AA2 family. In addition, the *A. aegerita* genome contains four and eleven genes coding for putative GH6 and GH7 enzymes, respectively, also suggesting a white-rot decay [102]. Of these putative GH enzymes, three GH7 (AAE3_12287, AAE3_05957, AAE3_05953) and one GH6 (AAE3_13829) as well as 26 other putative CAZymes, amongst others the LMPOs (see Additional file 1: Figure S10) harbour a C-terminal CBM1 domain underpinning the lignocellulolytic ability of *A. aegerita*. Although *A. aegerita* is known as a white-rot fungus, its enzyme equipment differs from that of prototypical white-rot fungi, in line with the substrate versatility of this fungus. However, litter-decomposing fungi, such as *A. bisporus* and *C. cinerea*, which are also able to degrade lignin, show some similarities to *A. aegerita* in terms of their enzyme inventory (Additional file 1: Figure S13). For instance, the genome of *A. aegerita* contains 21 genes coding for putative hydrolases acting on β-linked polysaccharides grouped into the CAZy glycoside hydrolase family GH5 (Additional file 1: Figure S14) similar to the button mushroom, *A. bisporus*, with 19 GH5 genes and more than twice the number of GH5 genes normally found in wood-colonising white-rot fungi [85]. A similar pattern can be observed with respect to UPO genes, of which *A. bisporus* carries 24, while *A. aegerita* carries 18. This is far more than observed for classical white-rot basidiomycetes sequenced so far, with the exception of *Auricularia subglabra*, which features 16 UPO genes.

However, the comparisons with respect to CAZymes is based on a comparison of basidiomycete genomes and, thus, needs to be treated with caution, as only few of these enzymes have been studied in detail in terms of their function. As a consequence, predictions concerning degradation characteristics are fraught with uncertainty. Further studies monitoring the expression profiles of the lignocellulolytic CAZy genes [105, 106] in *A. aegerita* cultures and measurements of enzymatic activity need to be conducted and assessed in order to elucidate the function of enzymes involved in the degradation of recalcitrant substrates.

## Conclusion

In summary, the archetypal mushroom morphology, the versatile biopolymer degradation potential, and the culinary quality of *A. aegerita* render this fungus an interesting model organism for studying fruit body development, biodegradation and aroma production. The annotated genome sequence provided in this study is a first step in this direction, which needs to be followed up by further studies investigating the possibility of genetic manipulation and, potential application of the results from this for industrial biotechnology, directed breeding, and commercial mushroom farming. Once genetic manipulation of *A. aegerita* is achieved, its white-rot potential, the use of its CAZy family enzymes for biotechnology (e.g. for biopolymer degradation), and its archetypal mushroom formation can be investigated in detail.

## Additional files

Additional file 1: Supplementary Figures. (PDF 1068 kb)

Additional file 2: Table S1. *Agrocybe aegerita* AAE-3 genes in subcategories of GO term “molecular function”. (DOCX 31 kb)

Additional file 3: Table S2. *Agrocybe aegerita* AAE-3 genes in subcategories of GO term “biological processes”. (DOCX 31 kb)

Additional file 4: Table S3. *Agrocybe aegerita* AAE-3 genes in subcategories of GO term “cellular components”. (DOCX 31 kb)

Additional file 5: Table S4. Annotation of *Agrocybe aegerita* AAE-3 genes using InterProScan. (DOCX 254 kb)

Additional file 6: Table S5. Proteins encoded by fruiting-related genes from different agaricomycetes species. (DOCX 34 kb)

## Abbreviations

AA: Auxiliary activity
ARID: AT-rich interaction domain
bp: basepair(s)
BQR: 1,4-benzoquinone reductase
CAZyme: Carbohydrate active enzyme
CBM: Carbohydrate-binding module
CDD: Conserved Domain Database
CDH: Cellobiose dehydrogenase
CE: Carbohydrate esterase
CEGMA: Core eukaryotic gene mapping approach
DyP: Dye-decolorising peroxidase
FAD: Flavin adenine dinucleotide
FRG: Fruiting-related gene
GH: Glycoside hydrolase
GO: Gene ontology
GT: Glycosyl transferase
HMG: High mobility group
HMM: Hidden Markov Model
LMPO: Lytic polysaccharide monooxygenase
Mb: Megabase(s)
MCO: Multicopper oxidase
MnP: Manganese peroxidase
NCBI: National Center for Biotechnology Information
nt: Nucleotide(s)
PAS: Per-Arnt-Sim domain
PL: Polysaccharide lyase
PQQ: Pyrroloquinoline quinone-dependent
TF-MHR: Transcription factor regulatory middle homology region
UPO: Unspecific peroxygenase
VAO: Vanillyl-alcohol oxidase
