# Supplementary material for: The genome sequence of the commercially cultivated mushroom Agrocybe aegerita reveals a conserved repertoire of fruiting-related genes and a versatile suite of biopolymer-degrading enzymes
Source: BMC Genomics. 2018 Jan 15;19:48. doi: 10.1186/s12864-017-4430-y (PMC5769442; doi:10.1186/s12864-017-4430-y)
Supplement: Supplementary file 1 — Supplementary Figures. (PDF 1068 kb) [file 12864_2017_4430_MOESM1_ESM.pdf]

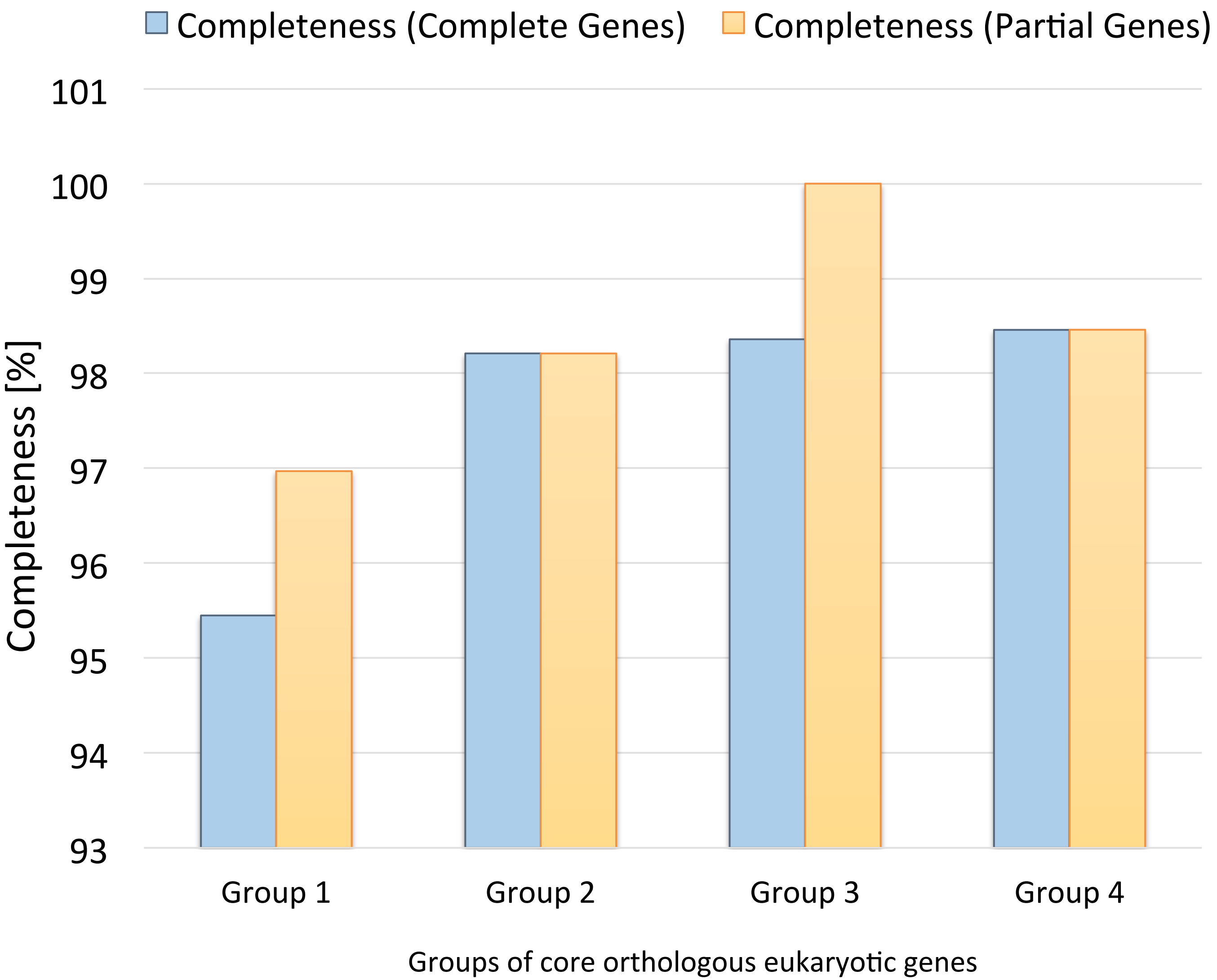

Figure S1 Genome completeness assessment of the *Agrocybe aegerita* AAE-3 genome via a core eukaryotic gene mapping approach (CEGMA). Group 1 is the least while Group 4 is the most conserved group of core orthologous eukaryotic genes

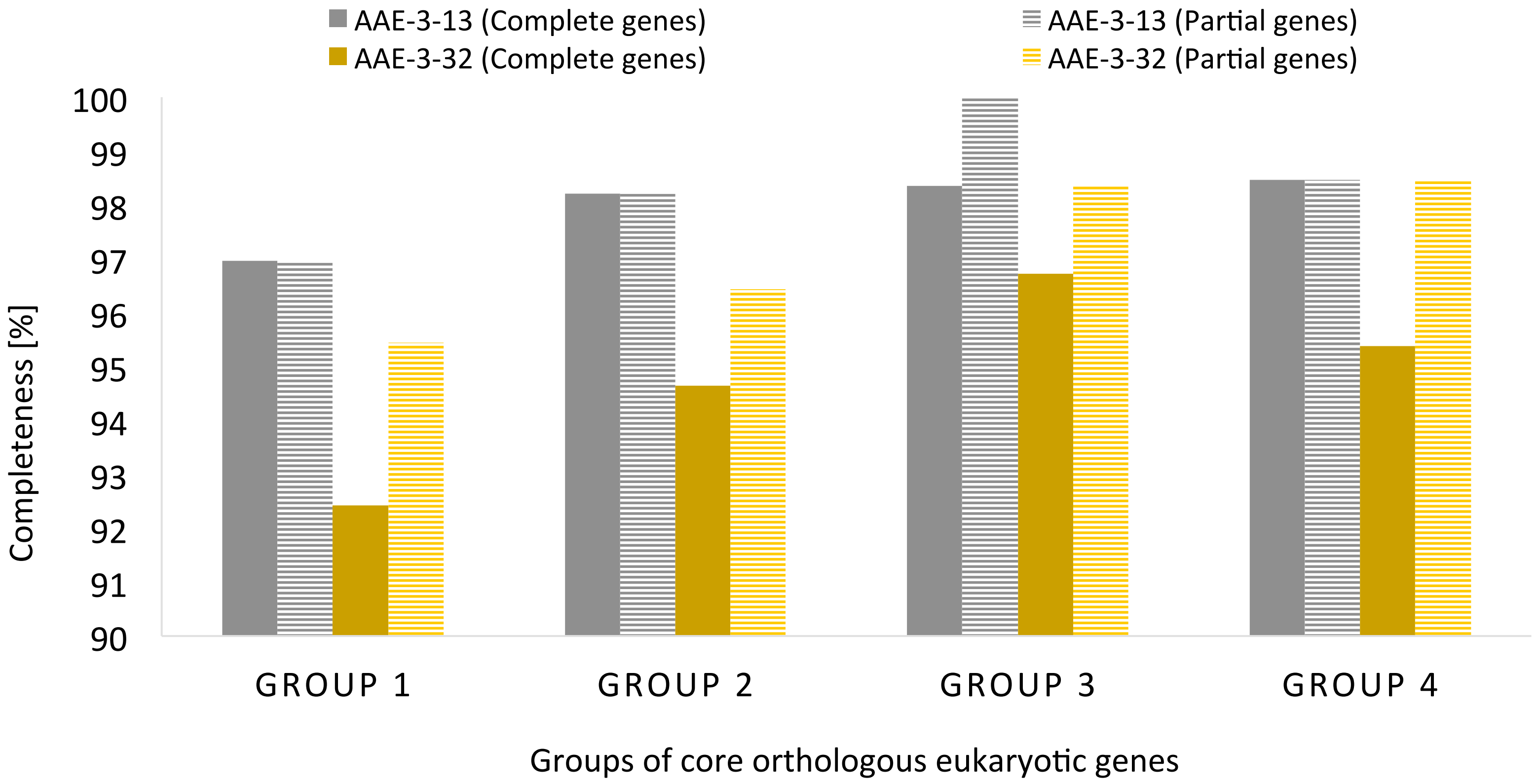

Figure S2 Genome completeness assessment of the *Agrocybe aegerita* AAE-3-13 and the *A. aegerita* AAE-3-32 genome via a core eukaryotic gene mapping approach (CEGMA). Group 1 is the least while Group 4 is the most conserved group of core orthologous eukaryotic genes

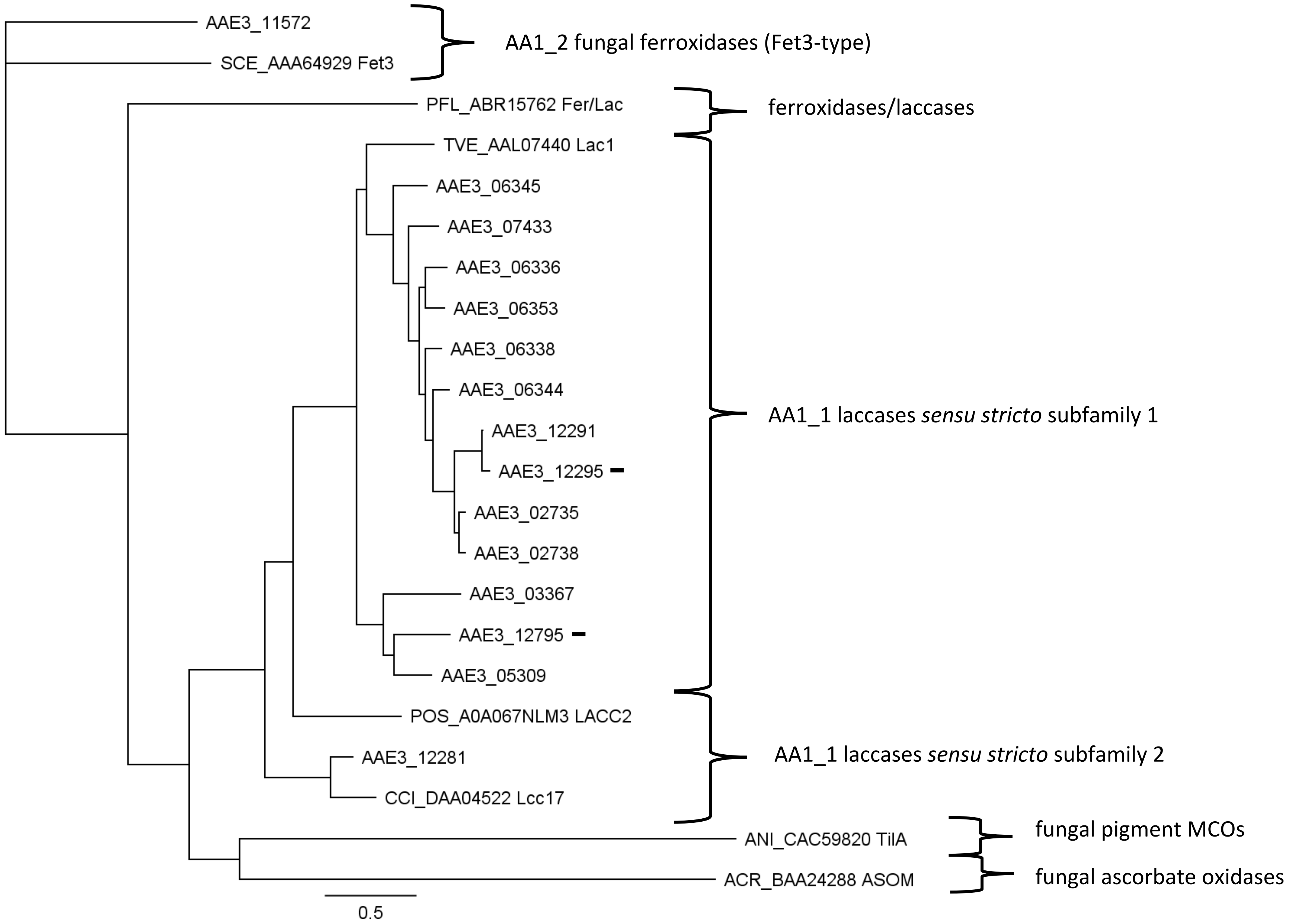

Figure S3 Phylogenetic tree (PhyML) of aligned multicopper oxidases (MCOs) deduced protein sequences of *Agrocybe aegerita* AAE-3 (AAE3) and reference protein sequences from *Sacharomyces cerevisiae* (SCE), *Phanerochaete flavidoalba* (PFL), *Trametes versicolor* (TVE), *Pleurotus ostreatus* (POS), *Coprinopsis cinerea* (CCI), *Aspergillus nidulans* (ANI) and *Acremonium* sp. (ACR). Protein codes from reference sequences refer to NCBI GenBank (http://www.ncbi.nlm.nih.gov/) or UniProtKB (http://www.uniprot.org). A minus marks partial MCO sequences

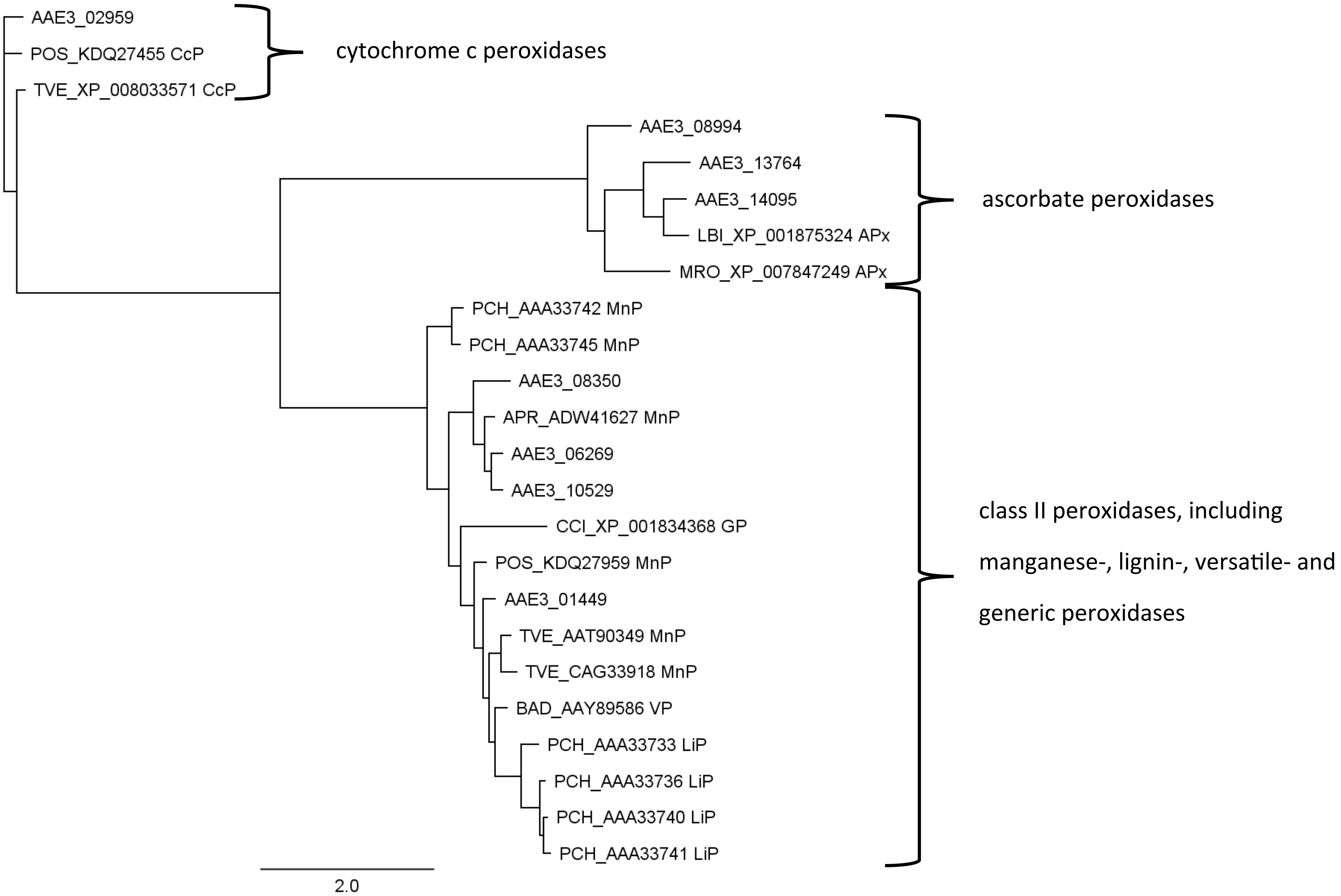

Figure S4 Phylogenetic tree (PhyML) of aligned deduced protein sequences of the *Agrocybe aegerita* AAE-3 (AAE3) CAZy family AA2 class II fungal peroxidases (includes also class I peroxidases cytochrome c peroxidase, CcP and intracellular ascorbate peroxidases, APx) and reference protein sequences from *Pleurotus ostreatus* (POS), *Trametes versicolor* (TVE), *Laccaria bicolor* (LBI), *Moniliophthora roreri* (MRO), *Phanerochaete chrysosporium* (PCH), *Agrocybe praecox* (APR), *Coprinopsis cinerea* (CCI) and *Bjerkandera adusta* (BAD). Protein codes from reference sequences refer to NCBI GenBank (http://www.ncbi.nlm.nih.gov/). Enzyme abbreviations: cytochrome c peroxidase (CcP), ascorbate peroxidase (APx), generic peroxidase (GP), manganese peroxidase (MnP), lignin peroxidase (LiP), versatile peroxidase (VP)

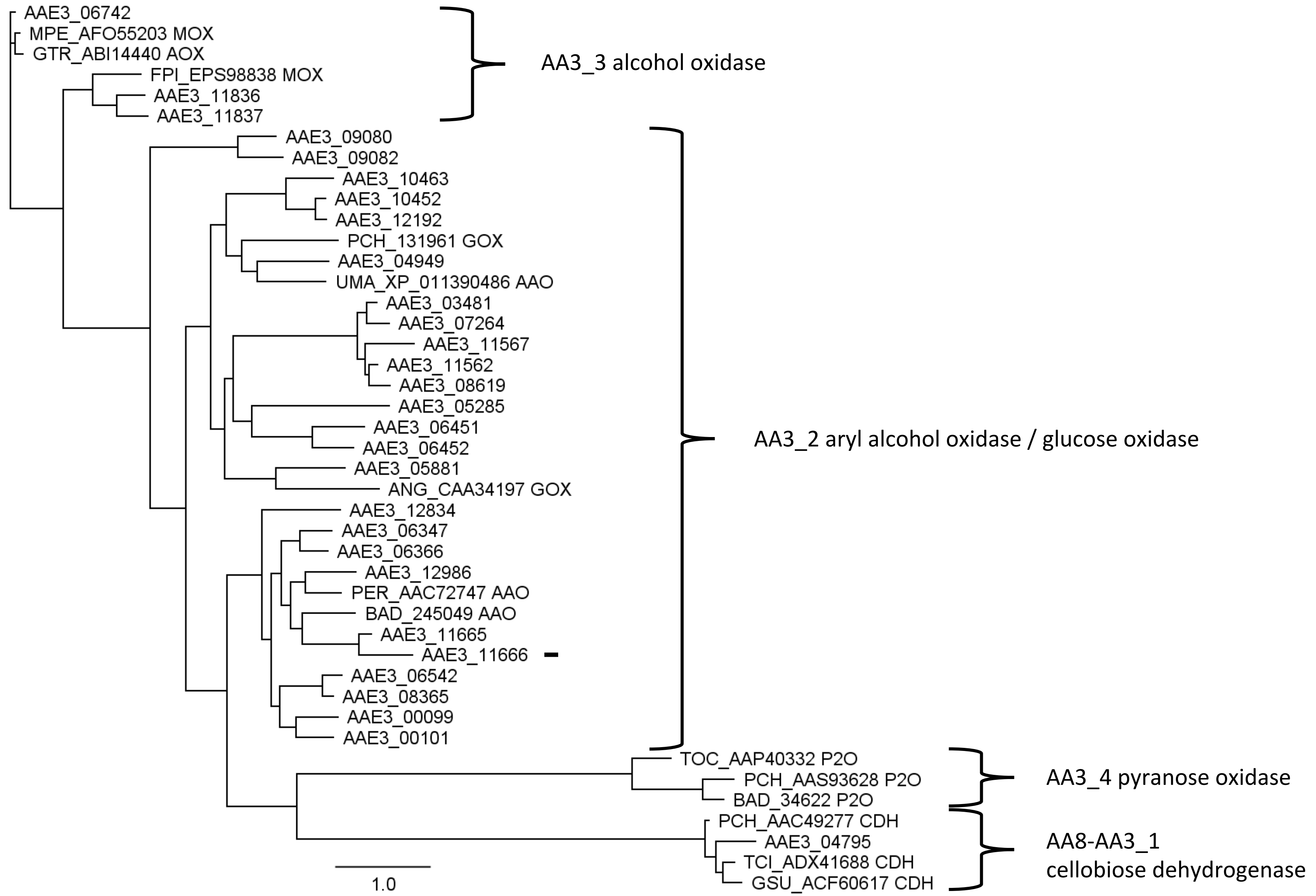

Figure S5 Phylogenetic tree (PhyML) of aligned deduced GMC (glucose, methanol, choline) oxidoreductase protein sequences from *Agrocybe aegerita* AAE-3 (AAE3) and reference protein sequences from *Moniliophthora perniciosa* (MPE), *Gloeophyllulm trabeum* (GTR), *Fomitopsis pinicola* (FPI), *Phanerochaete chrysosporium* (PCH), *Ustilago maydis* (UMA), *Aspergillus niger* (ANG), *Pleurotus eryngii* (PER), *Bjerkandera adusta* (BAD), *Trametes ochracea* (TOC), *Trametes cinnabarina* (TCI) and *Gelatoporia subvermispora* (GSU). Protein codes from reference sequences refer to NCBI GenBank (http://www.ncbi.nlm.nih.gov/) or JGI Protein IDs (http://genome.jgi.doe.gov/programs/fungi/index.jsf). Enzyme abbreviations: alcohol oxidase (AOX), methanol oxidase (MOX), aryl-alcohol oxidase (AAO), glucose oxidase (GOX), cellobiose dehydrogenase (CDH), pyranose 2-oxidase (P2O). A minus marks a partial GMC oxidoreductase sequence

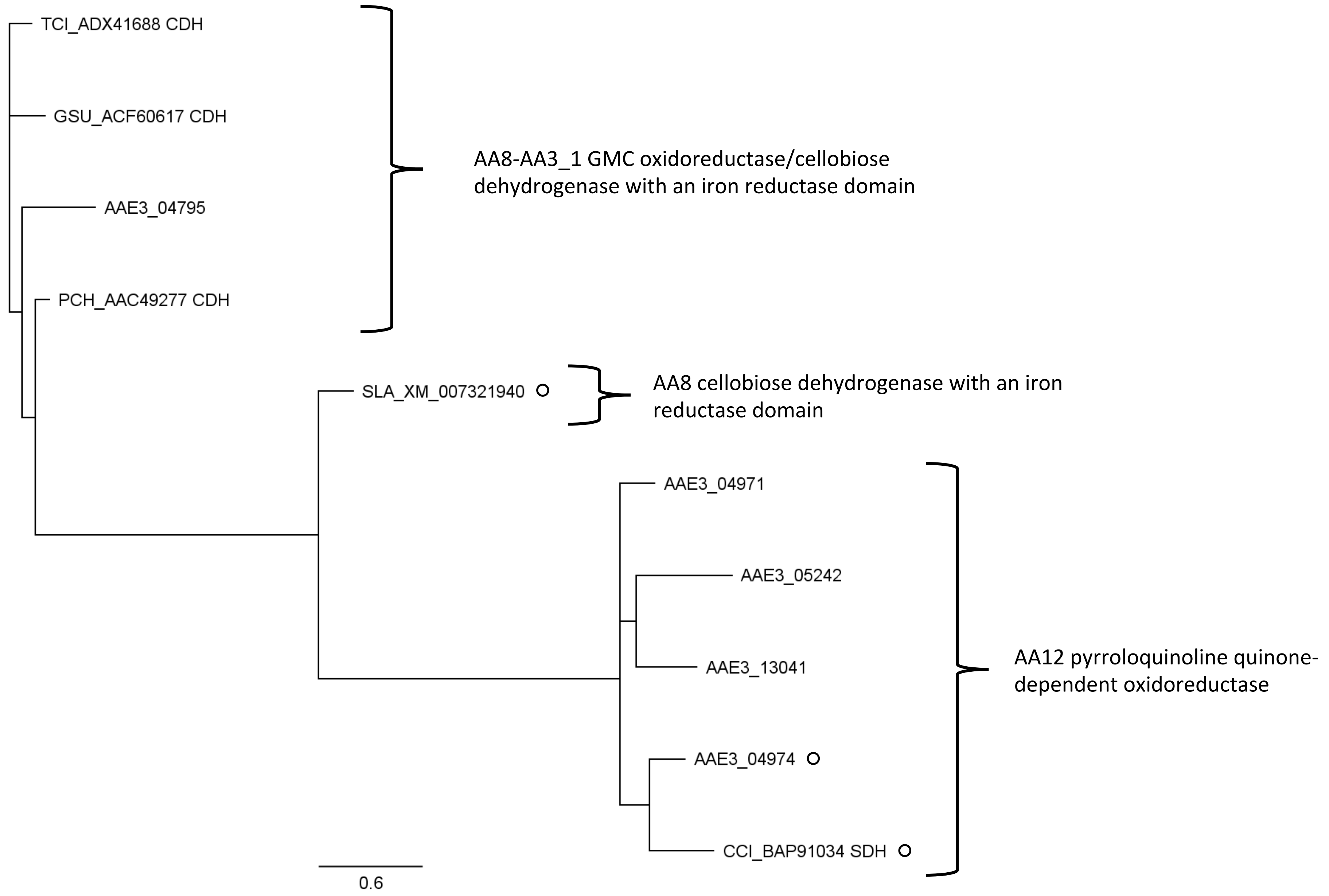

Figure S6 Phylogenetic tree (PhyML) of aligned deduced protein sequences of *Agrocybe aegerita* AAE-3 (AAE3) and reference protein sequences from *Trametes cinnabarina* (TCI), *Gelatoporia subvermispora* (GSU), *Phanerochaete chrysosporium* (PCH), *Serpula lacrymans* (SLA) and *Coprinopsis cinerea* (CCI). Protein codes from reference sequences refer to NCBI GenBank (http://www.ncbi.nlm.nih.gov/). Enzyme abbreviations: cellobiose dehydrogenase (CDH), sugar dehydrogenase (SDH). A circle marks protein sequences with a C-terminal CBM1 (carbohydrate binding module 1) domain

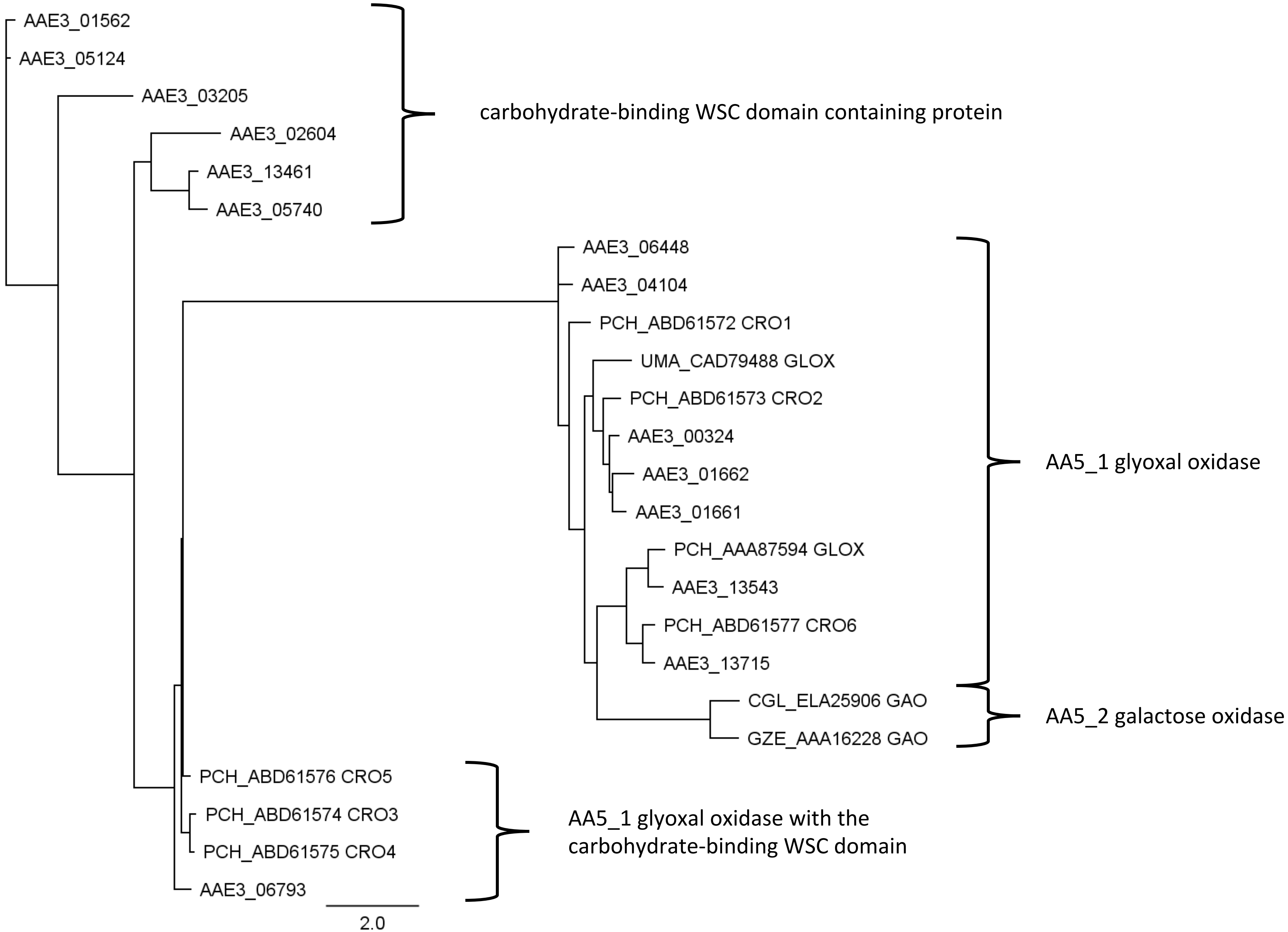

Figure S7 Phylogenetic tree (PhyML) of aligned deduced protein sequences of *Agrocybe aegerita* AAE-3 (AAE3) and reference protein sequences from *Phaneroachaete chrysosporium* (PCH), *Ustilago maydis* (UMA), *Colletotrichum gloeosporioides* (CGL) and *Gibberella zeae* (GZE). Protein codes from reference sequences refer to NCBI GenBank (http://www.ncbi.nlm.nih.gov/) or UniProtKB (http://www.uniprot.org). Enzyme abbreviations: copper radical oxidase (CRO), glyoxal oxidase (GLOX), galactose oxidase (GAO)

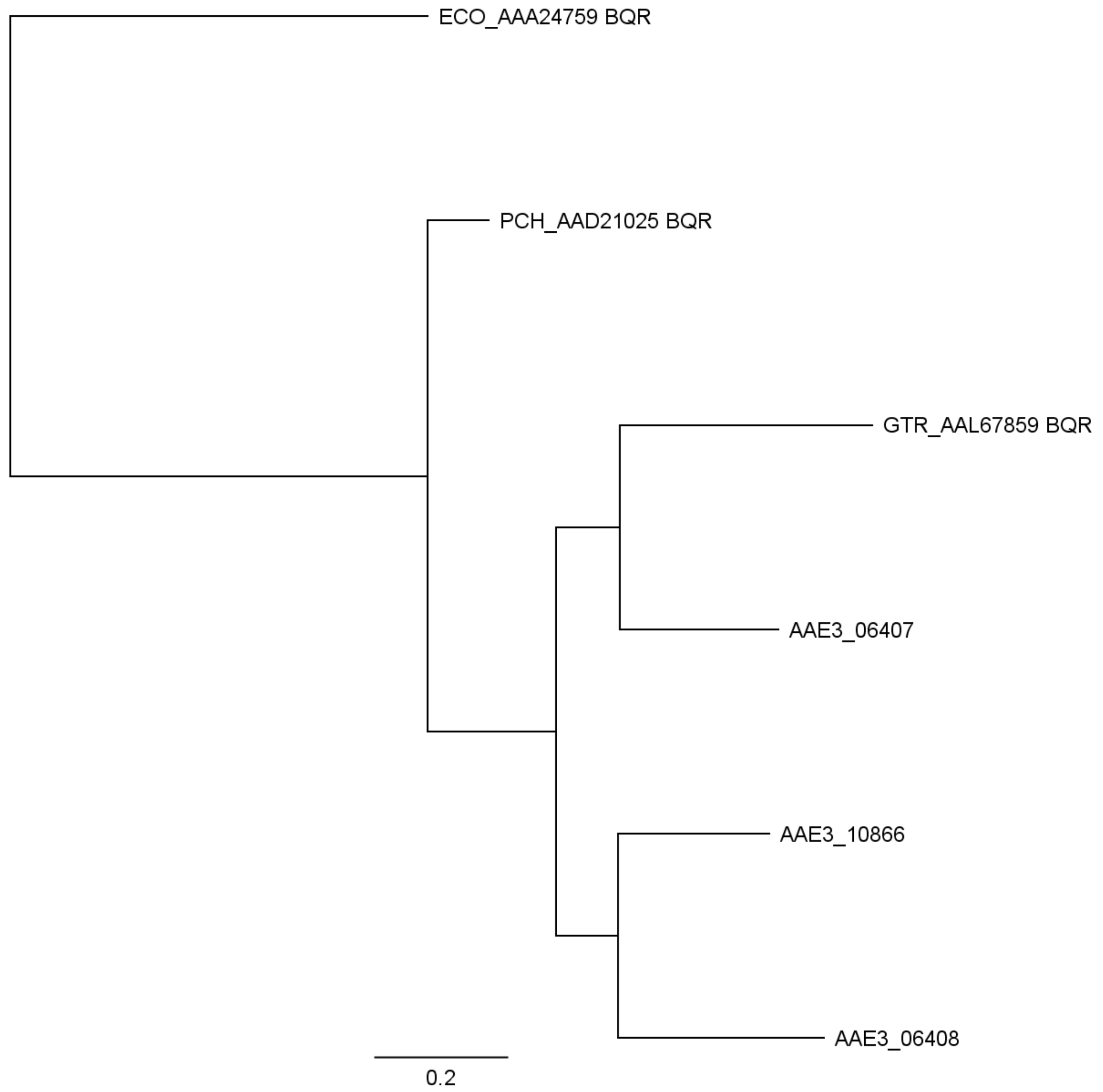

Figure S8 Phylogenetic tree (PhyML) of aligned deduced protein sequences of 1,4-benzoquinone reductases (BQR, CAZy family AA6) from *Agrocybe aegerita* AAE-3 (AAE3) and reference protein sequences from *Escherichia coli* (ECO), *Phaneroachaete chrysosporium* (PCH) and *Gloeophyllum trabeum* (GTR). Protein codes from reference sequences refer to NCBI GenBank (http://www.ncbi.nlm.nih.gov/)

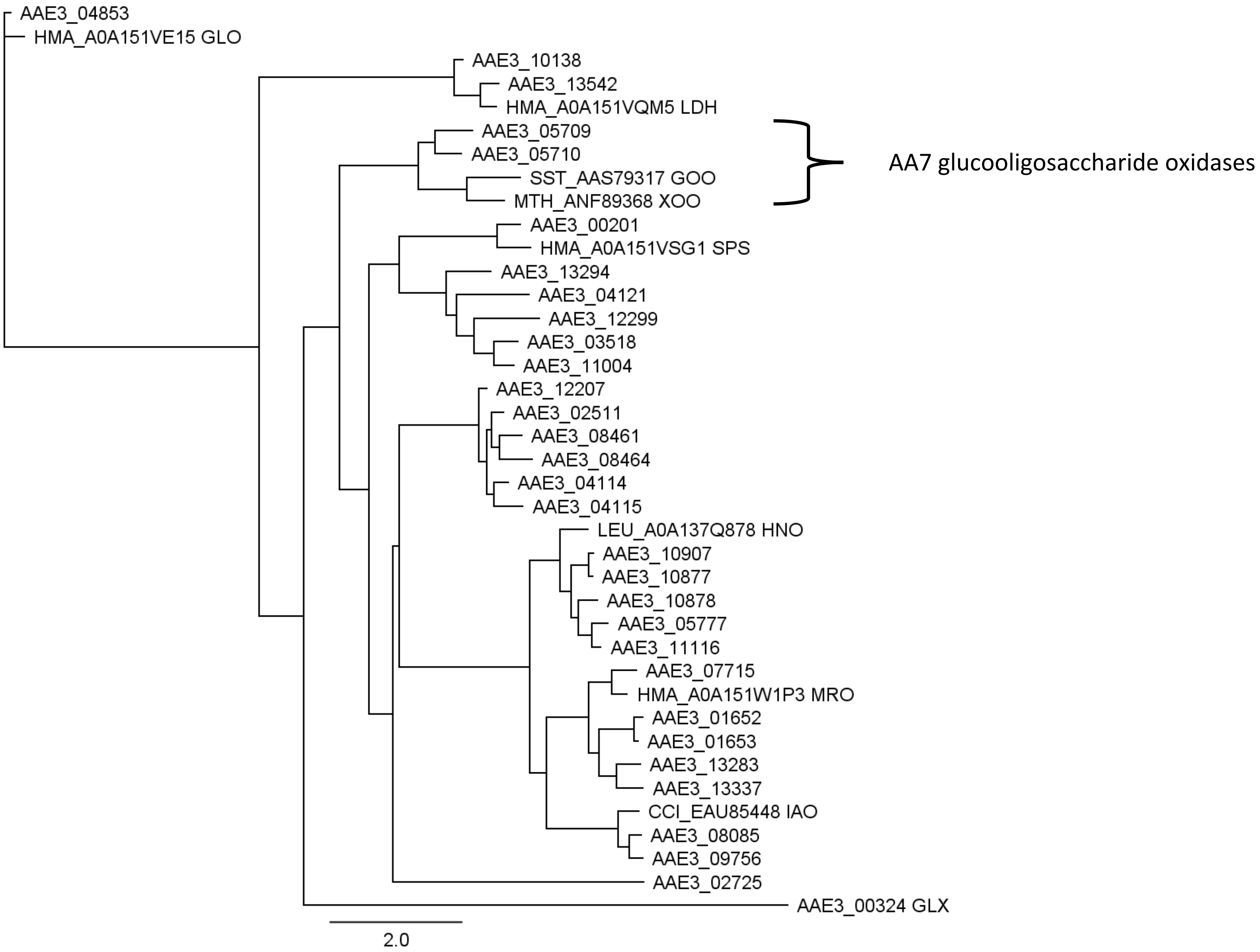

Figure S9 Phylogenetic tree (PhyML) of aligned deduced protein sequences of *Agrocybe aegerita* AAE-3 (AAE3) and reference protein sequences from *Hypsizigus marmoreus* (HMA), *Sarocladium strictum* (SST), *Myceliophthora thermophila* (MTH), *Leucoagaricus* sp. (LEU), *Coprinopsis cinerea* (CCI) and the deduced protein sequence of a *A. aegerita* glyoxal oxidase (AAE3_00324 GLX) as an outgroup. Protein codes from reference sequences refer to NCBI GenBank (http://www.ncbi.nlm.nih.gov/) or UniProtKB/Swiss-Prot (http://http://www.uniprot.org/). Enzyme abbreviations: L-gulonolactone oxidase (GLO), D-lactate dehydrogenase (LDH), glucooligosaccharide oxidase (GOO), xylooligosaccharide oxidase (XOO), solanapyrone oxidase (SPS), 6-hydroxy-D-nicotine oxidase, mitomycin radical oxidase (MRO) and isoamyl alcohol oxidase (IAO)

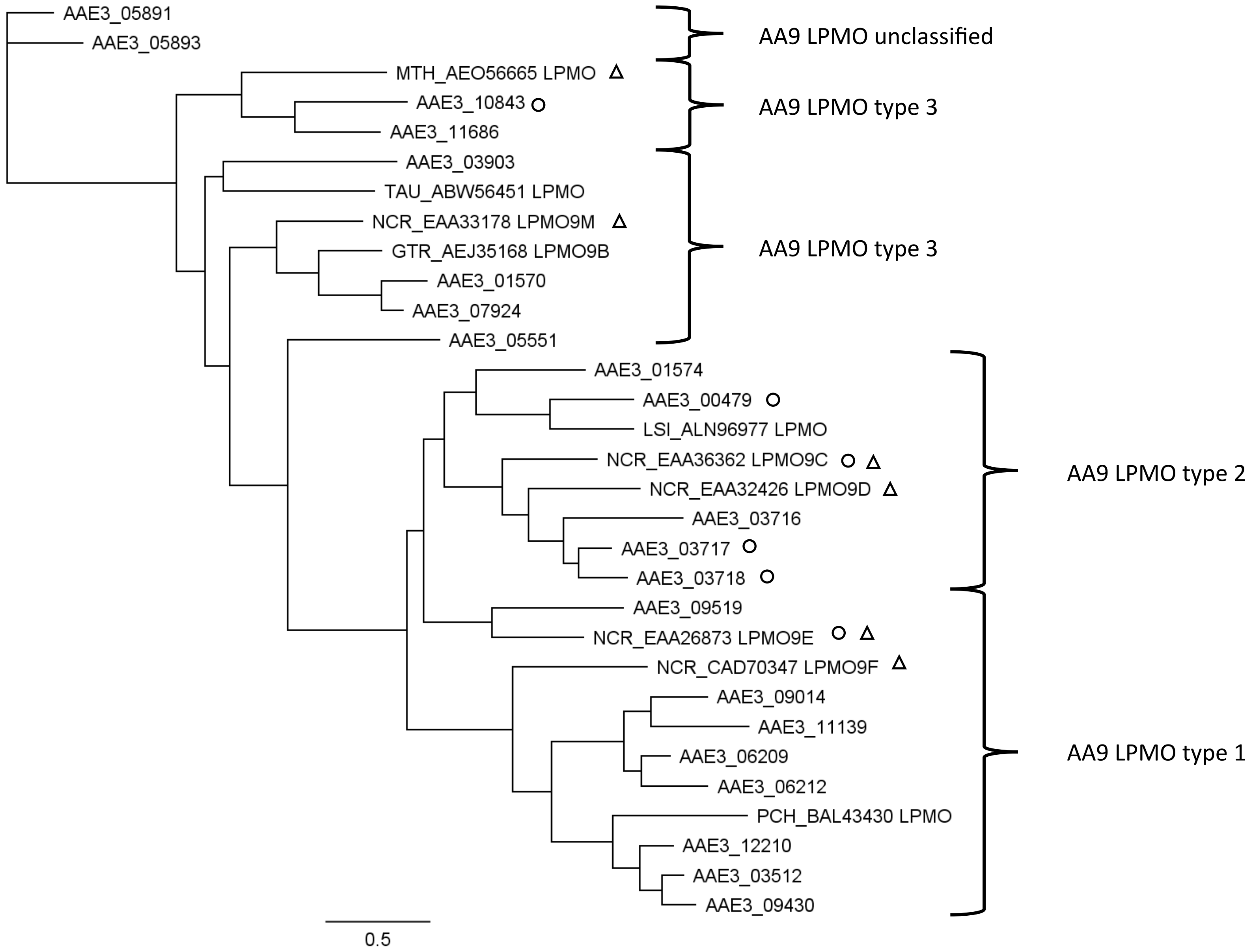

Figure S10 Phylogenetic tree (PhyML) of aligned deduced protein sequences of lytic polysaccharide monooxygenases (LPMO) from *Agrocybe aegerita* AAE-3 (AAE3) and reference protein sequences from *Myceliophthora thermophila* (MTH), *Thermoascus aurantiacus* (TAU), *Neurospora crassa* (NCR), *Gloeophyllum trabeum* (GTR), *Lentinus similis* (LSI) and *Phaneroachaete chrysosporium* (PCH). Protein codes from reference sequences refer to NCBI GenBank (http://www.ncbi.nlm.nih.gov/). A circle marks protein sequences with a C-terminal CBM1 domain. A triangle marks the LPMO determining the type

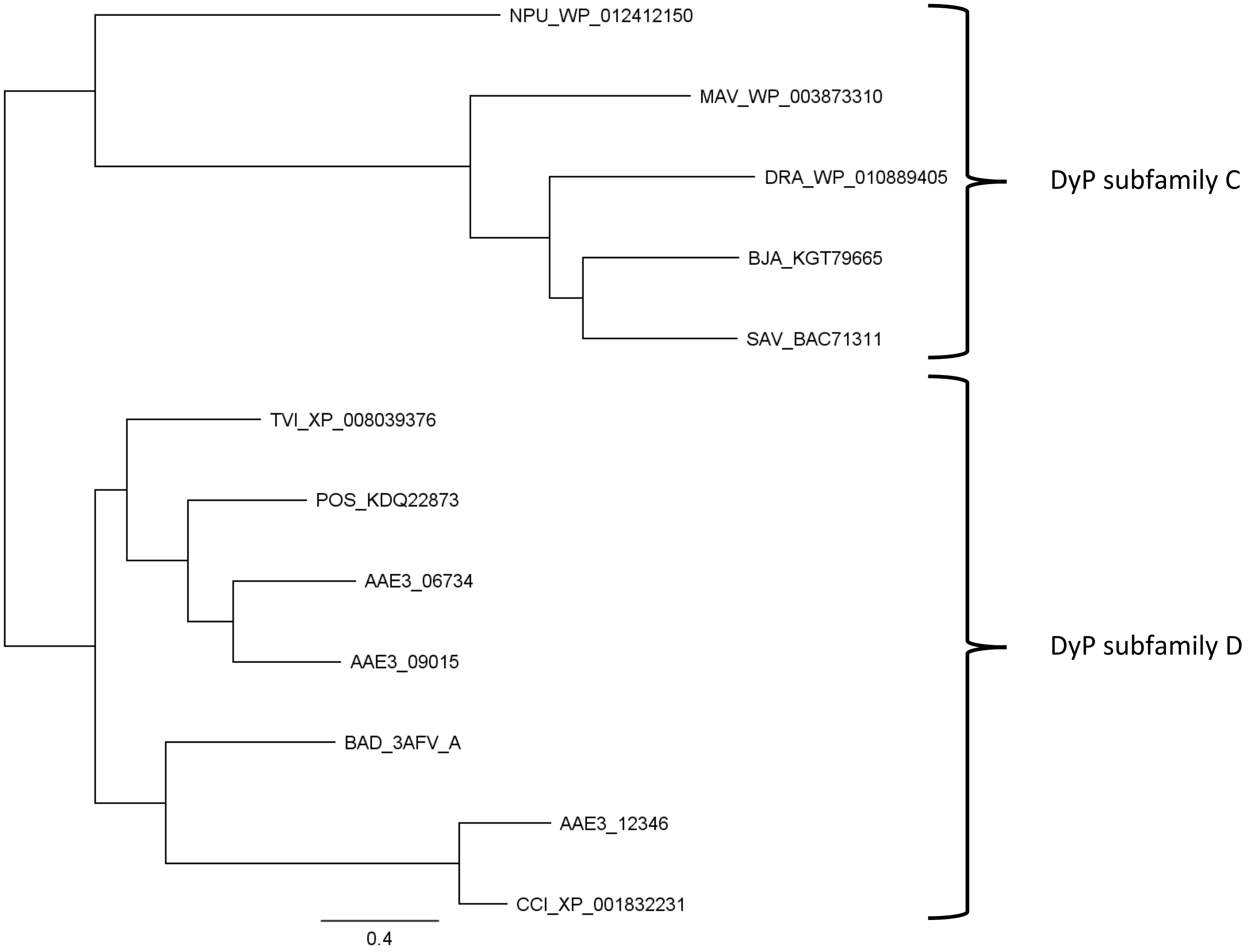

Figure S11 Phylogenetic tree (PhyML) of the *Agrocybe aegerita* AAE-3 (AAE3) dye-decolorizing peroxidases (DyP) and reference sequences from *Nostoc punctiforme* (NPU), *Mycobacterium avium* (MAV), *Deinococcus radiodurans* (DRA), *Bradyrhizobium japonicum* (BJA), *Streptomyces avermitilis* (SAV), *Trametes versicolor* (TVI), *Pleurotus ostreatus* (POS), *Bjerkandera adusta* (BAD) and *Coprinopsis cinerea* (CCI). Protein codes from reference sequences refer to NCBI GenBank (http://www.ncbi.nlm.nih.gov/)

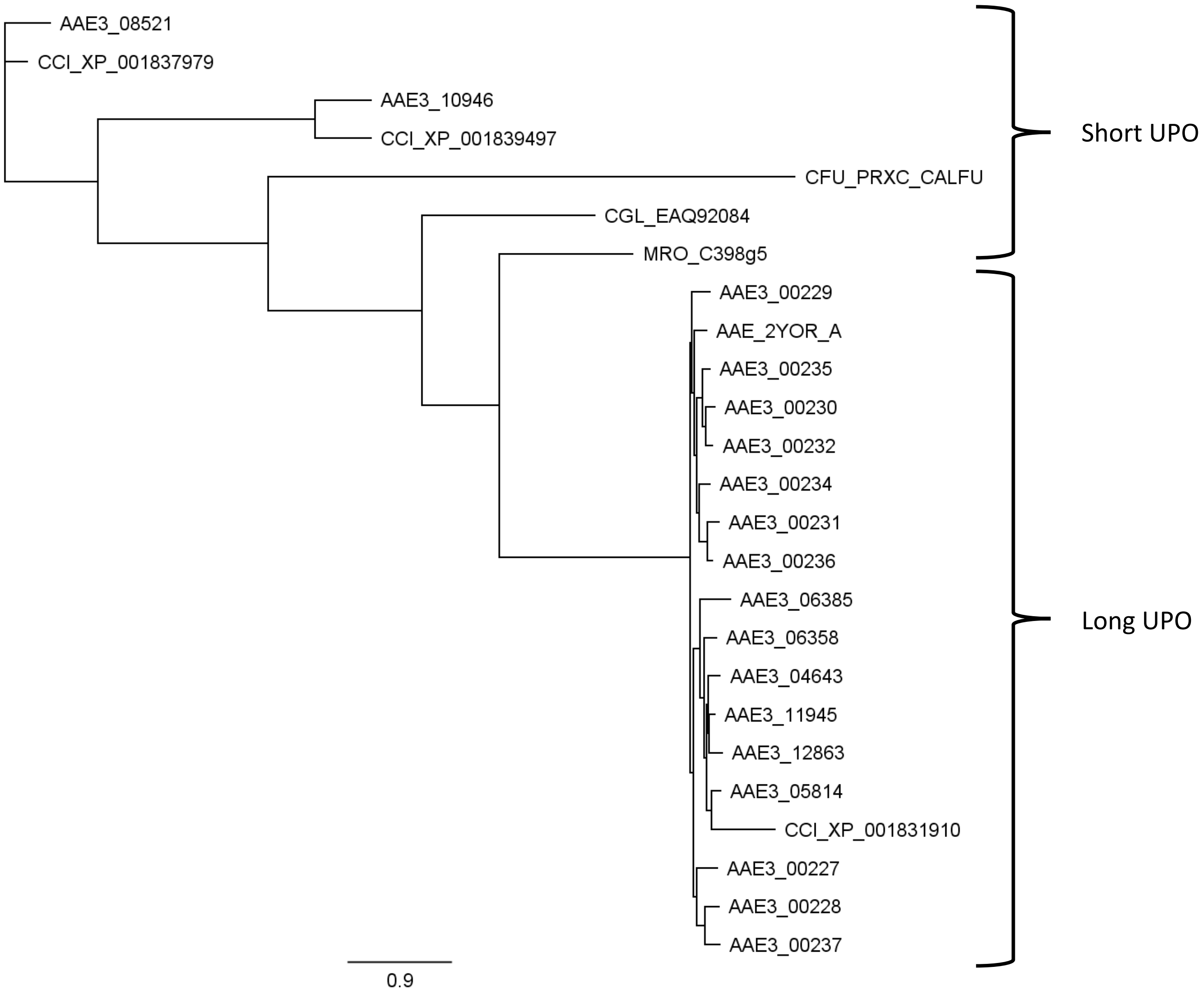

Figure S12 Phylogenetic tree (PhyML) of the *Agrocybe aegerita* AAE-3 (AAE3) unspecific peroxygenases (UPO) and reference sequences from *Coprinopsis cinerea* (CCI), *Caldariomyces fumago* (CFU), *Chaetomium globosum* (CGL), *Marasmius rotula* (MRO, unpublished) and *Agrocybe aegerita* TM A1 (AAE). Protein codes from reference sequences refer to NCBI GenBank (http://www.ncbi.nlm.nih.gov/)

	TVE	GMA	ASU	FME	PCH	CSU	DSQ	PST	PCA	POS	HAN	SHI	VVO	AAE3	CCI	ABI	SCO	JAR	BBO	FPI	PPL	WCO	DSP	GTR	CSU	SLA		
Crystalline cellulose	23	54	43	6	30	17	17	21	27	33	17	17	7	30	44	13	5	24	28	0	0	0	1	1	2	8	CBM1	Carbohydrate-binding module family 1
	1	3	2	2	1	1	1	1	1	3	1	1	0	4	5	1	1	3	3	0	0	0	0	0	2	1	GH6	Glycoside hydrolase family 6
	4	8	8	2	9	3	4	5	5	16	1	3	14	11	6	1	2	5	7	0	0	0	0	0	2	0	GH7	Glycoside hydrolase family 7
	18	19	20	13	15	9	15	14	11	29	10	16	30	21	35	11	22	15	32	4	2	2	0	4	10	5	AA9	Lytic polysaccharide monooxygenase
Lignin	25	10	5	16	15	15	12	10	8	9	8	5	4	8	1	2	0	0	0	0	0	0	0	0	0	0	AA2	Class II peroxidase
	17	32	30	24	27	18	30	19	37	36	29	40	8	25	18	18	18	16	21	15	21	9	8	20	14	8	AA3_2	GMC oxidoreductase
	9	15	8	4	7	3	9	9	6	16	5	8	3	8	6	8	2	4	5	4	3	4	3	2	6	3	AA5_1	Copper radical oxidase
	7	8	0	10	0	7	11	12	0	11	14	15	11	14	17	12	2	1	0	5	2	3	0	4	6	4	AA1_1	Laccase
	4	6	6	3	3	3	4	4	4	4	3	7	5	3	0	5	4	2	3	5	4	5	1	2	5	5	AA3_3	Alcohol oxidase
	0	9	2	0	0	0	4	1	0	3	3	3	2	2	2	3	4	1	4	5	0	0	3	0	0	0	AA7	Glucooligosaccharide oxidase
	2	1	1	1	1	1	1	1	1	1	1	2	0	1	0	1	0	1	1	1	1	1	3	1	1	1	AA1_2	Ferroxidase
	1	1	1	1	1	1	1	1	1	1	1	1	1	0	1	1	1	1	3	0	0	0	0	1	2	2	AA3_1	Cellobiose dehydrogenase
	1	0	3	0	1	0	0	1	0	0	0	0	0	0	0	0	0	1	2	0	0	0	0	1	0	0	AA3_4	Pyranose oxidase
	1	0	7	1	4	1	1	0	8	0	2	3	0	0	0	0	4	1	4	1	1	1	2	0	1	1	AA1_dist	Multicopper oxidase
	1	3	4	3	4	0	1	2	3	2	2	1	2	3	3	4	4	3	1	1	1	1	1	3	2	2	AA6	Bezoquinone reductase
	2	1	2	1	2	2	2	1	2	1	2	2	1	1	6	1	3	2	2	0	0	0	0	0	4	4	AA8	Iron reductase domain
	0	0	0	0	0	0	0	0	0	0	0	0	-	0	-	-	0	2	0	0	0	0	2	3	0	0	AA4	Vanillyl alcohol oxidase
	2	5	11	3	0	0	1	5	1	5	1	2	3	3	4	0	0	1	3	6	0	0	0	0	0	0	DyP	DyP-type peroxidase
	3	0	16	4	3	9	4	8	0	3	4	10	0	18	8	24	3	0	0	4	3	5	6	6	4	3	UPO	Unspecific peroxygenase

Figure S13 Selected CAZy genes present in basidiomycete genomes involved in ligninolytic and cellulolytic degradation. *Agrocybe aegerita* (AAE3); *Auricularia subglabra* (ASU); *Botryobasidium botryosum* (BBO); *Ceriporiopsis subvermispora* (CSU); *Coniophora puteana* (CPU); *Coprinopsis cinerea* (CCI); *Dacryopinax* sp. (DSP); *Dichomitus squalens* (DSQ); *Fomitiporia mediterranea* (FME); *Fomitopsis pinicola* (FPI); *Galerina marginata* (GMA); *Gloeophyllum trabeum* (GTR); *Heterobasidion annosum* (HAN); *Jaapia argillacea* (JAR); *Phanerochaete carnosa* (PCA); *Phanerochaete chrysosporium* (PCH); *Pleurotus ostreatus* (POS); *Postia placenta* (PPL); *Punctularia strigosozonata* (PST); *Schizophyllum commune* (SCO); *Serpula lacrymans* (SLA); *Stereum hirsutum* (SHI); *Trametes versicolor* (TVE); *Volvariella volvacea* (VVO); *Wolfiporia cocos* (WCO). Fungi are categorised by their degradation pattern: white-rot (yellow), litter (green), brown-rot (grey) and unspecified (blue). The figure is based on a figure published by Riley et al. [1] and broadened by data from Kohler et al. [2] for ABI and CCI, Bao et al. [3] for VVO and data from the joint genome institute JGI (https://genome.jgi.doe.gov/programs/fungi/index.jsf)

References

1. Riley R, Salamov AA, Brown DW, Nagy LG, Floudas D, Held BW, et al. Extensive sampling of basidiomycete genomes demonstrates inadequacy of the white-rot/brown-rot paradigm for wood decay fungi. Proc Natl Acad Sci. 2014;111:9923–9928
2. Kohler A, Kuo A, Nagy LG, Morin E, Barry KW, Buscot F, et al. Convergent losses of decay mechanisms and rapid turnover of symbiosis genes in mycorrhizal mutualists. Nat. Genet. 2015;47:410-15.
3. Bao D, Gong M, Zheng H, Chen M, Zhang L, Wang H, et al. Sequencing and Comparative Analysis of the Straw Mushroom (*Volvariella volvacea*) Genome. PLoS One. 2013;8:e58294.

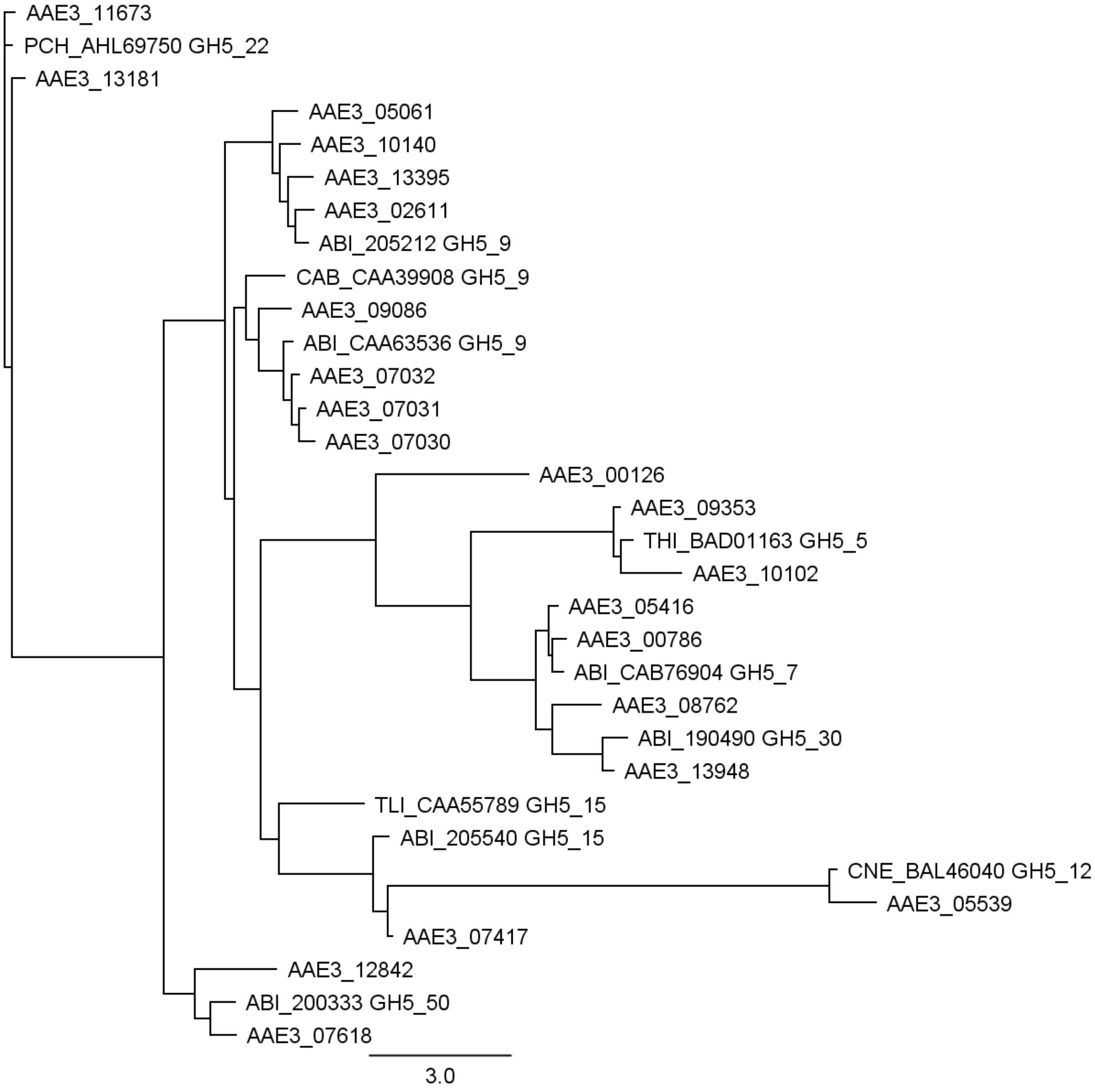

Figure S14 Phylogenetic tree (PhyML) of the *Agrocybe aegerita* AAE-3 (AAE3) genes coding for putative glycoside hydrolases of the CAZy family GH5 and reference sequences from *Phanerochate chrysosporium* (PCH), *Agaricus bisporus* (ABI), *Candida albicans* (CAB), *Trametes hirsuta* (THI), *Trametes lixii* (TLI) and *Cryptococcus neoformans* (CNE). Protein codes from reference sequences refer to NCBI GenBank (http://www.ncbi.nlm.nih.gov/)
