# Supplementary material for: The genome sequence of the commercially cultivated mushroom Agrocybe aegerita reveals a conserved repertoire of fruiting-related genes and a versatile suite of biopolymer-degrading enzymes
Source: BMC Genomics. 2018 Jan 15;19:48. doi: 10.1186/s12864-017-4430-y (PMC5769442; doi:10.1186/s12864-017-4430-y)
Supplement: Supplementary file 2 — Agrocybe aegerita AAE-3 genes in subcategories of GO term “molecular function”. (DOCX 31 kb) [file 12864_2017_4430_MOESM2_ESM.docx]

**Table S1** *Agrocybe aegerita* AAE-3 genes in subcategories of GO term "molecular function"

| **GO-ID** | **GO-term** | **Number of sequences** |
| --- | --- | --- |
| GO:0008152 | metabolic process | 4028 |
| GO:0009987 | cellular process | 3644 |
| GO:0044699 | single-organism process | 2983 |
| GO:0065007 | biological regulation | 1052 |
| GO:0051179 | localisation | 947 |
| GO:0071840 | cellular component organization or biogenesis | 914 |
| GO:0050896 | response to stimulus | 627 |
| GO:0023052 | signaling | 233 |
| GO:0000003 | reproduction | 94 |
| GO:0032502 | developmental process | 59 |
| GO:0051704 | multi-organism process | 47 |
| GO:0022414 | reproductive process | 42 |
| GO:0040007 | growth | 39 |
| GO:0032501 | multicellular organismal process | 14 |
| GO:0001906 | cell killing | 6 |
| GO:0022610 | biological adhesion | 4 |
| GO:0002376 | immune system process | 2 |
| GO:0044848 | biological phase | 2 |
| GO:0040011 | locomotion | 1 |
