# Supplementary material for: The genome sequence of the commercially cultivated mushroom Agrocybe aegerita reveals a conserved repertoire of fruiting-related genes and a versatile suite of biopolymer-degrading enzymes
Source: BMC Genomics. 2018 Jan 15;19:48. doi: 10.1186/s12864-017-4430-y (PMC5769442; doi:10.1186/s12864-017-4430-y)
Supplement: Supplementary file 3 — Agrocybe aegerita AAE-3 genes in subcategories of GO term “biological processes”. (DOCX 31 kb) [file 12864_2017_4430_MOESM3_ESM.docx]

**Table S2** *Agrocybe aegerita* AAE-3 genes in subcategories of GO term "biological processes"

| **GO-ID** | **GO-term** | **Number of sequences** |
| --- | --- | --- |
| GO:0005488 | binding | 4290 |
| GO:0003824 | catalytic activity | 3631 |
| GO:0005215 | transporter activity | 332 |
| GO:0005198 | structural molecule activity | 197 |
| GO:0001071 | nucleic acid binding transcription factor activity | 140 |
| GO:0098772 | molecular function regulator | 126 |
| GO:0016209 | antioxidant activity | 54 |
| GO:0060089 | molecular transducer activity | 48 |
| GO:0000988 | transcription factor activity, protein binding | 43 |
| GO:0009055 | electron carrier activity | 25 |
| GO:0045735 | nutrient reservoir activity | 3 |
| GO:0031386 | protein tag | 3 |
| GO:0045182 | translation regulator activity | 2 |
