# Supplementary material for: The genome sequence of the commercially cultivated mushroom Agrocybe aegerita reveals a conserved repertoire of fruiting-related genes and a versatile suite of biopolymer-degrading enzymes
Source: BMC Genomics. 2018 Jan 15;19:48. doi: 10.1186/s12864-017-4430-y (PMC5769442; doi:10.1186/s12864-017-4430-y)
Supplement: Supplementary file 4 — Agrocybe aegerita AAE-3 genes in subcategories of GO term “cellular components”. (DOCX 31 kb) [file 12864_2017_4430_MOESM4_ESM.docx]

**Table S3** *Agrocybe aegerita* AAE-3 genes in subcategories of GO term "cellular components"

| **GO-ID** | **GO-term** | **Number of sequences** |
| --- | --- | --- |
| GO:0005623 | cell | 2656 |
| GO:0016020 | membrane | 2259 |
| GO:0043226 | organelle | 1986 |
| GO:0032991 | macromolecular complex | 1235 |
| GO:0031974 | membrane-enclosed lumen | 447 |
| GO:0005576 | extracellular region | 89 |
| GO:0019012 | virion | 21 |
| GO:0044215 | other organism | 11 |
| GO:0009295 | nucleoid | 10 |
| GO:0031012 | extracellular matrix | 1 |
