# Supplementary material for: The genome sequence of the commercially cultivated mushroom Agrocybe aegerita reveals a conserved repertoire of fruiting-related genes and a versatile suite of biopolymer-degrading enzymes
Source: BMC Genomics. 2018 Jan 15;19:48. doi: 10.1186/s12864-017-4430-y (PMC5769442; doi:10.1186/s12864-017-4430-y)
Supplement: Supplementary file 5 — Annotation of Agrocybe aegerita AAE-3 genes using InterProScan. (DOCX 254 kb) [file 12864_2017_4430_MOESM5_ESM.docx]

**Table S4** Annotation of *Agrocybe aegerita* AAE-3 genes using InterProScan

| **InterProScan-ID** | **InterProScan family** | **Number of sequences** |
| --- | --- | --- |
| IPR001128 | Cytochrome P450 | 687 |
| IPR002347 | Short-chain dehydrogenase/reductase SDR | 221 |
| IPR002401 | Cytochrome P450, E-class, group I | 140 |
| IPR011701 | Major facilitator superfamily | 130 |
| IPR001019 | Guanine nucleotide binding protein (G-protein), alpha subunit | 80 |
| IPR001338 | Hydrophobin | 69 |
| IPR002293 | Amino acid/polyamine transporter I | 57 |
| IPR002085 | Alcohol dehydrogenase superfamily, zinc-type | 53 |
| IPR001395 | Aldo/keto reductase/potassium channel subunit beta | 49 |
| IPR001765 | Carbonic anhydrase | 47 |
| IPR003663 | Sugar/inositol transporter | 41 |
| IPR006084 | XPG/Rad2 endonuclease | 39 |
| IPR001563 | Peptidase S10, serine carboxypeptidase | 38 |
| IPR004000 | Actin family | 38 |
| IPR005828 | Major facilitator, sugar transporter-like | 37 |
| IPR001382 | Glycoside hydrolase family 47 | 37 |
| IPR029033 | Histidine phosphatase superfamily | 35 |
| IPR001461 | Aspartic peptidase A1 family | 35 |
| IPR022812 | Dynamin superfamily | 33 |
| IPR001722 | Glycoside hydrolase, family 7 | 32 |
| IPR008914 | Phosphatidylethanolamine-binding protein | 31 |
| IPR020471 | Aldo/keto reductase | 30 |
| IPR000164 | Histone H3/CENP-A | 29 |
| IPR004813 | Oligopeptide transporter, OPT superfamily | 28 |
| IPR001806 | Small GTPase superfamily | 28 |
| IPR002403 | Cytochrome P450, E-class, group IV | 26 |
| IPR001757 | P-type ATPase | 26 |
| IPR020636 | Calcium/calmodulin-dependent/calcium-dependent protein kinase | 26 |
| IPR015500 | Peptidase S8, subtilisin-related | 25 |
| IPR013126 | Heat shock protein 70 family | 23 |
| IPR016288 | 1, 4-beta cellobiohydrolase | 22 |
| IPR027640 | Kinesin-like protein | 22 |
| IPR001842 | Peptidase M36, fungalysin | 21 |
| IPR012132 | Glucose-methanol-choline oxidoreductase | 21 |
| IPR005301 | MOB kinase activator family | 21 |
| IPR015655 | Protein phosphatase 2C family | 21 |
| IPR001938 | Thaumatin | 21 |
| IPR005103 | Glycoside hydrolase, family 61 | 21 |
| IPR006035 | Ureohydrolase | 20 |
| IPR026892 | Glycoside hydrolase family 3 | 20 |
| IPR001619 | Sec1-like protein | 18 |
| IPR013078 | Histidine phosphatase superfamily, clade-1 | 18 |
| IPR000425 | Major intrinsic protein | 18 |
| IPR002524 | Cation efflux protein | 18 |
| IPR000277 | Cys/Met metabolism, pyridoxal phosphate-dependent enzyme | 17 |
| IPR001164 | Arf GTPase activating protein | 16 |
| IPR001353 | Proteasome, subunit alpha/beta | 16 |
| IPR016491 | Septin | 16 |
| IPR020849 | Small GTPase superfamily, Ras type | 15 |
| IPR000704 | Casein kinase II, regulatory subunit | 15 |
| IPR006689 | Small GTPase superfamily, ARF/SAR type | 15 |
| IPR008462 | CsbD-like | 14 |
| IPR001360 | Glycoside hydrolase family 1 | 14 |
| IPR024936 | Cyclophilin-type peptidyl-prolyl cis-trans isomerase | 14 |
| IPR005814 | Aminotransferase class-III | 14 |
| IPR003000 | Sirtuin family | 14 |
| IPR011935 | Conserved hypothetical protein CHP02231 | 14 |
| IPR002423 | Chaperonin Cpn60/TCP-1 family | 14 |
| IPR004834 | Fungal chitin synthase | 14 |
| IPR001310 | Histidine triad (HIT) protein | 14 |
| IPR002020 | Citrate synthase | 14 |
| IPR007259 | Gamma-tubulin complex component protein | 14 |
| IPR002067 | Mitochondrial carrier protein | 14 |
| IPR007855 | RNA-dependent RNA polymerase, eukaryotic-type | 13 |
| IPR000648 | Oxysterol-binding protein | 13 |
| IPR000092 | Polyprenyl synthetase | 13 |
| IPR016461 | O-methyltransferase COMT-type | 13 |
| IPR009053 | Prefoldin | 12 |
| IPR008547 | Protein of unknown function DUF829, TMEM53 | 12 |
| IPR002685 | Glycosyl transferase, family 15 | 12 |
| IPR000639 | Epoxide hydrolase-like | 12 |
| IPR000542 | Acyltransferase ChoActase/COT/CPT | 12 |
| IPR000286 | Histone deacetylase superfamily | 12 |
| IPR001499 | GPCR fungal pheromone mating factor, STE3 | 12 |
| IPR001046 | NRAMP family | 12 |
| IPR004835 | Chitin synthase | 12 |
| IPR008521 | Magnesium transporter NIPA | 12 |
| IPR024950 | Dual specificity phosphatase | 12 |
| IPR006539 | P-type ATPase, subfamily IV | 12 |
| IPR012133 | Alpha-hydroxy acid dehydrogenase, FMN-dependent | 11 |
| IPR005629 | Beta-glucan synthesis-associated, SKN1 | 11 |
| IPR007274 | Ctr copper transporter | 11 |
| IPR002220 | DapA-like | 10 |
| IPR009413 | Hemolysin, aegerolysin type | 10 |
| IPR010255 | Haem peroxidase | 10 |
| IPR006130 | Aspartate/ornithine carbamoyltransferase | 10 |
| IPR000218 | Ribosomal protein L14P | 10 |
| IPR006461 | PLAC8 motif-containing protein | 10 |
| IPR000217 | Tubulin | 10 |
| IPR001753 | Crotonase superfamily | 10 |
| IPR023398 | Translation Initiation factor eIF- 4e-like | 10 |
| IPR020946 | Flavin monooxygenase-like | 10 |
| IPR006175 | YjgF/YER057c/UK114 family | 9 |
| IPR000266 | Ribosomal protein S17/S11 | 9 |
| IPR009571 | Membrane protein SUR7/Rim9-like, fungi | 9 |
| IPR001260 | Coproporphyrinogen III oxidase, aerobic | 9 |
| IPR000407 | Nucleoside phosphatase GDA1/CD39 | 9 |
| IPR001130 | TatD family | 9 |
| IPR018150 | Aminoacyl-tRNA synthetase, class II (D/K/N)-like | 9 |
| IPR001734 | Sodium/solute symporter | 9 |
| IPR019901 | Ergot alkaloid biosynthesis protein | 9 |
| IPR001269 | tRNA-dihydrouridine synthase | 9 |
| IPR001544 | Aminotransferase class IV | 9 |
| IPR002523 | Mg2+ transporter protein, CorA-like/Zinc transport protein ZntB | 9 |
| IPR000850 | Adenylate kinase/UMP-CMP kinase | 9 |
| IPR019410 | Lysine methyltransferase | 9 |
| IPR017998 | Chaperone tailless complex polypeptide 1 (TCP-1) | 9 |
| IPR000120 | Amidase | 9 |
| IPR001568 | Ribonuclease T2-like | 9 |
| IPR000743 | Glycoside hydrolase, family 28 | 9 |
| IPR006439 | HAD hydrolase, subfamily IA | 9 |
| IPR025649 | Protein of unknown function DUF4360 | 9 |
| IPR016435 | Diphthamide synthesis DPH1/DPH2 | 9 |
| IPR001661 | Glycoside hydrolase, family 37 | 9 |
| IPR001525 | C-5 cytosine methyltransferase | 9 |
| IPR005599 | GPI mannosyltransferase | 8 |
| IPR002303 | Valine-tRNA ligase | 8 |
| IPR002933 | Peptidase M20 | 8 |
| IPR019591 | Mrp/NBP35 ATP-binding protein | 8 |
| IPR000537 | UbiA prenyltransferase family | 8 |
| IPR001441 | Decaprenyl diphosphate synthase-like | 8 |
| IPR000362 | Fumarate lyase family | 8 |
| IPR002317 | Serine-tRNA ligase, type1 | 8 |
| IPR000558 | Histone H2B | 8 |
| IPR021740 | Velvet factor | 8 |
| IPR002119 | Histone H2A | 8 |
| IPR005822 | Ribosomal protein L13 | 8 |
| IPR006032 | Ribosomal protein S12/S23 | 8 |
| IPR007269 | Isoprenylcysteine carboxyl methyltransferase | 8 |
| IPR018608 | Gti1/Pac2 family | 8 |
| IPR000462 | CDP-alcohol phosphatidyltransferase | 8 |
| IPR010905 | Glycosyl hydrolase, family 88 | 8 |
| IPR002908 | Frataxin/CyaY | 8 |
| IPR005746 | Thioredoxin | 8 |
| IPR003700 | Ketopantoate hydroxymethyltransferase | 8 |
| IPR023333 | Proteasome B-type subunit | 8 |
| IPR002467 | Peptidase M24A, methionine aminopeptidase, subfamily 1 | 8 |
| IPR001040 | Translation Initiation factor eIF- 4e | 8 |
| IPR031127 | E3 ubiquitin ligase RBR family | 8 |
| IPR031107 | Small heat shock protein HSP20 | 8 |
| IPR005468 | Avidin/streptavidin | 8 |
| IPR001892 | Ribosomal protein S13 | 7 |
| IPR006219 | DHAP synthase, class 1 | 7 |
| IPR001905 | Ammonium transporter | 7 |
| IPR001971 | Ribosomal protein S11 | 7 |
| IPR005078 | Peptidase C54 | 7 |
| IPR002616 | tRNA-guanine(15) transglycosylase-like | 7 |
| IPR003738 | SOS response associated peptidase (SRAP) | 7 |
| IPR025799 | Protein arginine N-methyltransferase | 7 |
| IPR002202 | Hydroxymethylglutaryl-CoA reductase, class I/II | 7 |
| IPR021765 | Protein of unknown function DUF3328 | 7 |
| IPR027796 | OTT_1508-like deaminase | 7 |
| IPR001958 | Tetracycline resistance protein TetA/multidrug resistance protein MdtG | 7 |
| IPR023566 | Peptidyl-prolyl cis-trans isomerase, FKBP-type | 7 |
| IPR019791 | Haem peroxidase, animal | 7 |
| IPR000730 | Proliferating cell nuclear antigen, PCNA | 7 |
| IPR001192 | Phosphoinositide phospholipase C family | 7 |
| IPR000511 | Cytochrome c/c1 haem-lyase | 7 |
| IPR002213 | UDP-glucuronosyl/UDP-glucosyltransferase | 7 |
| IPR025340 | Protein of unknown function DUF4246 | 7 |
| IPR006172 | DNA-directed DNA polymerase, family B | 7 |
| IPR002092 | DNA-directed RNA polymerase, phage-type | 7 |
| IPR000789 | Cyclin-dependent kinase, regulatory subunit | 7 |
| IPR007657 | Glycosyltransferase AER61, uncharacterised | 7 |
| IPR005178 | Organic solute transporter subunit alpha/Transmembrane protein 184 | 7 |
| IPR023332 | Proteasome A-type subunit | 7 |
| IPR010126 | Esterase, PHB depolymerase | 7 |
| IPR000322 | Glycoside hydrolase family 31 | 7 |
| IPR000630 | Ribosomal protein S8 | 7 |
| IPR001404 | Heat shock protein Hsp90 family | 7 |
| IPR027725 | Heat shock transcription factor family | 7 |
| IPR006179 | 5'-Nucleotidase/apyrase | 7 |
| IPR001189 | Manganese/iron superoxide dismutase | 7 |
| IPR015902 | Glycoside hydrolase, family 13 | 7 |
| IPR000101 | Gamma-glutamyltranspeptidase | 7 |
| IPR006710 | Glycoside hydrolase, family 43 | 7 |
| IPR019378 | GDP-fucose protein O-fucosyltransferase | 7 |
| IPR001447 | Arylamine N-acetyltransferase | 7 |
| IPR012719 | T-complex protein 1, gamma subunit | 6 |
| IPR005937 | 26S proteasome subunit P45 | 6 |
| IPR000760 | Inositol monophosphatase | 6 |
| IPR002208 | SecY/SEC61-alpha family | 6 |
| IPR031054 | Glutamate carboxypeptidase 2 homologue | 6 |
| IPR001737 | Ribosomal RNA adenine methyltransferase KsgA/Erm | 6 |
| IPR003094 | Fructose-2,6-bisphosphatase | 6 |
| IPR015712 | DNA-directed RNA polymerase, subunit 2 | 6 |
| IPR000043 | Adenosylhomocysteinase | 6 |
| IPR028651 | ING family | 6 |
| IPR001807 | Chloride channel, voltage gated | 6 |
| IPR018466 | Kre9/Knh1 family | 6 |
| IPR002129 | Pyridoxal phosphate-dependent decarboxylase | 6 |
| IPR001645 | Folylpolyglutamate synthetase | 6 |
| IPR004648 | Tetrapeptide transporter, OPT1/isp4 | 6 |
| IPR005522 | Inositol polyphosphate kinase | 6 |
| IPR002504 | NAD kinase | 6 |
| IPR004130 | GPN-loop GTPase | 6 |
| IPR000453 | Chorismate synthase | 6 |
| IPR000589 | Ribosomal protein S15 | 6 |
| IPR004345 | TB2/DP1/HVA22-related protein | 6 |
| IPR002678 | GTP cyclohydrolase 1 type 2/Nif3 | 6 |
| IPR001283 | Cysteine-rich secretory protein, allergen V5/Tpx-1-related | 6 |
| IPR002618 | UDPGP family | 6 |
| IPR006544 | P-type ATPase, subfamily V | 6 |
| IPR033010 | The WD repeat Cdc20/Fizzy family | 6 |
| IPR006314 | Dyp-type peroxidase | 6 |
| IPR005455 | Profilin | 6 |
| IPR006357 | HAD-superfamily hydrolase, subfamily IIA | 6 |
| IPR018499 | Tetraspanin/Peripherin | 6 |
| IPR031327 | Mini-chromosome maintenance protein | 6 |
| IPR015937 | Aconitase/isopropylmalate dehydratase | 6 |
| IPR016763 | Vesicle-associated membrane-protein-associated protein | 6 |
| IPR021134 | Bestrophin/UPF0187 | 6 |
| IPR003197 | Cytochrome b-c1 complex subunit 7 | 6 |
| IPR015679 | Phospholipase D family | 6 |
| IPR000812 | Transcription factor TFIIB | 6 |
| IPR000907 | Lipoxygenase | 6 |
| IPR027073 | 5'-3' exoribonuclease | 6 |
| IPR001564 | Nucleoside diphosphate kinase | 6 |
| IPR008238 | Chorismate mutase, AroQ class, eukaryotic type | 6 |
| IPR032801 | Peroxiredoxin-like FAM213/AAED1 | 6 |
| IPR001944 | Glycoside hydrolase, family 35 | 6 |
| IPR002241 | Glycoside hydrolase, family 27 | 6 |
| IPR001063 | Ribosomal protein L22/L17 | 6 |
| IPR024156 | Small GTPase superfamily, ARF type | 6 |
| IPR006598 | Lipopolysaccharide-modifying protein | 6 |
| IPR001585 | Transaldolase/Fructose-6-phosphate aldolase | 6 |
| IPR000456 | Ribosomal protein L17 | 6 |
| IPR002171 | Ribosomal protein L2 | 6 |
| IPR002848 | Translin | 6 |
| IPR001951 | Histone H4 | 6 |
| IPR004808 | AP endonuclease 1 | 6 |
| IPR002155 | Thiolase | 6 |
| IPR002306 | Tryptophan-tRNA ligase | 6 |
| IPR005024 | Snf7 family | 6 |
| IPR002528 | Multi antimicrobial extrusion protein | 6 |
| IPR018028 | Catalase, mono-functional, haem-containing | 6 |
| IPR001852 | Pyridoxal 5'-phosphate synthase subunit PdxS/SNZ | 6 |
| IPR001733 | Peptidase S26B | 6 |
| IPR001289 | Nuclear transcription factor Y subunit A | 5 |
| IPR003788 | Putative S-adenosyl-L-methionine-dependent methyltransferase MidA | 5 |
| IPR001602 | Uncharacterised protein family UPF0047 | 5 |
| IPR019438 | Potential Queuosine, Q, salvage protein family | 5 |
| IPR002836 | PDCD5-related protein | 5 |
| IPR005810 | Succinyl-CoA ligase, alpha subunit | 5 |
| IPR001848 | Ribosomal protein S10 | 5 |
| IPR004327 | Phosphotyrosyl phosphatase activator, PTPA | 5 |
| IPR000652 | Triosephosphate isomerase | 5 |
| IPR007248 | Mpv17/PMP22 | 5 |
| IPR000056 | Ribulose-phosphate 3-epimerase-like | 5 |
| IPR006939 | SNF5/SMARCB1/INI1 | 5 |
| IPR001248 | Purine-cytosine permease | 5 |
| IPR000754 | Ribosomal protein S9 | 5 |
| IPR006034 | Asparaginase/glutaminase | 5 |
| IPR015507 | Ribosomal RNA large subunit methyltransferase E | 5 |
| IPR000304 | Pyrroline-5-carboxylate reductase | 5 |
| IPR002164 | Nucleosome assembly protein (NAP) | 5 |
| IPR002019 | Urease, beta subunit | 5 |
| IPR002695 | AICARFT/IMPCHase bienzyme | 5 |
| IPR002124 | Cytochrome c oxidase, subunit Vb | 5 |
| IPR001114 | Adenylosuccinate synthetase | 5 |
| IPR006789 | Actin-related protein 2/3 complex subunit 5 | 5 |
| IPR004242 | Transposon, En/Spm-like | 5 |
| IPR001648 | Ribosomal protein S18 | 5 |
| IPR022684 | Peptidase C2, calpain family | 5 |
| IPR007603 | Choline transporter-like | 5 |
| IPR033177 | Phosphatidylserine decarboxylase | 5 |
| IPR007271 | Nucleotide-sugar transporter | 5 |
| IPR002769 | Translation initiation factor IF6 | 5 |
| IPR006762 | Gtr1/RagA G protein | 5 |
| IPR001684 | Ribosomal protein L27 | 5 |
| IPR002634 | BolA protein | 5 |
| IPR002905 | tRNA methyltransferase, Trm1 | 5 |
| IPR000560 | Histidine phosphatase superfamily, clade-2 | 5 |
| IPR003719 | Phenazine biosynthesis PhzF protein | 5 |
| IPR002196 | Glycoside hydrolase, family 24 | 5 |
| IPR009976 | Exocyst complex component Sec10-like | 5 |
| IPR012664 | Conserved hypothetical protein CHP02452 | 5 |
| IPR001902 | SLC26A/SulP transporter | 5 |
| IPR006043 | Xanthine/uracil/vitamin C permease | 5 |
| IPR001731 | Delta-aminolevulinic acid dehydratase | 5 |
| IPR001891 | Malic oxidoreductase | 5 |
| IPR004516 | Histidine-tRNA ligase/ATP phosphoribosyltransferase regulatory subunit | 5 |
| IPR001576 | Phosphoglycerate kinase | 5 |
| IPR000960 | Flavin monooxygenase FMO | 5 |
| IPR002043 | Uracil-DNA glycosylase | 5 |
| IPR016292 | Epoxide hydrolase | 5 |
| IPR001854 | Ribosomal protein L29 | 5 |
| IPR014816 | tRNA (1-methyladenosine) methyltransferase catalytic subunit Gcd14 | 5 |
| IPR007568 | RTA-like protein | 5 |
| IPR000269 | Copper amine oxidase | 5 |
| IPR001406 | Pseudouridine synthase I, TruA | 5 |
| IPR019465 | Conserved oligomeric Golgi complex subunit 5 | 5 |
| IPR017446 | Polyprenyl synthetase-related | 5 |
| IPR024768 | Meiosis arrest female protein 1 | 5 |
| IPR002161 | Pyridoxal 5'-phosphate synthase subunit PdxT/SNO | 5 |
| IPR000246 | Peptidase T2, asparaginase 2 | 5 |
| IPR002180 | 6,7-dimethyl-8-ribityllumazine synthase | 5 |
| IPR020818 | GroES chaperonin family | 5 |
| IPR023209 | D-amino-acid oxidase | 5 |
| IPR011603 | 2-oxoglutarate dehydrogenase E1 component | 5 |
| IPR004033 | UbiE/COQ5 methyltransferase | 5 |
| IPR008049 | DNA replication licensing factor Mcm6 | 5 |
| IPR004205 | Cytochrome b-c1 complex subunit 8 | 5 |
| IPR007941 | Protein of unknown function DUF726 | 5 |
| IPR000771 | Ketose-bisphosphate aldolase, class-II | 5 |
| IPR001253 | Translation initiation factor 1A (eIF-1A) | 5 |
| IPR007204 | Actin-related protein 2/3 complex subunit 3 | 5 |
| IPR005197 | Glycoside hydrolase family 71 | 5 |
| IPR000552 | Ribosomal protein L44e | 5 |
| IPR001991 | Sodium:dicarboxylate symporter | 5 |
| IPR003673 | CoA-transferase family III | 5 |
| IPR002026 | Urease, gamma/gamma-beta subunit | 5 |
| IPR032854 | Alkylated DNA repair protein alkB homologue 3 | 4 |
| IPR011107 | Protein phosphatase inhibitor | 4 |
| IPR002876 | Transcriptional regulator TACO1-like | 4 |
| IPR022801 | Ribosomal protein S4/S9 | 4 |
| IPR025705 | Beta-hexosaminidase | 4 |
| IPR005809 | Succinyl-CoA synthetase, beta subunit | 4 |
| IPR013248 | Shr3 amino acid permease chaperone | 4 |
| IPR010036 | Magnesium-dependent phosphatase-1, eukaryotic/archaeal type | 4 |
| IPR003195 | Transcription initiation factor IID, 18kDa subunit | 4 |
| IPR007704 | Mannosyltransferase, DXD | 4 |
| IPR001708 | Membrane insertase OXA1/ALB3/YidC | 4 |
| IPR032816 | SNARE associated Golgi protein | 4 |
| IPR001972 | Stomatin family | 4 |
| IPR005654 | ATPase, AFG1-like | 4 |
| IPR007122 | Villin/Gelsolin | 4 |
| IPR001865 | Ribosomal protein S2 | 4 |
| IPR001844 | Chaperonin Cpn60 | 4 |
| IPR015699 | DNA-directed RNA pol I, largest subunit | 4 |
| IPR002283 | Isopenicillin N synthase | 4 |
| IPR004522 | Asparagine-tRNA ligase | 4 |
| IPR000631 | ATP-dependent (S)-NAD(P)H-hydrate dehydratase | 4 |
| IPR004254 | AdipoR/Haemolysin-III-related | 4 |
| IPR002773 | Deoxyhypusine synthase | 4 |
| IPR000796 | Aspartate/other aminotransferase | 4 |
| IPR002610 | Peptidase S54, rhomboid | 4 |
| IPR001804 | Isocitrate and isopropylmalate dehydrogenases family | 4 |
| IPR017861 | Kae1/TsaD family | 4 |
| IPR006254 | Isocitrate lyase | 4 |
| IPR000889 | Glutathione peroxidase | 4 |
| IPR001085 | Serine hydroxymethyltransferase | 4 |
| IPR001328 | Peptidyl-tRNA hydrolase | 4 |
| IPR003721 | Pantoate-beta-alanine ligase | 4 |
| IPR004023 | Mago nashi protein | 4 |
| IPR027065 | Lon protease | 4 |
| IPR000911 | Ribosomal protein L11/L12 | 4 |
| IPR005615 | Glutathione synthase, eukaryotic | 4 |
| IPR005946 | Ribose-phosphate diphosphokinase | 4 |
| IPR012131 | Histidinol dehydrogenase | 4 |
| IPR001977 | Dephospho-CoA kinase | 4 |
| IPR026749 | Transmembrane protein 135 | 4 |
| IPR002737 | MEMO1 family | 4 |
| IPR029001 | Inosine triphosphate pyrophosphatase-like | 4 |
| IPR002305 | Aminoacyl-tRNA synthetase, class Ic | 4 |
| IPR000807 | Imidazoleglycerol-phosphate dehydratase | 4 |
| IPR000131 | ATPase, F1 complex, gamma subunit | 4 |
| IPR016639 | Glutathione S-transferase (GST) | 4 |
| IPR017795 | Aromatic prenyltransferase, DMATS type | 4 |
| IPR001621 | Fungal ligninase | 4 |
| IPR007129 | Ubiquinol-cytochrome c chaperone, CBP3 | 4 |
| IPR027075 | Cleavage and polyadenylation specificity factor subunit 2 | 4 |
| IPR002778 | Signal recognition particle, SRP19 subunit | 4 |
| IPR000271 | Ribosomal protein L34 | 4 |
| IPR005936 | Peptidase, FtsH | 4 |
| IPR004226 | Tubulin binding cofactor A | 4 |
| IPR015414 | Transmembrane protein TMEM64/TMEM41 | 4 |
| IPR005990 | Inosine-5'-monophosphate dehydrogenase | 4 |
| IPR018203 | GDP dissociation inhibitor | 4 |
| IPR004712 | Na+/H+ antiporter, fungi | 4 |
| IPR007523 | NDUFAF3/Mth938 domain-containing protein | 4 |
| IPR004601 | UV-endonuclease UvdE | 4 |
| IPR003492 | Batten's disease protein Cln3 | 4 |
| IPR014780 | tRNA pseudouridine synthase II, TruB | 4 |
| IPR011078 | Uncharacterised protein family UPF0001 | 4 |
| IPR002777 | Prefoldin beta-like | 4 |
| IPR000711 | ATPase, OSCP/delta subunit | 4 |
| IPR001719 | AP endonuclease 2 | 4 |
| IPR005061 | Vacuolar protein sorting-associated protein Ist1 | 4 |
| IPR005226 | UPF0014 family | 4 |
| IPR001210 | Ribosomal protein S17e | 4 |
| IPR012110 | Thiamine pyrophosphate (TPP)-dependent enzyme | 4 |
| IPR016125 | Peptidase C15, pyroglutamyl peptidase I-like | 4 |
| IPR004203 | Cytochrome c oxidase subunit IV family | 4 |
| IPR000163 | Prohibitin | 4 |
| IPR002301 | Isoleucine-tRNA ligase | 4 |
| IPR000109 | Proton-dependent oligopeptide transporter family | 4 |
| IPR000228 | RNA 3'-terminal phosphate cyclase | 4 |
| IPR002259 | Equilibrative nucleoside transporter | 4 |
| IPR005033 | YEATS | 4 |
| IPR002042 | Uricase | 4 |
| IPR000702 | Ribosomal protein L6 | 4 |
| IPR026983 | Dynein heavy chain | 4 |
| IPR000481 | GPCR fungal pheromone B alpha receptor | 4 |
| IPR005485 | Ribosomal protein L5 eukaryotic/L18 archaeal | 4 |
| IPR000289 | Ribosomal protein S28e | 4 |
| IPR000417 | Hydroxyethylthiazole kinase | 4 |
| IPR002935 | O-methyltransferase, family 3 | 4 |
| IPR025533 | Protein of unknown function DUF4419 | 4 |
| IPR027165 | Condensin complex subunit 3 | 4 |
| IPR000358 | Ribonucleotide reductase small subunit | 4 |
| IPR019956 | Ubiquitin | 4 |
| IPR002554 | Protein phosphatase 2A, regulatory B subunit, B56 | 4 |
| IPR000581 | Dihydroxy-acid/6-phosphogluconate dehydratase | 4 |
| IPR013233 | Glycosylphosphatidylinositol-mannosyltransferase I, PIG-X/PBN1 | 4 |
| IPR000692 | Fibrillarin | 4 |
| IPR028831 | DNA mismatch repair protein Pms1/Pms2 | 4 |
| IPR004856 | Glycosyl transferase, ALG6/ALG8 | 4 |
| IPR012173 | U3 small nucleolar ribonucleoprotein complex, subunit Mpp10 | 4 |
| IPR011400 | Eukaryotic translation initiation factor 3 subunit B | 4 |
| IPR006155 | Machado-Joseph disease protein MJD | 4 |
| IPR000612 | Proteolipid membrane potential modulator | 4 |
| IPR006818 | Histone chaperone ASF1-like | 4 |
| IPR001282 | Glucose-6-phosphate dehydrogenase | 4 |
| IPR007590 | CWC16 protein | 4 |
| IPR018492 | Ribosomal protein L7Ae/L8/Nhp2 family | 4 |
| IPR002480 | DAHP synthetase, class II | 4 |
| IPR000811 | Glycosyl transferase, family 35 | 4 |
| IPR008576 | Alpha-N-methyltransferase NTM1 | 4 |
| IPR015222 | Mitochondrial matrix Mmp37 | 4 |
| IPR001278 | Arginine-tRNA ligase | 4 |
| IPR015720 | TMP21-related | 4 |
| IPR009836 | Glycine-rich domain-containing protein-like | 4 |
| IPR003689 | Zinc/iron permease | 4 |
| IPR026791 | Dedicator of cytokinesis | 4 |
| IPR014758 | Methionyl-tRNA synthetase | 4 |
| IPR006206 | Mevalonate/galactokinase | 4 |
| IPR023186 | Inosine/uridine-preferring nucleoside hydrolase | 4 |
| IPR006285 | Ubiquitin-like modifier-activating enzyme Atg7 | 4 |
| IPR001312 | Hexokinase | 4 |
| IPR006274 | Carbamoyl-phosphate synthase, small subunit | 4 |
| IPR004855 | Transcription factor IIA, alpha/beta subunit | 4 |
| IPR013860 | Protein of unknown function DUF1752, fungi | 4 |
| IPR007757 | MT-A70-like | 4 |
| IPR015433 | Phosphatidylinositol Kinase | 4 |
| IPR000649 | Initiation factor 2B-related | 4 |
| IPR021154 | H/ACA ribonucleoprotein complex, subunit Gar1 | 4 |
| IPR000941 | Enolase | 4 |
| IPR001266 | Ribosomal protein S19e | 4 |
| IPR001515 | Ribosomal protein L32e | 4 |
| IPR008162 | Inorganic pyrophosphatase | 4 |
| IPR002307 | Tyrosine-tRNA ligase | 4 |
| IPR012099 | Midasin | 4 |
| IPR010111 | Kynureninase | 4 |
| IPR001372 | Dynein light chain, type 1/2 | 4 |
| IPR003213 | Cytochrome c oxidase, subunit VIb | 4 |
| IPR005045 | CDC50/LEM3 family | 4 |
| IPR003377 | Cornichon | 4 |
| IPR012912 | Plasmid pRiA4b, Orf3 | 4 |
| IPR000988 | Ribosomal protein L24e-related | 4 |
| IPR029915 | ISWI family | 4 |
| IPR011268 | Purine nucleoside phosphorylase | 4 |
| IPR000814 | TATA-box binding protein | 4 |
| IPR003204 | Cytochrome c oxidase, subunit Va/VI | 4 |
| IPR003196 | Transcription initiation factor IIF, beta subunit | 4 |
| IPR003084 | Histone deacetylase | 4 |
| IPR001672 | Phosphoglucose isomerase (PGI) | 4 |
| IPR001129 | Membrane-associated, eicosanoid/glutathione metabolism (MAPEG) protein | 4 |
| IPR033247 | Transketolase family | 4 |
| IPR000054 | Ribosomal protein L31e | 4 |
| IPR003923 | Transcription initiation factor TFIID, 23-30kDa subunit | 4 |
| IPR013933 | Chromatin-remodelling complex, RSC SWI/SNF subunit Rsc7/Swp82 | 4 |
| IPR019405 | Lactonase, 7-bladed beta propeller | 4 |
| IPR004123 | Dim1 family | 4 |
| IPR001714 | Peptidase M24, methionine aminopeptidase | 4 |
| IPR003397 | Mitochondrial inner membrane translocase subunit Tim17/Tim22/Tim23/peroxisomal protein PMP24 | 4 |
| IPR001557 | L-lactate/malate dehydrogenase | 3 |
| IPR008862 | T-complex 11 | 3 |
| IPR008010 | Tapt1 family | 3 |
| IPR024535 | Pectate lyase superfamily protein | 3 |
| IPR015659 | Proline oxidase family | 3 |
| IPR012170 | TFIIH subunit Ssl1/p44 | 3 |
| IPR013005 | 50S ribosomal protein uL4 | 3 |
| IPR012808 | Conserved hypothetical protein CHP02453 | 3 |
| IPR004790 | Isocitrate dehydrogenase NADP-dependent | 3 |
| IPR032914 | VAM6/VPS39 family | 3 |
| IPR009049 | Argininosuccinate lyase | 3 |
| IPR000243 | Peptidase T1A, proteasome beta-subunit | 3 |
| IPR000778 | Cytochrome b245, heavy chain | 3 |
| IPR007194 | Transport protein particle (TRAPP) component | 3 |
| IPR024671 | Autophagy-related protein 22-like | 3 |
| IPR007255 | Conserved oligomeric Golgi complex subunit 8 | 3 |
| IPR004977 | Ribosomal protein S25 | 3 |
| IPR003337 | Trehalose-phosphatase | 3 |
| IPR002320 | Threonine-tRNA ligase, class IIa | 3 |
| IPR019317 | Brain protein I3 | 3 |
| IPR001096 | Peptidase C13, legumain | 3 |
| IPR019587 | Polyketide cyclase/dehydrase | 3 |
| IPR000977 | DNA ligase, ATP-dependent | 3 |
| IPR001392 | Clathrin adaptor, mu subunit | 3 |
| IPR008220 | Homoserine acetyltransferase | 3 |
| IPR002699 | ATPase, V1 complex, subunit D | 3 |
| IPR000447 | FAD-dependent glycerol-3-phosphate dehydrogenase | 3 |
| IPR007763 | NADH dehydrogenase [ubiquinone] 1 alpha subcomplex subunit 12 | 3 |
| IPR000682 | Protein-L-isoaspartate(D-aspartate) O-methyltransferase | 3 |
| IPR002410 | Peptidase S33 | 3 |
| IPR028553 | Neurofibromin | 3 |
| IPR010089 | Flavoprotein WrbA | 3 |
| IPR000009 | Protein phosphatase 2A regulatory subunit PR55 | 3 |
| IPR027108 | Pre-mRNA-processing factor 6/Prp1/STA1 | 3 |
| IPR004888 | Glycoside hydrolase family 63 | 3 |
| IPR006298 | GTP-binding protein TypA | 3 |
| IPR003021 | Rad1/Rec1/Rad17 | 3 |
| IPR028134 | Ubiquitin carboxyl-terminal hydrolase USP4/12 | 3 |
| IPR022043 | Chromatin assembly factor 1 subunit A | 3 |
| IPR005848 | Urease, alpha subunit | 3 |
| IPR017141 | Peptidase M20, carboxypeptidase S | 3 |
| IPR027127 | Actin-related protein 10 | 3 |
| IPR002312 | Aspartyl/Asparaginyl-tRNA synthetase, class IIb | 3 |
| IPR013935 | TRAPP II complex, Trs120 | 3 |
| IPR004400 | Urease accessory protein UreG | 3 |
| IPR000465 | XPA | 3 |
| IPR001094 | Flavodoxin-like | 3 |
| IPR003428 | Mitochondrial glycoprotein | 3 |
| IPR001783 | Lumazine-binding protein | 3 |
| IPR001377 | Ribosomal protein S6e | 3 |
| IPR006110 | RNA polymerase, subunit omega/K/RPB6 | 3 |
| IPR003701 | DNA double-strand break repair protein Mre11 | 3 |
| IPR007594 | RFT1 | 3 |
| IPR003817 | Phosphatidylserine decarboxylase-related | 3 |
| IPR024706 | Peroxiredoxin, AhpC-type | 3 |
| IPR000245 | V-ATPase proteolipid subunit | 3 |
| IPR001952 | Alkaline phosphatase | 3 |
| IPR005570 | RNA polymerase, Rpb8 | 3 |
| IPR007918 | Mitochondrial distribution/morphology family 35/apoptosis | 3 |
| IPR007143 | Vacuolar protein sorting-associated, VPS28 | 3 |
| IPR008218 | ATPase, V1 complex, subunit F | 3 |
| IPR001474 | GTP cyclohydrolase I | 3 |
| IPR006840 | ChaC-like protein | 3 |
| IPR011613 | Glycoside hydrolase family 15/Phosphorylase b kinase regulatory chain family | 3 |
| IPR016643 | 26S proteasome regulatory complex, non-ATPase subcomplex, Rpn1 subunit | 3 |
| IPR008183 | Aldose 1-/Glucose-6-phosphate 1-epimerase | 3 |
| IPR007783 | Eukaryotic translation initiation factor 3 subunit D | 3 |
| IPR002056 | Protein import receptor MAS20 | 3 |
| IPR000892 | Ribosomal protein S26e | 3 |
| IPR016900 | Alpha-2-glucosyltransferase Alg10 | 3 |
| IPR001384 | Peptidase M35, deuterolysin | 3 |
| IPR005065 | Platelet-activating factor acetylhydrolase | 3 |
| IPR027663 | Dynactin subunit 1 | 3 |
| IPR005016 | Serine incorporator/TMS membrane protein | 3 |
| IPR001233 | tRNA-splicing ligase, RtcB | 3 |
| IPR023267 | RNA (C5-cytosine) methyltransferase | 3 |
| IPR000554 | Ribosomal protein S7e | 3 |
| IPR011876 | Isopentenyl-diphosphate delta-isomerase, type 1 | 3 |
| IPR017334 | Eukaryotic translation initiation factor 3 subunit G | 3 |
| IPR026872 | Protein farnesyltransferase subunit beta | 3 |
| IPR016650 | Eukaryotic translation initiation factor 3 subunit E | 3 |
| IPR006722 | Trafficking protein particle complex subunit 2 | 3 |
| IPR001697 | Pyruvate kinase | 3 |
| IPR004788 | Ribose 5-phosphate isomerase, type A | 3 |
| IPR024873 | Ectonucleotide pyrophosphatase/phosphodiesterase family | 3 |
| IPR007518 | Protein of unknown function DUF544 | 3 |
| IPR024960 | Phosphatidyl-N-methylethanolamine/N-methyltransferase | 3 |
| IPR002677 | Ribosomal protein L32p | 3 |
| IPR003231 | Acyl carrier protein (ACP) | 3 |
| IPR027141 | Like-Sm (LSM) domain containing protein, LSm4/SmD1/SmD3 | 3 |
| IPR003732 | D-aminoacyl-tRNA deacylase DTD | 3 |
| IPR010721 | Protein of unknown function DUF1295 | 3 |
| IPR015413 | Methionyl/Leucyl tRNA synthetase | 3 |
| IPR000439 | Ribosomal protein L15e | 3 |
| IPR007653 | Signal peptidase 22kDa subunit | 3 |
| IPR001930 | Peptidase M1, alanine aminopeptidase/leukotriene A4 hydrolase | 3 |
| IPR005772 | ATPase, V1 complex, subunit F, eukaryotic | 3 |
| IPR006849 | Elongator complex subunit Iki3 | 3 |
| IPR001272 | Phosphoenolpyruvate carboxykinase, ATP-utilising | 3 |
| IPR004776 | Membrane transport protein | 3 |
| IPR025602 | BCP1 family | 3 |
| IPR009784 | Protein of unknown function DUF1349 | 3 |
| IPR024111 | Peroxisomal targeting signal 1 receptor family | 3 |
| IPR004589 | DNA helicase, ATP-dependent, RecQ type | 3 |
| IPR003733 | Thiamine phosphate synthase | 3 |
| IPR002082 | Aspartate carbamoyltransferase | 3 |
| IPR002051 | Haem oxygenase | 3 |
| IPR016305 | Mannose-6-phosphate isomerase | 3 |
| IPR006165 | Ku70 | 3 |
| IPR001171 | Ergosterol biosynthesis ERG4/ERG24 | 3 |
| IPR022137 | Protein of unknown function DUF3669, zinc finger protein | 3 |
| IPR008257 | Renal dipeptidase family | 3 |
| IPR028589 | AdoMet-dependent rRNA methyltransferase, Spb1 | 3 |
| IPR004480 | Monothiol glutaredoxin-related | 3 |
| IPR000890 | Aliphatic acid kinase, short-chain | 3 |
| IPR004598 | Transcription factor TFIIH subunit p52/Tfb2 | 3 |
| IPR003358 | tRNA (guanine-N-7) methyltransferase, Trmb type | 3 |
| IPR009772 | Cell division cycle protein 123 | 3 |
| IPR031155 | Urea active transporter | 3 |
| IPR008689 | ATPase, F0 complex, subunit D, mitochondrial | 3 |
| IPR000529 | Ribosomal protein S6 | 3 |
| IPR002745 | Phosphotransferase KptA/Tpt1 | 3 |
| IPR000114 | Ribosomal protein L16 | 3 |
| IPR000165 | Glucoamylase | 3 |
| IPR000380 | DNA topoisomerase, type IA | 3 |
| IPR033268 | Structural maintenance of chromosomes protein 5/6 | 3 |
| IPR001857 | Ribosomal protein L19 | 3 |
| IPR002891 | Adenylyl-sulfate kinase | 3 |
| IPR005345 | PHF5-like | 3 |
| IPR002821 | Hydantoinase/oxoprolinase | 3 |
| IPR005786 | Branched-chain amino acid aminotransferase II | 3 |
| IPR005631 | Flavinator of succinate dehydrogenase | 3 |
| IPR013906 | Eukaryotic translation initiation factor 3 subunit J | 3 |
| IPR017774 | Bicupin, oxalate decarboxylase/oxidase | 3 |
| IPR021054 | Cell wall mannoprotein 1 | 3 |
| IPR004353 | Vacuolar fusion protein MON1 | 3 |
| IPR027746 | Probable tubulin-tyrosine ligase | 3 |
| IPR023473 | AMMECR1 | 3 |
| IPR007015 | DNA polymerase V | 3 |
| IPR006685 | Mechanosensitive ion channel MscS | 3 |
| IPR018939 | Autophagy-related protein 27 | 3 |
| IPR024909 | Cysteinyl-tRNA synthetase/mycothiol ligase | 3 |
| IPR005708 | Homogentisate 1,2-dioxygenase | 3 |
| IPR009348 | Nitrogen permease regulator 2 | 3 |
| IPR007537 | tRNAHis guanylyltransferase Thg1 | 3 |
| IPR001884 | Translation elongation factor IF5A | 3 |
| IPR002591 | Type I phosphodiesterase/nucleotide pyrophosphatase/phosphate transferase | 3 |
| IPR009160 | Acyl-CoA desaturase, haem/steroid binding domain-containing | 3 |
| IPR005294 | ATPase, F1 complex, alpha subunit | 3 |
| IPR001698 | F-actin-capping protein subunit beta | 3 |
| IPR002297 | DNA-directed DNA-polymerase, family A, mitochondria | 3 |
| IPR007266 | Endoplasmic reticulum oxidoreductin 1 | 3 |
| IPR001147 | Ribosomal protein L21e | 3 |
| IPR004907 | ATPase, V1 complex, subunit C | 3 |
| IPR003120 | Transcription factor Ste12 | 3 |
| IPR026030 | Purine-cytosine permease Fcy2/21/22 | 3 |
| IPR000490 | Glycoside hydrolase family 17 | 3 |
| IPR009161 | ATP-dependent 6-phosphofructokinase, eukaryotic-type | 3 |
| IPR001748 | G10 protein | 3 |
| IPR007229 | Nicotinate phosphoribosyltransferase family | 3 |
| IPR002008 | DNA polymerase family X, beta-like | 3 |
| IPR000898 | Indoleamine 2,3-dioxygenase | 3 |
| IPR001727 | Gdt1 family | 3 |
| IPR028364 | Ribosomal protein L1/ribosomal biogenesis protein | 3 |
| IPR003874 | CDC45 family | 3 |
| IPR003210 | Signal recognition particle, SRP14 subunit | 3 |
| IPR000398 | Thymidylate synthase | 3 |
| IPR001141 | Ribosomal protein L27e | 3 |
| IPR004418 | Homoaconitase, mitochondrial | 3 |
| IPR005880 | Ribosomal protein L2, bacterial/organellar-type | 3 |
| IPR002674 | Ribosomal protein L37ae | 3 |
| IPR018105 | Translationally controlled tumour protein | 3 |
| IPR001139 | Glycoside hydrolase family 30 | 3 |
| IPR012093 | Pirin | 3 |
| IPR008195 | Ribosomal protein L34Ae | 3 |
| IPR003698 | Lipoyl synthase | 3 |
| IPR012094 | tRNA(Ile)-lysidine synthase | 3 |
| IPR002222 | Ribosomal protein S19/S15 | 3 |
| IPR002680 | Alternative oxidase | 3 |
| IPR024734 | Magnesium-dependent phosphatase-1, eukaryotic type | 3 |
| IPR026739 | AP complex subunit beta | 3 |
| IPR002133 | S-adenosylmethionine synthetase | 3 |
| IPR002326 | Cytochrome c1 | 3 |
| IPR011691 | Vesicle transport protein, SFT2-like | 3 |
| IPR004083 | Regulatory associated protein of TOR | 3 |
| IPR005378 | Vacuolar protein sorting-associated protein 35 | 3 |
| IPR002028 | Tryptophan synthase, alpha chain | 3 |
| IPR006534 | P-type ATPase, subfamily IIIA | 3 |
| IPR015876 | Fatty acid desaturase, type 1, core | 3 |
| IPR000740 | GrpE nucleotide exchange factor | 3 |
| IPR017476 | UDP-glucose/GDP-mannose dehydrogenase | 3 |
| IPR010329 | 3-hydroxyanthranilic acid dioxygenase | 3 |
| IPR005995 | Phosphoglycerate mutase, 2,3-bisphosphoglycerate-independent | 3 |
| IPR001976 | Ribosomal protein S24e | 3 |
| IPR005656 | MmgE/PrpD | 3 |
| IPR000308 | 14-3-3 protein | 3 |
| IPR015257 | Repressor of RNA polymerase III transcription Maf1 | 3 |
| IPR002587 | Myo-inositol-1-phosphate synthase | 3 |
| IPR006276 | Cobalamin-independent methionine synthase | 3 |
| IPR002637 | Ham1-like protein | 3 |
| IPR016849 | Histone acetyltransferase Rtt109 | 3 |
| IPR014764 | Defective-in-cullin neddylation protein | 3 |
| IPR012724 | Chaperone DnaJ | 3 |
| IPR004134 | Peptidase C1B, bleomycin hydrolase | 3 |
| IPR002815 | Spo11/DNA topoisomerase VI subunit A | 3 |
| IPR020795 | Origin recognition complex, subunit 3 | 3 |
| IPR009637 | Lung seven transmembrane receptor-like | 3 |
| IPR015362 | WIBG family | 3 |
| IPR008384 | Actin-related protein 2/3 complex subunit 4 | 3 |
| IPR006709 | Small-subunit processome, Utp14 | 3 |
| IPR020831 | Glyceraldehyde/Erythrose phosphate dehydrogenase family | 3 |
| IPR032979 | Cytosolic endo-beta-N-acetylglucosaminidase | 3 |
| IPR007286 | EAP30 | 3 |
| IPR008758 | Peptidase S28 | 3 |
| IPR001834 | NADH:cytochrome b5 reductase (CBR) | 3 |
| IPR007233 | Trafficking protein particle complex subunit | 3 |
| IPR002189 | F-actin-capping protein subunit alpha | 3 |
| IPR011687 | Ribosome biogenesis protein Nop53/GLTSCR2 | 3 |
| IPR008313 | Metal-independent alpha-mannosidase | 3 |
| IPR003737 | N-acetylglucosaminyl phosphatidylinositol deacetylase-related | 3 |
| IPR008628 | Golgi phosphoprotein 3 | 3 |
| IPR004765 | Niemann-Pick C type protein | 3 |
| IPR014311 | Guanine deaminase | 3 |
| IPR024880 | COPII coat assembly protein, Sec16 | 3 |
| IPR008733 | Peroxisomal biogenesis factor 11 | 3 |
| IPR013025 | Ribosomal protein L25/L23 | 3 |
| IPR001398 | Macrophage migration inhibitory factor | 3 |
| IPR004165 | Coenzyme A transferase family I | 3 |
| IPR004574 | Alkylated DNA repair protein AlkB | 3 |
| IPR008045 | DNA replication licensing factor Mcm2 | 3 |
| IPR027005 | Glycosyltransferase 39-like | 3 |
| IPR001015 | Ferrochelatase | 3 |
| IPR005914 | Acetoacetyl-CoA synthase | 3 |
| IPR004299 | Membrane bound O-acyl transferase, MBOAT | 3 |
| IPR004721 | Dihydroorotase homodimeric type | 3 |
| IPR001780 | Ribosomal protein L35A | 3 |
| IPR001106 | Aromatic amino acid lyase | 3 |
| IPR002468 | Peptidase M24A, methionine aminopeptidase, subfamily 2 | 3 |
| IPR002698 | 5-formyltetrahydrofolate cyclo-ligase | 3 |
| IPR003226 | Metal-dependent protein hydrolase | 3 |
| IPR013023 | Ketol-acid reductoisomerase | 3 |
| IPR000915 | 60S ribosomal protein L6E | 3 |
| IPR000307 | Ribosomal protein S16 | 3 |
| IPR026082 | ABC transporter A, ABCA | 3 |
| IPR004307 | TspO/MBR-related protein | 3 |
| IPR000406 | Rho protein GDP-dissociation inhibitor | 3 |
| IPR002495 | Glycosyl transferase, family 8 | 3 |
| IPR016635 | Adaptor protein complex, sigma subunit | 3 |
| IPR007225 | Exocyst complex subunit Sec15-like | 3 |
| IPR005756 | Ribosomal protein L26/L24P, eukaryotic/archaeal | 3 |
| IPR001656 | Pseudouridine synthase, TruD | 3 |
| IPR000860 | Porphobilinogen deaminase | 3 |
| IPR002813 | Arginine biosynthesis protein ArgJ | 3 |
| IPR007241 | Autophagy-related protein 9 | 3 |
| IPR002023 | NADH-quinone oxidoreductase subunit E-like | 3 |
| IPR002470 | Peptidase S9A, prolyl oligopeptidase | 3 |
| IPR002313 | Lysine-tRNA ligase, class II | 3 |
| IPR007533 | Cytochrome c oxidase assembly protein CtaG/Cox11 | 3 |
| IPR008011 | Complex 1 LYR protein | 3 |
| IPR007305 | Vesicle transport protein, Got1/SFT2-like | 3 |
| IPR013657 | UAA transporter | 3 |
| IPR007822 | Lanthionine synthetase C-like | 3 |
| IPR022312 | DNA polymerase family X | 3 |
| IPR010347 | Tyrosyl-DNA phosphodiesterase I | 3 |
| IPR000718 | Peptidase M13 | 3 |
| IPR008594 | Scavenger mRNA decapping enzyme DcpS/DCS2 | 3 |
| IPR017439 | Amidohydrolase | 3 |
| IPR013256 | Chromatin SPT2 | 2 |
| IPR002490 | V-type ATPase, V0 complex, 116kDa subunit family | 2 |
| IPR033031 | SCC2/Nipped-B family | 2 |
| IPR005349 | TMEM14 family | 2 |
| IPR007811 | DNA-directed RNA polymerase III subunit RPC4 | 2 |
| IPR019263 | Inorganic phosphate transporter Pho88 | 2 |
| IPR017714 | Methylthioribulose-1-phosphate dehydratase | 2 |
| IPR002671 | Ribosomal protein L22e | 2 |
| IPR010014 | Diphthamide synthesis DHP2, eukaryotic | 2 |
| IPR006113 | 6-phosphogluconate dehydrogenase, decarboxylating | 2 |
| IPR000705 | Galactokinase | 2 |
| IPR000641 | CbxX/CfqX | 2 |
| IPR006968 | Root UVB sensitive family | 2 |
| IPR003840 | DNA helicase | 2 |
| IPR024990 | Anaphase-promoting complex subunit 1 | 2 |
| IPR007369 | Peptidase A22B, signal peptide peptidase | 2 |
| IPR007245 | GPI transamidase component PIG-T | 2 |
| IPR027246 | Eukaryotic porin/Tom40 | 2 |
| IPR001567 | Peptidase M3A/M3B | 2 |
| IPR000223 | Peptidase S26A, signal peptidase I | 2 |
| IPR013958 | DASH complex subunit Dad1 | 2 |
| IPR010334 | mRNA-decapping enzyme subunit 1 | 2 |
| IPR004584 | DNA repair protein Rad50, eukaryotes | 2 |
| IPR007220 | Origin recognition complex, subunit 2 | 2 |
| IPR001354 | Mandelate racemase/muconate lactonizing enzyme/methylaspartate ammonia-lyase | 2 |
| IPR000559 | Formate-tetrahydrofolate ligase, FTHFS | 2 |
| IPR032642 | DNA mismatch repair protein Msh2 | 2 |
| IPR006773 | 26S proteasome complex ubiquitin receptor, subunit Rpn13 | 2 |
| IPR007318 | Phospholipid methyltransferase | 2 |
| IPR009145 | U2 auxiliary factor small subunit | 2 |
| IPR006223 | Glycine cleavage system T protein | 2 |
| IPR010490 | Conserved oligomeric Golgi complex subunit 6 | 2 |
| IPR004344 | Tubulin-tyrosine ligase/Tubulin polyglutamylase | 2 |
| IPR004600 | TFIIH subunit Tfb4/p34 | 2 |
| IPR011160 | Sphingomyelin phosphodiesterase | 2 |
| IPR002843 | ATPase, V0 complex, c/d subunit | 2 |
| IPR010286 | Ribosomal RNA large subunit methyltransferase F-like | 2 |
| IPR009269 | NF-kappa-B-activating protein/UPF0396 | 2 |
| IPR013926 | CGI121/TPRKB | 2 |
| IPR004416 | tRNA uridine 5-carboxymethylaminomethyl modification enzyme MnmG | 2 |
| IPR008564 | Protein of unknown function DUF846, eukaryotic | 2 |
| IPR021819 | Protein of unknown function DUF3402 | 2 |
| IPR009311 | Interferon alpha-inducible protein 6/27 | 2 |
| IPR006808 | ATPase, F0 complex, subunit G, mitochondrial | 2 |
| IPR018794 | Uncharacterised protein family UPF0538 | 2 |
| IPR016049 | RNA polymerase Rpc34-like | 2 |
| IPR005469 | Avidin | 2 |
| IPR030518 | Glucose N-acetyltransferase 1 | 2 |
| IPR008111 | RNA-binding motif protein 8 | 2 |
| IPR005343 | Nucleolar complex protein 2 | 2 |
| IPR000870 | Homoserine kinase | 2 |
| IPR003020 | Bicarbonate transporter, eukaryotic | 2 |
| IPR004625 | Pyridoxine kinase | 2 |
| IPR003445 | Cation transporter | 2 |
| IPR027121 | Vacuolar protein sorting-associated protein 33 | 2 |
| IPR004313 | Acireductone dioxygenase ARD family | 2 |
| IPR017407 | Serine/threonine-protein kinase Rio1 | 2 |
| IPR009737 | Thioredoxin-like ferredoxin | 2 |
| IPR004551 | Diphthine synthase | 2 |
| IPR006809 | TAFII28-like protein | 2 |
| IPR017383 | Actin-related protein 2/3 complex subunit 1 | 2 |
| IPR007632 | Anoctamin | 2 |
| IPR010073 | Phosphoribosylformylglycinamidine synthase | 2 |
| IPR006973 | Pre-mRNA-splicing factor Cwf15/Cwc15 | 2 |
| IPR008570 | ESCRT-II complex, vps25 subunit | 2 |
| IPR014052 | DNA primase, small subunit, eukaryotic/archaeal | 2 |
| IPR012486 | N1221-like | 2 |
| IPR004240 | Nonaspanin (TM9SF) | 2 |
| IPR010960 | Flavocytochrome c | 2 |
| IPR008352 | Mitogen-activated protein (MAP) kinase, p38 | 2 |
| IPR016437 | MCT-1/Tma20 | 2 |
| IPR008467 | Dynein 1 light intermediate chain | 2 |
| IPR004304 | Acetamidase/Formamidase | 2 |
| IPR007023 | Ribosomal biogenesis regulatory protein | 2 |
| IPR006383 | HAD-superfamily hydrolase, subfamily IB, PSPase-like | 2 |
| IPR008699 | NADH dehydrogenase [ubiquinone] 1 beta subcomplex subunit 8 | 2 |
| IPR004499 | Proline-tRNA ligase, class IIa, archaeal-type | 2 |
| IPR008584 | Protein of unknown function DUF866, eukaryotic | 2 |
| IPR005721 | Ribosomal protein L22/L17, eukaryotic/archaeal | 2 |
| IPR023600 | Folylpolyglutamate synthase, eukaryota | 2 |
| IPR007217 | Per1-like | 2 |
| IPR012445 | Autophagy-related protein 101 | 2 |
| IPR005011 | SNU66/SART1 family | 2 |
| IPR005940 | Anthranilate phosphoribosyl transferase | 2 |
| IPR015519 | Serine/threonine-protein kinase ATM | 2 |
| IPR005841 | Alpha-D-phosphohexomutase superfamily | 2 |
| IPR025332 | Protein of unknown function DUF4238 | 2 |
| IPR002041 | Ran GTPase | 2 |
| IPR007290 | Arv1 protein | 2 |
| IPR029940 | AzgA purine transporter | 2 |
| IPR005365 | Nitrogen permease regulator 3 | 2 |
| IPR010139 | Imidazole glycerol phosphate synthase, subunit H | 2 |
| IPR004854 | Ubiquitin fusion degradation protein UFD1 | 2 |
| IPR000872 | Tafazzin | 2 |
| IPR027521 | U6 snRNA phosphodiesterase Usb1 | 2 |
| IPR006138 | NADH-ubiquinone oxidoreductase, 20 Kd subunit | 2 |
| IPR033146 | CnAP1 peptidase | 2 |
| IPR005935 | Diphosphomevalonate/phosphomevalonate decarboxylase | 2 |
| IPR005314 | Peptidase C50, separase | 2 |
| IPR007252 | Nuclear pore protein 84/107 | 2 |
| IPR010414 | FRG1-like | 2 |
| IPR002315 | Glycyl-tRNA synthetase | 2 |
| IPR004524 | Aspartate-tRNA ligase, bacterial/mitochondrial-type | 2 |
| IPR022885 | NAD(P)H-quinone oxidoreductase subunit D/H | 2 |
| IPR001193 | Membrane-bound transcription factor site-2 protease | 2 |
| IPR008291 | Glucoamylase, starch-binding | 2 |
| IPR024166 | Ribosomal RNA assembly KRR1 | 2 |
| IPR017770 | RNA 3'-terminal phosphate cyclase type 1 | 2 |
| IPR007213 | Methyltransferase Ppm1/Ppm2/Tcmp | 2 |
| IPR001985 | S-adenosylmethionine decarboxylase | 2 |
| IPR026913 | Methyltransferase-like protein 24 | 2 |
| IPR004575 | Cdk-activating kinase assembly factor MAT1/Tfb3 | 2 |
| IPR006111 | Archaeal RpoK/eukaryotic RPB6 RNA polymerase subunit | 2 |
| IPR010301 | Nucleolar, Nop52 | 2 |
| IPR010280 | (Uracil-5)-methyltransferase family | 2 |
| IPR010856 | Protein of unknown function DUF1479 | 2 |
| IPR008506 | Protein of unknown function DUF788, TMEM208 | 2 |
| IPR022042 | snRNA-activating protein complex, subunit 3 | 2 |
| IPR000011 | Ubiquitin/SUMO-activating enzyme E1 | 2 |
| IPR013962 | DASH complex subunit Dam1 | 2 |
| IPR012423 | Chromatin modification-related protein Eaf7/MRGBP | 2 |
| IPR009244 | Mediator complex, subunit Med7 | 2 |
| IPR008636 | Hook-related protein family | 2 |
| IPR004908 | ATPase, V1 complex, subunit H | 2 |
| IPR000979 | Phosphodiesterase MJ0936/Vps29 | 2 |
| IPR024881 | T-cell immunomodulatory protein | 2 |
| IPR004870 | Nucleoporin, Nup155-like | 2 |
| IPR005887 | Alpha-1,2-mannosidase, putative | 2 |
| IPR000396 | Cyclic-AMP phosphodiesterase, class-II | 2 |
| IPR005706 | Ribosomal protein S2, bacteria/mitochondria/plastid | 2 |
| IPR007325 | Kynurenine formamidase | 2 |
| IPR004396 | Ribosome-binding ATPase YchF/Obg-like ATPase 1 | 2 |
| IPR005336 | Mitochondrial pyruvate carrier | 2 |
| IPR007018 | Mediator complex, subunit Med6 | 2 |
| IPR004640 | Co-chaperone Hsc20 | 2 |
| IPR028343 | Fructose-1,6-bisphosphatase | 2 |
| IPR016438 | ATP-dependent RNA helicase Ski2 | 2 |
| IPR018627 | Elongation complex protein 6 | 2 |
| IPR004730 | Transaldolase type 1 | 2 |
| IPR009346 | GRIM-19 | 2 |
| IPR005707 | Ribosomal protein S2, eukaryotic/archaeal | 2 |
| IPR013178 | Histone acetyltransferase Rtt109/CBP | 2 |
| IPR019325 | NEDD4/Bsd2 | 2 |
| IPR006168 | Glycerol-3-phosphate dehydrogenase, NAD-dependent | 2 |
| IPR005922 | Phenylalanine ammonia-lyase | 2 |
| IPR026070 | Tyrosine-protein phosphatase CDC14 | 2 |
| IPR012846 | Acetolactate synthase, large subunit, biosynthetic | 2 |
| IPR004430 | 3-isopropylmalate dehydratase, large subunit | 2 |
| IPR007342 | Pseudouridine-5'-phosphate glycosidase | 2 |
| IPR006813 | Glycosyl transferase, family 17 | 2 |
| IPR010733 | Protein of unknown function DUF1308 | 2 |
| IPR026740 | AP-3 complex subunit beta | 2 |
| IPR003154 | S1/P1 nuclease | 2 |
| IPR004127 | Prefoldin alpha-like | 2 |
| IPR021840 | Protein of unknown function DUF3433 | 2 |
| IPR019140 | Mini-chromosome maintenance complex-binding protein | 2 |
| IPR005399 | Potassium channel, voltage-dependent, beta subunit, KCNAB-related | 2 |
| IPR002684 | Biotin synthase/Biotin biosynthesis bifunctional protein BioAB | 2 |
| IPR000672 | Tetrahydrofolate dehydrogenase/cyclohydrolase | 2 |
| IPR010109 | Citrate synthase, eukaryotic-type | 2 |
| IPR007873 | Glycosyltransferase, ALG3 | 2 |
| IPR005366 | Uncharacterised protein family UPF0172 | 2 |
| IPR005334 | Tctex-1 | 2 |
| IPR007834 | DSS1/SEM1 | 2 |
| IPR014371 | Sterol O-acyltransferase, ACAT/DAG/ARE types | 2 |
| IPR030155 | Ferric/cupric reductase transmembrane component 7 | 2 |
| IPR008276 | Concentrative nucleoside transporter | 2 |
| IPR019407 | Cytoplasmic tRNA 2-thiolation protein 2 | 2 |
| IPR027256 | P-type ATPase, subfamily IB | 2 |
| IPR007232 | DNA repair protein Rad52/59/22 | 2 |
| IPR007740 | Ribosomal protein L49/IMG2 | 2 |
| IPR005062 | SAC3/GANP/THP3 | 2 |
| IPR002140 | Ribosome maturation protein Sdo1/SBDS | 2 |
| IPR008631 | Glycogen synthase | 2 |
| IPR003782 | Copper chaperone SCO1/SenC | 2 |
| IPR009292 | rRNA biogenesis protein RRP36 | 2 |
| IPR018094 | Thymidylate kinase | 2 |
| IPR007828 | Inositol oxygenase | 2 |
| IPR004802 | tRNA pseudouridine synthase B family | 2 |
| IPR011566 | Ubiquinone biosynthesis protein Coq7 | 2 |
| IPR010097 | Malate dehydrogenase, type 1 | 2 |
| IPR006214 | Bax inhibitor 1-related | 2 |
| IPR007203 | ORMDL family | 2 |
| IPR001790 | Ribosomal protein L10P | 2 |
| IPR004429 | Isopropylmalate dehydrogenase | 2 |
| IPR021850 | Symplekin/Pta1 | 2 |
| IPR019585 | 26S proteasome regulatory subunit Rpn7/COP9 signalosome complex subunit 1 | 2 |
| IPR009653 | Protein kish | 2 |
| IPR002809 | Protein of unknown function DUF106, transmembrane | 2 |
| IPR007276 | Nucleolar protein 14 | 2 |
| IPR012879 | Protein of unknown function DUF1682 | 2 |
| IPR028427 | Peptide methionine sulfoxide reductase | 2 |
| IPR018034 | KRR1 interacting protein 1 | 2 |
| IPR033317 | Transcription-associated protein 1 | 2 |
| IPR000996 | Clathrin light chain | 2 |
| IPR001047 | Ribosomal protein S8e | 2 |
| IPR016641 | Nascent polypeptide-associated complex subunit alpha | 2 |
| IPR007188 | Actin-related protein 2/3 complex subunit 2 | 2 |
| IPR011171 | Glia maturation factor | 2 |
| IPR033100 | Exosome complex component RRP45 | 2 |
| IPR007243 | Atg6/Beclin | 2 |
| IPR001241 | DNA topoisomerase, type IIA | 2 |
| IPR016661 | Prefoldin, subunit 4 | 2 |
| IPR000659 | Pyridoxamine 5'-phosphate oxidase | 2 |
| IPR005855 | Glucosamine-fructose-6-phosphate aminotransferase, isomerising | 2 |
| IPR005593 | Xylulose 5-phosphate/Fructose 6-phosphate phosphoketolase | 2 |
| IPR003437 | Glycine dehydrogenase (decarboxylating) | 2 |
| IPR007599 | Derlin | 2 |
| IPR005248 | Probable nicotinate-nucleotide adenylyltransferase | 2 |
| IPR000744 | NSF attachment protein | 2 |
| IPR007905 | Emopamil-binding protein | 2 |
| IPR002167 | Graves disease carrier protein | 2 |
| IPR007268 | Rad9/Ddc1 | 2 |
| IPR005554 | Nrap protein | 2 |
| IPR004540 | Translation elongation factor EFG/EF2 | 2 |
| IPR007133 | RNA polymerase II associated factor Paf1 | 2 |
| IPR024193 | Ku80 | 2 |
| IPR009643 | Heat shock factor binding 1 | 2 |
| IPR007785 | Anamorsin | 2 |
| IPR031120 | WD repeat HIR1 | 2 |
| IPR023562 | Clp protease proteolytic subunit /Translocation-enhancing protein TepA | 2 |
| IPR017252 | Dynein regulator LIS1 | 2 |
| IPR004923 | Iron permease FTR1/Fip1/EfeU | 2 |
| IPR016346 | Guanine nucleotide-binding protein, beta subunit | 2 |
| IPR014397 | L-2-aminoadipate reductase | 2 |
| IPR004523 | Aspartyl-tRNA synthetase, archaeal-type | 2 |
| IPR028018 | Protein of unknown function DUF4646 | 2 |
| IPR006811 | RNA polymerase II subunit A | 2 |
| IPR026569 | Ribosomal protein L28/L24 | 2 |
| IPR018791 | UV radiation resistance protein/autophagy-related protein 14 | 2 |
| IPR033689 | NAD-dependent formate dehydrogenase | 2 |
| IPR023208 | NADPH-cytochrome P450 reductase | 2 |
| IPR002453 | Beta tubulin | 2 |
| IPR016624 | Uncharacterised conserved protein UCP014753 | 2 |
| IPR005715 | Glutamate 5-kinase/delta-1-pyrroline-5-carboxylate synthase | 2 |
| IPR012908 | GPI inositol-deacylase PGAP1-like | 2 |
| IPR002452 | Alpha tubulin | 2 |
| IPR010308 | TRP-like family | 2 |
| IPR001381 | 3-dehydroquinate dehydratase type I | 2 |
| IPR011419 | ATP12, ATPase F1F0-assembly protein | 2 |
| IPR000235 | Ribosomal protein S5/S7 | 2 |
| IPR003171 | Methylenetetrahydrofolate reductase | 2 |
| IPR006252 | Malate synthase A | 2 |
| IPR001380 | Ribosomal protein L13e | 2 |
| IPR007919 | Uncharacterised protein family UPF0220 | 2 |
| IPR007146 | Sas10/Utp3/C1D | 2 |
| IPR017144 | Peptidase M20A, amidohydrolase, predicted | 2 |
| IPR027524 | Eukaryotic translation initiation factor 3 subunit H | 2 |
| IPR022085 | Protein of unknown function DUF3632 | 2 |
| IPR024224 | DENND6 | 2 |
| IPR010357 | Protein of unknown function DUF953, thioredoxin-like | 2 |
| IPR005164 | Allantoicase | 2 |
| IPR010678 | Digestive organ expansion factor, predicted | 2 |
| IPR024704 | Structural maintenance of chromosomes protein | 2 |
| IPR022210 | Transcription activator GCR1-like domain | 2 |
| IPR002136 | Ribosomal protein L4/L1e | 2 |
| IPR006235 | O-acetylhomoserine/O-acetylserine sulfhydrylase | 2 |
| IPR004129 | Glycerophosphoryl diester phosphodiesterase | 2 |
| IPR006264 | 3-phosphoshikimate 1-carboxyvinyltransferase | 2 |
| IPR002738 | RNase P subunit p30 | 2 |
| IPR008417 | B-cell receptor-associated protein 29/31 | 2 |
| IPR026825 | Vacuole morphology and inheritance protein 14 | 2 |
| IPR012725 | Chaperone DnaK | 2 |
| IPR010378 | Protein of unknown function DUF974 | 2 |
| IPR007482 | Protein-tyrosine phosphatase-like, PTPLA | 2 |
| IPR016534 | Vacuolar protein sorting-associated protein 16 | 2 |
| IPR004932 | Retrieval of early ER protein Rer1 | 2 |
| IPR010699 | Protein of unknown function DUF1275 | 2 |
| IPR000146 | Fructose-1,6-bisphosphatase class 1 | 2 |
| IPR004140 | Exocyst complex protein Exo70 | 2 |
| IPR002654 | Glycosyl transferase, family 25 | 2 |
| IPR006042 | Xanthine/uracil permease | 2 |
| IPR007991 | RNA polymerase I specific transcription initiation factor RRN3 | 2 |
| IPR001898 | Sodium/sulphate symporter | 2 |
| IPR004403 | Peptide chain release factor eRF1/aRF1 | 2 |
| IPR029529 | Transcription factor IIIB subunit Brf1 | 2 |
| IPR006708 | Pex19 protein | 2 |
| IPR008685 | Centromere protein Mis12 | 2 |
| IPR011555 | V-ATPase proteolipid subunit C, eukaryotic | 2 |
| IPR010218 | NADH dehydrogenase, subunit C | 2 |
| IPR010729 | Ribosomal protein L47, mitochondrial | 2 |
| IPR021047 | Mannosyltransferase 1, CMT1 | 2 |
| IPR009286 | Inositol-pentakisphosphate 2-kinase | 2 |
| IPR007304 | TAP42-like protein | 2 |
| IPR000924 | Glutamyl/glutaminyl-tRNA synthetase | 2 |
| IPR002415 | H/ACA ribonucleoprotein complex, subunit Nhp2, eukaryote | 2 |
| IPR007242 | Ubiquitin-like protein Atg12 | 2 |
| IPR019540 | Phosphatidylinositol-glycan biosynthesis class S protein | 2 |
| IPR012720 | T-complex protein 1, eta subunit | 2 |
| IPR005861 | Histidinol-phosphate aminotransferase family | 2 |
| IPR007265 | Conserved oligomeric Golgi complex, subunit 3 | 2 |
| IPR006822 | Coatomer, epsilon subunit | 2 |
| IPR007955 | Bystin | 2 |
| IPR004541 | Translation elongation factor EFTu/EF1A, bacterial/organelle | 2 |
| IPR016460 | Coatomer beta subunit (COPB1) | 2 |
| IPR016812 | Protein phosphatase methylesterase, eukaryotic | 2 |
| IPR033688 | RNA cytidine acetyltransferase NAT10 | 2 |
| IPR008610 | Eukaryotic rRNA processing | 2 |
| IPR027105 | U4/U6 small nuclear ribonucleoprotein Prp31 | 2 |
| IPR006062 | Histidine biosynthesis | 2 |
| IPR011786 | Sulphite reductase (NADPH) hemoprotein, beta subunit | 2 |
| IPR016817 | Mannose-P-dolichol utilization defect 1 protein | 2 |
| IPR002454 | Gamma tubulin | 2 |
| IPR013260 | mRNA splicing factor SYF2 | 2 |
| IPR016092 | FeS cluster insertion protein | 2 |
| IPR010326 | Exocyst complex component Sec6 | 2 |
| IPR012255 | Electron transfer flavoprotein, beta subunit | 2 |
| IPR008050 | DNA replication licensing factor Mcm7 | 2 |
| IPR005910 | Histone acetyltransferase ELP3 | 2 |
| IPR001369 | PNP/MTAP phosphorylase | 2 |
| IPR026699 | Exosome complex RNA-binding protein 1/RRP40/RRP4 | 2 |
| IPR013878 | Mo25-like | 2 |
| IPR008409 | Pre-mRNA-splicing factor SPF27 | 2 |
| IPR007144 | Small-subunit processome, Utp11 | 2 |
| IPR019412 | Outer membrane protein Iml2/Tetratricopeptide repeat protein 39 | 2 |
| IPR016721 | Bet3 family | 2 |
| IPR026635 | Protein-lysine N-methyltransferase Efm4 | 2 |
| IPR011184 | DNA mismatch repair MutS | 2 |
| IPR013865 | Protein of unknown function DUF1754, eukaryotic | 2 |
| IPR005574 | RNA polymerase II, Rpb4 | 2 |
| IPR018022 | tRNA dimethylallyltransferase | 2 |
| IPR004308 | Glutamate-cysteine ligase catalytic subunit | 2 |
| IPR004539 | Translation elongation factor EF1A, eukaryotic/archaeal | 2 |
| IPR005198 | Glycoside hydrolase, family 76 | 2 |
| IPR002060 | Squalene/phytoene synthase | 2 |
| IPR006654 | Tryptophan synthase, beta chain | 2 |
| IPR006248 | Aconitase, mitochondrial-like | 2 |
| IPR013020 | DNA helicase (DNA repair), Rad3 type | 2 |
| IPR010240 | Cysteine desulfurase IscS | 2 |
| IPR010754 | Optic atrophy 3-like | 2 |
| IPR016342 | AP-1, 2,4 complex subunit beta | 2 |
| IPR015803 | Cysteine-tRNA ligase | 2 |
| IPR031103 | 4-hydroxybenzoate octaprenyltransferase | 2 |
| IPR016727 | ATPase, V0 complex, subunit d | 2 |
| IPR026971 | Condensin subunit 1/Condensin-2 complex subunit D3 | 2 |
| IPR006056 | RidA family | 2 |
| IPR000903 | Myristoyl-CoA:protein N-myristoyltransferase | 2 |
| IPR007317 | Uncharacterised protein family UPF0363 | 2 |
| IPR014186 | S-formylglutathione hydrolase | 2 |
| IPR022879 | V-type ATP synthase regulatory subunit B/beta | 2 |
| IPR023601 | Golgi SNAP receptor complex, subunit 1 | 2 |
| IPR000791 | Acetate transporter GPR1/FUN34/SatP family | 2 |
| IPR012681 | Nucleobase cation symporter-1, NCS1 | 2 |
| IPR006259 | Adenylate kinase subfamily | 2 |
| IPR013261 | Mitochondrial import inner membrane translocase subunit Tim21 | 2 |
| IPR010140 | Histidinol phosphate phosphatase, HisJ | 2 |
| IPR010756 | Hepatocellular carcinoma-associated antigen 59 | 2 |
| IPR019186 | Nucleolar protein 12 | 2 |
| IPR002657 | Bile acid:sodium symporter/arsenical resistance protein Acr3 | 2 |
| IPR002076 | ELO family | 2 |
| IPR009548 | Protein of unknown function DUF1168 | 2 |
| IPR006687 | Small GTPase superfamily, SAR1-type | 2 |
| IPR007246 | GPI transamidase component Gaa1 | 2 |
| IPR025653 | Peroxisome biogenesis factor 1 | 2 |
| IPR007273 | SCAMP | 2 |
| IPR006379 | HAD-superfamily hydrolase, subfamily IIB | 2 |
| IPR008551 | Transport and Golgi organisation protein 2 | 2 |
| IPR019927 | Ribosomal protein L3, bacterial/organelle-type | 2 |
| IPR011206 | Citrate lyase beta subunit-like | 2 |
| IPR024652 | Trichodiene synthase | 2 |
| IPR006275 | Carbamoyl-phosphate synthase, large subunit | 2 |
| IPR005930 | Pyruvate carboxylase | 2 |
| IPR011599 | Prefoldin alpha subunit, archaea-type | 2 |
| IPR008603 | Dynactin subunit 4 | 2 |
| IPR005552 | Scramblase | 2 |
| IPR033517 | DDX54/DBP10 family | 2 |
| IPR029175 | Exocyst complex component EXOC2/Sec5 | 2 |
| IPR004882 | Luc7-related | 2 |
| IPR000422 | 3,4-dihydroxy-2-butanone 4-phosphate synthase, RibB | 2 |
| IPR008854 | TPMT family | 2 |
| IPR017782 | Hydroxyacylglutathione hydrolase | 2 |
| IPR003718 | OsmC/Ohr family | 2 |
| IPR015267 | Protein phosphatase 4 core regulatory subunit R2 | 2 |
| IPR007262 | Vacuolar protein sorting 55 | 2 |
| IPR002975 | Fungal G-protein, alpha subunit | 2 |
| IPR019388 | Fat storage-inducing transmembrane protein | 2 |
| IPR008289 | Pentafunctional AroM protein | 2 |
| IPR007258 | Vps52 | 2 |
| IPR007174 | Las1-like | 2 |
| IPR007231 | Nucleoporin interacting component Nup93/Nic96 | 2 |
| IPR033308 | PGAP5/Cdc1/Ted1 | 2 |
| IPR016965 | Phosphatase PHOSPHO-type | 2 |
| IPR018450 | Romo1/Mgr2 | 2 |
| IPR000116 | High mobility group protein HMGA | 2 |
| IPR002099 | DNA mismatch repair protein family | 2 |
| IPR005304 | Ribosomal biogenesis, methyltransferase, EMG1/NEP1 | 2 |
| IPR002318 | Alanine-tRNA ligase, class IIc | 2 |
| IPR007676 | Ribophorin I | 2 |
| IPR008803 | RHD3/Sey1 | 2 |
| IPR010770 | Ecd family | 2 |
| IPR010440 | Lipopolysaccharide kinase | 2 |
| IPR004434 | Isocitrate dehydrogenase NAD-dependent | 2 |
| IPR001250 | Mannose-6-phosphate isomerase, type I | 2 |
| IPR010816 | Heterokaryon incompatibility Het-C | 2 |
| IPR031101 | RNA polymerase-associated protein Ctr9 | 2 |
| IPR008076 | Cyanate hydratase | 2 |
| IPR021183 | N-terminal acetyltransferase A, auxiliary subunit | 2 |
| IPR027094 | Mitofusin family | 2 |
| IPR002833 | Peptidyl-tRNA hydrolase, PTH2 | 2 |
| IPR027091 | Transmembrane GTPase Fzo/Fzo1 | 2 |
| IPR009448 | UDP-glucose:Glycoprotein Glucosyltransferase | 2 |
| IPR001805 | Adenosine kinase | 2 |
| IPR033162 | Tubulin-folding cofactor D | 2 |
| IPR021151 | GINS complex | 2 |
| IPR005550 | Kinetochore protein Ndc80 | 2 |
| IPR012479 | SAP30-binding protein | 2 |
| IPR030054 | Actin-related protein 6 | 2 |
| IPR004709 | Na+/H+ exchanger | 2 |
| IPR011513 | Non-structural maintenance of chromosomes element 1 | 2 |
| IPR004728 | Translocation protein Sec62 | 2 |
| IPR010164 | Ornithine aminotransferase | 2 |
| IPR008335 | Eukaryotic molybdopterin oxidoreductase | 2 |
| IPR019335 | Conserved oligomeric Golgi complex subunit 7 | 2 |
| IPR007239 | Autophagy-related protein 5 | 2 |
| IPR003440 | Glycosyl transferase, family 48 | 2 |
| IPR004579 | ERCC1/RAD10/SWI10 family | 2 |
| IPR006329 | AMP deaminase | 2 |
| IPR032832 | E3 ubiquitin-protein ligase synoviolin/Hrd1 | 2 |
| IPR027244 | Vacuolar membrane-associated protein Iml1 | 2 |
| IPR006297 | Elongation factor 4 | 2 |
| IPR008676 | MRG | 2 |
| IPR004408 | Biotin--acetyl-CoA-carboxylase ligase | 2 |
| IPR024326 | Ribosomal RNA-processing protein 7 | 2 |
| IPR002994 | Surfeit locus 1/Shy1 | 2 |
| IPR021858 | Fungal transcription factor | 2 |
| IPR001270 | ClpA/B family | 2 |
| IPR015661 | Mitotic spindle checkpoint protein Bub1/Mad3 | 2 |
| IPR003545 | Telomerase reverse transcriptase | 2 |
| IPR007145 | Microtubule-associated protein, MAP65/Ase1/PRC1 | 2 |
| IPR015034 | Protein of unknown function DUF1917 | 2 |
| IPR021150 | Ubiquinol-cytochrome c chaperone/UPF0174 | 2 |
| IPR012459 | Ribosomal RNA-processing protein 15 | 2 |
| IPR004695 | Voltage-dependent anion channel | 2 |
| IPR004511 | Phosphoadenosine phosphosulphate/adenosine 5'-phosphosulphate reductase | 2 |
| IPR015496 | Ubiquilin | 2 |
| IPR004706 | Arsenical-resistance protein Acr3 | 2 |
| IPR017665 | Guanylate kinase | 2 |
| IPR005945 | Proline-specific peptidase | 2 |
| IPR004241 | Autophagy protein Atg8 ubiquitin-like | 2 |
| IPR005193 | Glycoside hydrolase, family 62, arabinosidase | 2 |
| IPR003697 | Maf-like protein | 2 |
| IPR016706 | Cleavage/polyadenylation specificity factor subunit 5 | 2 |
| IPR007175 | RNAse P, Rpr2/Rpp21 subunit | 2 |
| IPR007005 | XAP5 protein | 2 |
| IPR009846 | Splicing factor 3B subunit 5/RDS3 complex subunit 10 | 2 |
| IPR001948 | Peptidase M18 | 2 |
| IPR016300 | Arsenical pump ATPase, ArsA/GET3 | 2 |
| IPR005668 | 2-isopropylmalate synthase | 2 |
| IPR014472 | Choline/ethanolamine phosphotransferase | 2 |
| IPR018539 | SUN domain-containing protein 1 | 2 |
| IPR023582 | Impact family | 2 |
| IPR002842 | ATPase, V1/A1 complex, subunit E | 2 |
| IPR017375 | Peroxisome assembly protein 12 | 2 |
| IPR000544 | Octanoyltransferase | 2 |
| IPR027842 | Protein of unknown function DUF4449 | 2 |
| IPR032974 | Polyprenol kinase family | 2 |
| IPR010516 | Sin3 associated polypeptide p18 | 2 |
| IPR003702 | Acetyl-CoA hydrolase/transferase | 2 |
| IPR005627 | Copper homeostasis protein CutC | 2 |
| IPR022816 | Condensin complex subunit 2/barren | 2 |
| IPR016439 | Ceramide synthase component Lag1/Lac1 | 2 |
| IPR006266 | UMP-CMP kinase | 2 |
| IPR008728 | Elongator complex protein 4 | 2 |
| IPR000597 | Ribosomal protein L3 | 2 |
| IPR011343 | Deoxyribose-phosphate aldolase | 2 |
| IPR022309 | Ribosomal protein S8e/ribosomal biogenesis NSA2 | 2 |
| IPR009600 | GPI transamidase subunit PIG-U | 2 |
| IPR002327 | Cytochrome c, class IA/ IB | 2 |
| IPR000103 | Pyridine nucleotide-disulphide oxidoreductase, class-II | 2 |
| IPR005819 | Histone H5 | 2 |
| IPR022057 | Chitin synthase III catalytic subunit | 2 |
| IPR026008 | Uridine kinase | 2 |
| IPR019382 | Translation initiation factor 3 complex subunit L | 2 |
| IPR016818 | Nitric oxide synthase-interacting | 2 |
| IPR005442 | Glutathione S-transferase, omega-class | 2 |
| IPR006931 | Calcipressin | 2 |
| IPR004636 | Acetylornithine/Succinylornithine transaminase family | 2 |
| IPR012722 | T-complex protein 1, zeta subunit | 2 |
| IPR026768 | Protein FAM72 | 2 |
| IPR018946 | Alkaline phosphatase D-related | 2 |
| IPR000424 | Primosome PriB/single-strand DNA-binding | 2 |
| IPR029685 | Structural maintenance of chromosomes protein 3 | 2 |
| IPR004621 | Eukaryotic-type methylenetetrahydrofolate reductase | 2 |
| IPR005722 | ATPase, F1 complex, beta subunit | 2 |
| IPR012981 | PIH1 family | 2 |
| IPR001580 | Calreticulin/calnexin | 2 |
| IPR016655 | Prefoldin subunit 3 | 2 |
| IPR005037 | Pre-mRNA-splicing factor 38 | 2 |
| IPR001593 | Ribosomal protein S3Ae | 2 |
| IPR002930 | Glycine cleavage system H-protein | 2 |
| IPR002472 | Palmitoyl protein thioesterase | 2 |
| IPR003422 | Cytochrome b-c1 complex, subunit 6 | 2 |
| IPR000851 | Ribosomal protein S5 | 2 |
| IPR005002 | Eukaryotic phosphomannomutase | 2 |
| IPR026893 | Tyrosine/serine-protein phosphatase IphP-type | 2 |
| IPR027238 | RuvB-like | 2 |
| IPR006384 | Pyridoxal phosphate phosphatase-related | 2 |
| IPR004733 | Phosphoribosylformylglycinamidine cyclo-ligase | 2 |
| IPR009447 | GWT1 | 2 |
| IPR031047 | DNA helicase Ino80 | 2 |
| IPR024420 | TRAPP III complex, Trs85 | 2 |
| IPR013922 | Cyclin PHO80-like | 2 |
| IPR006716 | ERG2/sigma1 receptor-like | 2 |
| IPR009445 | Protein of unknown function DUF1077, TMEM85 | 2 |
| IPR010226 | NADH-quinone oxidoreductase, chain I | 2 |
| IPR017072 | Transcription elongation factor Spt6 | 2 |
| IPR010776 | Tat binding protein 1-interacting | 2 |
| IPR010285 | DNA helicase Pif1-like | 2 |
| IPR022495 | Serine/threonine-protein kinase Bud32 | 2 |
| IPR002292 | Ornithine/putrescine carbamoyltransferase | 2 |
| IPR007823 | Ribosomal RNA processing protein 8 | 2 |
| IPR016528 | Vacuolar protein sorting-associated protein 11 | 2 |
| IPR020796 | Origin recognition complex, subunit 5 | 2 |
| IPR005124 | Vacuolar (H+)-ATPase G subunit | 2 |
| IPR029559 | Membrane protein Tms1-like | 2 |
| IPR017046 | Prenylcysteine oxidase | 2 |
| IPR028297 | Wiskott-Aldrich syndrome protein homologue, fungal | 2 |
| IPR026871 | Phospholipid-transporting P-type ATPase | 2 |
| IPR006368 | GDP-mannose 4,6-dehydratase | 2 |
| IPR006994 | Transcription factor 25 | 2 |
| IPR007257 | DNA replication complex GINS protein Psf2 | 2 |
| IPR004547 | Glucosamine-6-phosphate isomerase | 2 |
| IPR001975 | Ribosomal protein L40e | 2 |
| IPR005502 | ADP-ribosylation/Crystallin J1 | 2 |
| IPR008568 | Uncharacterised conserved protein UCP010045, transmembrane eukaryotic | 2 |
| IPR019402 | Frag1/DRAM/Sfk1 | 2 |
| IPR020164 | Cytochrome c oxidase assembly protein COX16 | 2 |
| IPR010107 | Glutamate decarboxylase | 2 |
| IPR004506 | tRNA-specific 2-thiouridylase | 2 |
| IPR027665 | Actin-related protein 9, fungi | 2 |
| IPR007277 | Transmembrane adaptor Erv26 | 2 |
| IPR004468 | CTP synthase | 2 |
| IPR019416 | Nuclear cap-binding protein subunit 3 | 2 |
| IPR002409 | Aflatoxin biosynthesis regulatory protein | 2 |
| IPR026195 | P-selectin glycoprotein ligand 1 | 2 |
| IPR021843 | Protein of unknown function DUF3437 | 2 |
| IPR004806 | UV excision repair protein Rad23 | 2 |
| IPR027534 | Ribosomal protein L12 family | 2 |
| IPR009890 | Etoposide-induced 2.4 | 2 |
| IPR008672 | Spindle assembly checkpoint component Mad1 | 2 |
| IPR000115 | Phosphoribosylglycinamide synthetase | 2 |
| IPR002915 | DeoC/FbaB/ lacD aldolase | 2 |
| IPR009360 | Isy1-like splicing | 2 |
| IPR028325 | Voltage-gated potassium channel | 2 |
| IPR027038 | Ran GTPase-activating protein | 2 |
| IPR005013 | Dolichyl-diphosphooligosaccharide--protein glycosyltransferase 48 kDa subunit | 2 |
| IPR010829 | Cerato-platanin | 2 |
| IPR004372 | Acetate/propionate kinase | 2 |
| IPR028245 | Eisosome component PIL1/LSP1 | 2 |
| IPR016270 | Phospholipase D, phosphatidylserine synthase type | 2 |
| IPR011356 | Peptidase M17, leucine aminopeptidase/peptidase B | 2 |
| IPR004798 | Calcium/proton exchanger CAX | 2 |
| IPR008429 | Cleft lip and palate transmembrane 1 | 2 |
| IPR006984 | rRNA-processing protein Fcf1/Utp23 | 2 |
| IPR004910 | Yippee/Mis18/Cereblon | 2 |
| IPR005755 | Ribosomal protein L13, eukaryotic/archaeal | 2 |
| IPR007881 | UNC-50 | 2 |
| IPR014848 | Reduced growth phenotype protein 1 | 2 |
| IPR007859 | ETF-QO/FixX | 2 |
| IPR010591 | ATP11 | 2 |
| IPR018624 | Translocation protein Sec66 | 2 |
| IPR001921 | Ribosomal protein L7A/L8 | 2 |
| IPR003194 | Transcription initiation factor IIA, gamma subunit | 2 |
| IPR030564 | Myotubularin family | 2 |
| IPR026681 | Nicotinamide riboside kinase | 2 |
| IPR001232 | S-phase kinase-associated protein 1-like | 2 |
| IPR007541 | Uncharacterised protein family, basic secretory protein | 2 |
| IPR007528 | RINT-1/Tip20 | 2 |
| IPR006838 | FAR-17a/AIG1-like protein | 2 |
| IPR008775 | Phytanoyl-CoA dioxygenase | 2 |
| IPR004413 | Aspartyl/glutamyl-tRNA(Asn/Gln) amidotransferase, B subunit | 2 |
| IPR005677 | Fumarate hydratase, class II | 2 |
| IPR010401 | Glycogen debranching enzyme | 2 |
| IPR013283 | ABC transporter ABCE | 2 |
| IPR008831 | Mediator complex, subunit Med31 | 2 |
| IPR010422 | Coiled-coil domain-containing protein 124 | 2 |
| IPR003913 | Tuberin | 2 |
| IPR021851 | Protein of unknown function DUF3455 | 2 |
| IPR013907 | Sds3-like | 2 |
| IPR001518 | Argininosuccinate synthase | 2 |
| IPR019150 | Vesicle transport protein, Use1 | 2 |
| IPR017867 | Protein-tyrosine phosphatase, low molecular weight | 2 |
| IPR001185 | Large-conductance mechanosensitive channel | 2 |
| IPR007504 | H/ACA ribonucleoprotein complex, subunit Gar1/Naf1 | 2 |
| IPR027132 | Structural maintenance of chromosomes protein 6 | 2 |
| IPR016467 | DNA recombination and repair protein, RecA-like | 2 |
| IPR001045 | Spermidine/spermine synthases | 2 |
| IPR009332 | Mediator of RNA polymerase II transcription subunit 22 | 2 |
| IPR014492 | Poly(A) polymerase | 2 |
| IPR017423 | tRNA (adenine(58)-N(1))-methyltransferase non-catalytic subunit TRM6 | 2 |
| IPR008814 | Dolichyl-diphosphooligosaccharide--protein glycosyltransferase subunit Swp1 | 2 |
| IPR030878 | Ribosomal protein L15 | 1 |
| IPR021861 | THO complex, subunit THOC1 | 1 |
| IPR027277 | Nicotinate-nucleotide pyrophosphorylase/Putative pyrophosphorylase ModD | 1 |
| IPR007238 | DNA primase large subunit, eukaryotic/archaeal | 1 |
| IPR016901 | Anaphase-promoting complex subunit 10 | 1 |
| IPR023754 | Heme A synthase, type 2 | 1 |
| IPR028356 | UDP-glucose 6-dehydrogenase, eukaryotic type | 1 |
| IPR013226 | Pal1 cell morphology | 1 |
| IPR016210 | NAD-dependent glutamate dehydrogenase, eukaryotes | 1 |
| IPR000701 | Succinate dehydrogenase/Fumarate reductase, transmembrane subunit | 1 |
| IPR030507 | Bem1/Scd2 | 1 |
| IPR022024 | Protein of unknown function DUF3602 | 1 |
| IPR002659 | Glycosyl transferase, family 31 | 1 |
| IPR004585 | DNA recombination/repair protein Rad52 | 1 |
| IPR012420 | Cbp4 | 1 |
| IPR030616 | Aurora kinase | 1 |
| IPR010923 | tRNA threonylcarbamoyladenosine biosynthesis protein SUA5 | 1 |
| IPR012164 | DNA-directed RNA polymerase subunit/transcription factor S | 1 |
| IPR006003 | Carbohydrate kinase, FGGY-related | 1 |
| IPR006262 | Cytidine deaminase, homotetrameric | 1 |
| IPR002316 | Proline-tRNA ligase, class IIa | 1 |
| IPR027495 | Thiamine thiazole synthase | 1 |
| IPR021137 | Ribosomal protein L35 | 1 |
| IPR012571 | Mitochondrial distribution and morphology protein family 31/32, fungi | 1 |
| IPR016589 | tRNA-splicing endonuclease, SEN2 subunit | 1 |
| IPR008048 | DNA replication licensing factor Mcm | 1 |
| IPR008554 | Glutaredoxin-like | 1 |
| IPR001161 | Helicase XPB/Ssl2 | 1 |
| IPR027546 | Sirtuin, class III | 1 |
| IPR006639 | Presenilin/signal peptide peptidase | 1 |
| IPR000178 | Translation initiation factor aIF-2, bacterial-like | 1 |
| IPR027711 | Rmd5 | 1 |
| IPR011236 | Serine/threonine protein phosphatase 5 | 1 |
| IPR011559 | Initiation factor 2B alpha/beta/delta | 1 |
| IPR005719 | Dihydroorotate dehydrogenase, class 2 | 1 |
| IPR011800 | Phosphoadenosine phosphosulphate reductase CysH | 1 |
| IPR028363 | DNA-directed RNA polymerase, subunit RPB6 | 1 |
| IPR021163 | Ferredoxin-NADP+ reductase, adrenodoxin-type | 1 |
| IPR008381 | ACN9 | 1 |
| IPR019487 | RAM signalling pathway, SOG2 | 1 |
| IPR024712 | Catalase, mono-functional, haem-containing, clade 2 | 1 |
| IPR005579 | Cgr1-like | 1 |
| IPR005944 | Proline iminopeptidase | 1 |
| IPR029702 | DNA polymerase alpha catalytic subunit | 1 |
| IPR012933 | HicA mRNA interferase family | 1 |
| IPR026028 | ATPase, V0 complex, subunit 116kDa, eukaryotic | 1 |
| IPR011553 | Translocation protein Sec62, ascomycota | 1 |
| IPR027111 | Mitochondrial import inner membrane translocase subunit Tim50 | 1 |
| IPR028133 | Dynamitin | 1 |
| IPR023244 | Brefeldin A-sensitivity protein 4 | 1 |
| IPR025700 | L-lysine 6-monooxygenase/L-ornithine 5-monooxygenase | 1 |
| IPR006529 | U2 snRNP auxilliary factor, large subunit, splicing factor | 1 |
| IPR021331 | Protein of unknown function DUF2945 | 1 |
| IPR027093 | EAF family | 1 |
| IPR012776 | Trimethyllysine dioxygenase | 1 |
| IPR013954 | Polynucleotide kinase 3 phosphatase | 1 |
| IPR019193 | Ubiquitin-conjugating enzyme E2-binding protein | 1 |
| IPR019508 | Proteinase inhibitor I48, clitocypin | 1 |
| IPR014812 | Vacuolar protein sorting-associated protein 51 | 1 |
| IPR005676 | Aspartate-semialdehyde dehydrogenase, peptidoglycan lacking | 1 |
| IPR027514 | Methylthioribulose-1-phosphate dehydratase, eukaryotes | 1 |
| IPR012469 | Protein of unknown function DUF1688 | 1 |
| IPR019327 | Protein of unknown function DUF2373 | 1 |
| IPR002582 | Holo-[acyl carrier protein] synthase | 1 |
| IPR004580 | DNA repair protein, Rad18 | 1 |
| IPR030224 | Sla2 family | 1 |
| IPR022694 | 3-hydroxyacyl-CoA dehydrogenase | 1 |
| IPR017959 | Aspartyl/glutamyl-tRNA(Asn/Gln) amidotransferase, subunit B /E | 1 |
| IPR010332 | ATPase terminase subunit, putative | 1 |
| IPR021130 | Phosphoribosyl-ATP pyrophosphohydrolase-like | 1 |
| IPR024928 | E3 ubiquitin-protein ligase, SMURF1 type | 1 |
| IPR012137 | Nitrate reductase NADH dependent | 1 |
| IPR026183 | Taxilin family | 1 |
| IPR029710 | DNA ligase 4 | 1 |
| IPR016897 | S-phase kinase-associated protein 1 | 1 |
| IPR008089 | Nucleotide sugar epimerase | 1 |
| IPR002669 | Urease accessory protein UreD | 1 |
| IPR002402 | Cytochrome P450, E-class, group II | 1 |
| IPR000623 | Shikimate kinase/Threonine synthase-like 1 | 1 |
| IPR005678 | Mitochondrial inner membrane translocase complex, subunit Tim17 | 1 |
| IPR019188 | Small nuclear RNA activating complex (SNAPc), subunit SNAP43 | 1 |
| IPR016688 | Mechanosensitive ion channel MscS-like, plants/fungi | 1 |
| IPR009960 | Fungal fruit body lectin | 1 |
| IPR009598 | Bladder cancer-related BC10 | 1 |
| IPR005501 | Uncharacterised protein family UPF0271, LamB/YcsF | 1 |
| IPR007065 | HPP | 1 |
| IPR010102 | Succinate semialdehyde dehydrogenase | 1 |
| IPR002207 | Plant ascorbate peroxidase | 1 |
| IPR018850 | Mitochondrial escape protein 2 | 1 |
| IPR009617 | Seipin family | 1 |
| IPR019021 | E3 ubiquitin-protein ligase substrate receptor Mms22 | 1 |
| IPR019436 | Steryl acetyl hydrolase 1-like | 1 |
| IPR026010 | Nucleoporin NSP1/NUP62 | 1 |
| IPR017597 | Pyruvate dehydrogenase (acetyl-transferring) E1 component, alpha subunit, subgroup y | 1 |
| IPR017104 | Adaptor protein complex AP-2, alpha subunit | 1 |
| IPR009297 | Protein of unknown function DUF952 | 1 |
| IPR006407 | 1,4-alpha-glucan-branching enzyme, GlgB | 1 |
| IPR013970 | Replication factor A protein 3 | 1 |
| IPR016903 | Nucleolar complex-associated protein 3 | 1 |
| IPR001197 | Ribosomal protein L10e | 1 |
| IPR027097 | Mitotic spindle checkpoint protein Mad2 | 1 |
| IPR016046 | Transcription initiation Spt4-like | 1 |
| IPR003392 | Protein patched/dispatched | 1 |
| IPR033275 | E3 ubiquitin-protein ligase MARCH-like | 1 |
| IPR003855 | Potassium transporter | 1 |
| IPR007240 | Autophagy-related protein 17 | 1 |
| IPR019953 | Organic hydroperoxide resistance protein famiy | 1 |
| IPR024177 | Biotin synthase | 1 |
| IPR004649 | Ribonuclease H2, subunit A | 1 |
| IPR008476 | Protein PBDC1, metazoa/fungi | 1 |
| IPR007307 | Low temperature viability protein | 1 |
| IPR019167 | Topoisomerase II-associated protein PAT1 | 1 |
| IPR025788 | Histone-lysine N-methyltransferase, SET2, fungi | 1 |
| IPR014349 | Rieske iron-sulphur protein | 1 |
| IPR026986 | Kinesin-like protein KIF22 (Kid) | 1 |
| IPR026869 | Putative glutamine amidotransferase type 2 | 1 |
| IPR019038 | DNA polymerase subunit Cdc27 | 1 |
| IPR014033 | Arginase | 1 |
| IPR008828 | TORC2 component Sin1/Avo1 | 1 |
| IPR027109 | SWR1-complex protein 4/DNA methyltransferase 1-associated protein 1 | 1 |
| IPR018822 | Uncharacterised protein family UPF0646 | 1 |
| IPR009305 | Protein of unknown function DUF962 | 1 |
| IPR033682 | Palmitoyltransferase PFA4 | 1 |
| IPR024371 | Acetyl-coenzyme A transporter 1 | 1 |
| IPR019340 | Histone acetyltransferases subunit 3 | 1 |
| IPR011541 | Nickel/cobalt transporter, high-affinity | 1 |
| IPR028608 | Probable cytosolic iron-sulfur protein assembly protein, CIAO1/Cia1 | 1 |
| IPR027545 | Kynurenine 3-monooxygenase | 1 |
| IPR004405 | Translation release factor pelota | 1 |
| IPR027542 | Arsenical pump ATPase, ArsA/GET3, eukaryotic | 1 |
| IPR007420 | Protein of unknown function DUF465 | 1 |
| IPR013950 | Kinetochore Mis14 | 1 |
| IPR027104 | U4/U6 small nuclear ribonucleoprotein Prp3 | 1 |
| IPR012098 | Inorganic phosphate transporter Pho88, fungi | 1 |
| IPR024461 | Coiled-coil domain-containing protein 90-like | 1 |
| IPR006095 | Glutamate/phenylalanine/leucine/valine dehydrogenase | 1 |
| IPR022238 | Uncharacterised protein family, methyltransferase, Williams-Beuren syndrome | 1 |
| IPR005713 | Ribosomal protein S19A/S15e | 1 |
| IPR023434 | Argininosuccinate synthase, type 1 subfamily | 1 |
| IPR005938 | AAA ATPase, CDC48 family | 1 |
| IPR002882 | LPPG:FO 2-phospho-L-lactate transferase CofD/UPF0052 | 1 |
| IPR021867 | 25S rRNA (adenine(2142)-N(1))-methyltransferase, Bmt2 | 1 |
| IPR018307 | AVL9/DENND6 domain | 1 |
| IPR004911 | Gamma interferon inducible lysosomal thiol reductase GILT | 1 |
| IPR021827 | Nucleoporin Nup186/Nup192/Nup205 | 1 |
| IPR004591 | Replication factor A protein 1 | 1 |
| IPR017380 | Histone acetyltransferase type B, catalytic subunit | 1 |
| IPR019623 | Protein Rot1 | 1 |
| IPR016207 | Ketol-acid reductoisomerase, fungi | 1 |
| IPR010233 | Ubiquinone biosynthesis O-methyltransferase | 1 |
| IPR027084 | Dual specificity protein kinase TTK | 1 |
| IPR028591 | DIS3-like exonuclease 2 | 1 |
| IPR022955 | GMP synthase | 1 |
| IPR012734 | Dihydroxyacetone kinase | 1 |
| IPR000533 | Tropomyosin | 1 |
| IPR002755 | DNA primase, small subunit | 1 |
| IPR012259 | Dihydrofolate reductase | 1 |
| IPR022953 | ATP-dependent 6-phosphofructokinase | 1 |
| IPR028110 | Transmembrane protein 254 | 1 |
| IPR000212 | DNA helicase, UvrD/REP type | 1 |
| IPR019519 | Elongator complex protein 5 | 1 |
| IPR023042 | Peptidase M17, leucine aminopeptidase | 1 |
| IPR004277 | Phosphatidyl serine synthase | 1 |
| IPR032641 | Exonuclease 1 | 1 |
| IPR011339 | ISC system FeS cluster assembly, IscU scaffold | 1 |
| IPR027328 | Microtubule-associated protein RP/EB | 1 |
| IPR021036 | Ribosomal protein S35, mitochondrial | 1 |
| IPR005764 | Adenine phosphoribosyl transferase | 1 |
| IPR011205 | Uncharacterised conserved protein UCP015417, vWA | 1 |
| IPR000509 | Ribosomal protein L36e | 1 |
| IPR012386 | 2',3'-cyclic-nucleotide 3'-phosphodiesterase | 1 |
| IPR011671 | tRNA (uracil-O(2)-)-methyltransferase | 1 |
| IPR027131 | Structural maintenance of chromosomes protein 5 | 1 |
| IPR014362 | Glutamate dehydrogenase | 1 |
| IPR006408 | P-type ATPase, subfamily IIB | 1 |
| IPR019711 | ATPase, F0 complex, subunit H | 1 |
| IPR013241 | RNase P, subunit Pop3 | 1 |
| IPR011904 | Acetate-CoA ligase | 1 |
| IPR002993 | Ornithine decarboxylase antizyme | 1 |
| IPR004895 | Prenylated rab acceptor PRA1 | 1 |
| IPR021475 | Protein of unknown function DUF3128 | 1 |
| IPR014436 | Extradiol aromatic ring-opening dioxygenase, DODA type | 1 |
| IPR013889 | Karyogamy protein, KAR9 | 1 |
| IPR013776 | Alpha-amylase, thermostable | 1 |
| IPR012480 | Heparinase II/III-like | 1 |
| IPR017107 | Adaptor protein complex AP-1, gamma subunit | 1 |
| IPR021836 | Protein of unknown function DUF3429 | 1 |
| IPR017340 | U1 small nuclear ribonucleoprotein C | 1 |
| IPR009675 | TPX2 | 1 |
| IPR030841 | Endonuclease III-like protein 1 | 1 |
| IPR000706 | N-acetyl-gamma-glutamyl-phosphate reductase, type 1 | 1 |
| IPR033510 | PLAA/Doa1/Lub1 | 1 |
| IPR012462 | Peptidase C78, ubiquitin fold modifier-specific peptidase 1/ 2 | 1 |
| IPR026113 | Methyltransferase-like | 1 |
| IPR016651 | Leucine carboxyl methyltransferase 1, LCMT1 | 1 |
| IPR033052 | Cmr1/WDR76 | 1 |
| IPR024679 | Pre-rRNA-processing protein IPI1/Testis-expressed sequence 10 protein | 1 |
| IPR006823 | Neutral/alkaline nonlysosomal ceramidase | 1 |
| IPR004713 | Calcium/proton exchanger | 1 |
| IPR027057 | CAAX prenyl protease 1 | 1 |
| IPR006287 | Protein deglycase DJ-1 | 1 |
| IPR013641 | Protein KTI12/L-seryl-tRNA(Sec) kinase | 1 |
| IPR026051 | Chitobiosyldiphosphodolichol beta-mannosyltransferase ALG1-like | 1 |
| IPR019183 | N-acetyltransferase B complex, non-catalytic subunit | 1 |
| IPR013952 | Protein of unknown function DUF1776, fungi | 1 |
| IPR019324 | M-phase phosphoprotein 6 | 1 |
| IPR000764 | Uridine kinase-like | 1 |
| IPR028884 | tRNA (guanine-N(7)-)-methyltransferase non-catalytic subunit, Trm82 | 1 |
| IPR007218 | DNA polymerase delta, subunit 4 | 1 |
| IPR019342 | NADH:ubiquinone oxidoreductase, iron-sulphur subunit 5 | 1 |
| IPR025066 | Protein of unknown function DUF4078 | 1 |
| IPR011537 | NADH ubiquinone oxidoreductase, F subunit | 1 |
| IPR019404 | Mediator complex, subunit Med11 | 1 |
| IPR007852 | Cdc73/Parafibromin | 1 |
| IPR015381 | XLF family | 1 |
| IPR021419 | Mediator complex, subunit Med25, von Willebrand factor type A | 1 |
| IPR002053 | Glycoside hydrolase, family 25 | 1 |
| IPR006349 | 2-phosphoglycolate phosphatase, eukaryotic | 1 |
| IPR017153 | Cytosolic nonspecific dipeptidase/DUG1 | 1 |
| IPR001701 | Glycoside hydrolase family 9 | 1 |
| IPR012705 | 2-methylcitrate dehydratase PrpD | 1 |
| IPR012535 | Cell division protein Cdc14 | 1 |
| IPR003038 | DAD/Ost2 | 1 |
| IPR015418 | Chromatin modification-related protein Eaf6 | 1 |
| IPR001209 | Ribosomal protein S14 | 1 |
| IPR030108 | Actin patches distal protein 1 | 1 |
| IPR005723 | ATPase, V1 complex, subunit B | 1 |
| IPR027539 | Mitochondrial distribution and morphology protein 10 | 1 |
| IPR015761 | Lipoamide Acyltransferase | 1 |
| IPR021369 | Protein of unknown function DUF2985 | 1 |
| IPR006328 | Haloacid dehalogenase, type II | 1 |
| IPR016690 | tRNA-splicing endonuclease, SEN34 subunit | 1 |
| IPR006411 | Fructose-bisphosphate aldolase, class II, yeast/E. coli subtype | 1 |
| IPR006322 | Glutathione reductase, eukaryote/bacterial | 1 |
| IPR019368 | Ribosomal protein S23/S29, mitochondrial | 1 |
| IPR000715 | Glycosyl transferase, family 4 | 1 |
| IPR020373 | Ribosomal protein S36, mitochondrial | 1 |
| IPR009668 | RNA polymerase I associated factor, A49-like | 1 |
| IPR010122 | Hydroxymethylglutaryl-CoA synthase, eukaryotic | 1 |
| IPR024931 | Importin subunit alpha | 1 |
| IPR008504 | ER membrane protein complex subunit 6 | 1 |
| IPR007247 | Ureidoglycolate lyase | 1 |
| IPR013483 | Molybdenum cofactor biosynthesis protein A | 1 |
| IPR018800 | Proline-rich protein PRCC | 1 |
| IPR001706 | Ribosomal protein L35, non-mitochondrial | 1 |
| IPR010684 | RNA polymerase II transcription factor SIII, subunit A | 1 |
| IPR002922 | Thiazole biosynthetic enzyme Thi4 family | 1 |
| IPR004545 | PA2G4 family | 1 |
| IPR003333 | Mycolic acid cyclopropane synthase | 1 |
| IPR026846 | E3 SUMO-protein ligase Nse2 (Mms21) | 1 |
| IPR000689 | Ubiquinone biosynthesis monooxygenase COQ6 | 1 |
| IPR007244 | NatC N(alpha)-terminal acetyltransferase, Mak10 subunit | 1 |
| IPR005624 | Haem-degrading | 1 |
| IPR028600 | Cytosolic Fe-S cluster assembly factor NUBP2/Cfd1, eukaryotes | 1 |
| IPR009580 | GPI biosynthesis protein Pig-F | 1 |
| IPR010613 | Pescadillo | 1 |
| IPR016340 | Ribosomal protein L31, mitochondrial | 1 |
| IPR026812 | Actin cytoskeleton-regulatory complex protein Pan1 | 1 |
| IPR019400 | Peptidase C65, otubain | 1 |
| IPR012134 | Glutamate-5-semialdehyde dehydrogenase | 1 |
| IPR002433 | Ornithine decarboxylase | 1 |
| IPR001352 | Ribonuclease HII/HIII | 1 |
| IPR012875 | Protein of unknown function DUF1674 | 1 |
| IPR014189 | Quinone oxidoreductase PIG3 | 1 |
| IPR016964 | Transmembrane protein 97 | 1 |
| IPR030225 | Sterol regulatory element-binding protein cleavage-activating protein | 1 |
| IPR021346 | Translation machinery-associated protein 16 | 1 |
| IPR003480 | Transferase | 1 |
| IPR005511 | Senescence marker protein-30 (SMP-30) | 1 |
| IPR020621 | ATP phosphoribosyltransferase HisG, long form | 1 |
| IPR028419 | Adenylyl cyclase-associated protein CAP, fungal type | 1 |
| IPR022036 | Protein of unknown function DUF3605 | 1 |
| IPR022941 | Signal recognition particle, SRP54 subunit | 1 |
| IPR004737 | Nitrate transporter | 1 |
| IPR012717 | T-complex protein 1, delta subunit | 1 |
| IPR005792 | Protein disulphide isomerase | 1 |
| IPR000039 | Ribosomal protein L18e | 1 |
| IPR019133 | Mitochondrial inner membrane protein Mitofilin | 1 |
| IPR005703 | Ribosomal protein S3, eukaryotic/archaeal | 1 |
| IPR012198 | cAMP-dependent protein kinase regulatory subunit | 1 |
| IPR001546 | GPCR fungal pheromone A receptor | 1 |
| IPR027159 | Nuclear cap-binding protein subunit 1 | 1 |
| IPR013898 | Protein of unknown function DUF1770, fungi | 1 |
| IPR013965 | DASH complex subunit Dad3 | 1 |
| IPR010977 | Aromatic-L-amino-acid decarboxylase | 1 |
| IPR011548 | 3-hydroxyisobutyrate dehydrogenase | 1 |
| IPR015517 | Deoxycytidylate deaminase-related | 1 |
| IPR008509 | Molybdate-anion transporter | 1 |
| IPR007306 | tRNA A64-2'-O-ribosylphosphate transferase | 1 |
| IPR010318 | Protein of unknown function DUF917 | 1 |
| IPR018075 | Ubiquitin-activating enzyme E1 | 1 |
| IPR013640 | VPS4-associated protein 1 | 1 |
| IPR009917 | Steroid receptor RNA activator-protein/coat protein complex II, Sec31 | 1 |
| IPR005913 | dTDP-4-dehydrorhamnose reductase family | 1 |
| IPR021771 | Triacylglycerol lipase | 1 |
| IPR005578 | Yif1 family | 1 |
| IPR030664 | Succinate dehydrogenase/fumarate reductase, alpha/adenylylsulphate reductase subunit | 1 |
| IPR015637 | G/U mismatch-specific DNA glycosylase | 1 |
| IPR008012 | Proteasome maturation factor Ump1 | 1 |
| IPR019151 | Proteasome assembly chaperone 2 | 1 |
| IPR019190 | Exonuclease V | 1 |
| IPR007150 | Checkpoint protein Hus1/Mec3 | 1 |
| IPR011241 | NAGK-NAGSA, bifunctional | 1 |
| IPR003563 | 7,8-dihydro-8-oxoguanine triphosphatase | 1 |
| IPR017276 | Synthesis of cytochrome c oxidase, Sco1/Sco2 | 1 |
| IPR013635 | Protein Ice2 | 1 |
| IPR011530 | Ribosomal RNA adenine dimethylase | 1 |
| IPR007673 | Condensin subunit 1 | 1 |
| IPR026683 | Serine/threonine-protein kinase TOR | 1 |
| IPR027312 | Sda1 | 1 |
| IPR007364 | RNA methyltransferase TK0422/Sfm1 | 1 |
| IPR027652 | Pre-mRNA-processing-splicing factor 8 | 1 |
| IPR001057 | Glutamate/acetylglutamate kinase | 1 |
| IPR013897 | Protein of unknown function DUF1769 | 1 |
| IPR002113 | Adenine nucleotide translocator 1 | 1 |
| IPR008839 | Mitochondrial distribution and morphology family 33, fungi | 1 |
| IPR024088 | Tyrosine-tRNA ligase, bacterial-type | 1 |
| IPR019145 | Mediator complex, subunit Med10 | 1 |
| IPR018859 | BAR domain-containing family | 1 |
| IPR012066 | Uroporphyrin-III C-methyltransferase, fungi | 1 |
| IPR012878 | Beta-L-arabinofuranosidase, GH127 | 1 |
| IPR001636 | Phosphoribosylaminoimidazole-succinocarboxamide synthase | 1 |
| IPR013924 | Ribonuclease H2, subunit C | 1 |
| IPR029909 | Actin-related protein 1 family | 1 |
| IPR024158 | Mitochondrial import protein TIM15 | 1 |
| IPR012258 | Acyl-CoA oxidase | 1 |
| IPR028581 | Deoxyribose-phosphate aldolase type I | 1 |
| IPR018077 | Glycoside hydrolase, family 25 subgroup | 1 |
| IPR006059 | Solute-binding family 1 | 1 |
| IPR019985 | Ribosomal protein L23 | 1 |
| IPR011488 | Translation initiation factor 2, alpha subunit | 1 |
| IPR024512 | Small subunit of serine palmitoyltransferase-like | 1 |
| IPR009401 | Mediator complex, subunit Med13 | 1 |
| IPR005745 | Ribosomal protein L14P, bacterial-type | 1 |
| IPR024134 | Superoxide dismutase (Cu/Zn) / superoxide dismutase copper chaperone | 1 |
| IPR024929 | Nucleolar GTP-binding protein 2 | 1 |
| IPR026255 | NAD(P) transhydrogenase, alpha subunit | 1 |
| IPR027536 | Mitochondrial distribution and morphology protein 34 | 1 |
| IPR030843 | PAB-dependent poly(A)-specific ribonuclease subunit PAN2 | 1 |
| IPR009262 | Solute carrier family 35 member SLC35F1/F2/F6 | 1 |
| IPR005931 | Delta-1-pyrroline-5-carboxylate dehydrogenase | 1 |
| IPR000206 | Ribosomal protein L7/L12 | 1 |
| IPR021842 | Protein of unknown function DUF3435 | 1 |
| IPR006515 | Polyadenylate binding protein, human types 1, 2, 3, 4 | 1 |
| IPR013252 | Kinetochore-Ndc80 subunit Spc24 | 1 |
| IPR004583 | DNA repair protein Rad4 | 1 |
| IPR008242 | Bifunctional P-protein, chorismate mutase/prephenate dehydratase | 1 |
| IPR006886 | DNA-directed RNA polymerase III subunit Rpc5 | 1 |
| IPR026257 | FK506-binding protein | 1 |
| IPR007402 | Protein of unknown function DUF455 | 1 |
| IPR009374 | Eukaryotic translation initiation factor 3 subunit K | 1 |
| IPR021102 | Peptide-N4-(N-acetyl-beta-glucosaminyl)asparagine amidase A | 1 |
| IPR012404 | Nucleotide-sugar transporter-related | 1 |
| IPR012716 | T-complex protein 1, beta subunit | 1 |
| IPR027317 | PGAP2-interacting protein | 1 |
| IPR016819 | Ribonuclease P/MRP protein subunit Pop5 | 1 |
| IPR026505 | Solute carrier family 35 member F3/F4 | 1 |
| IPR013921 | Mediator complex, subunit Med20 | 1 |
| IPR005290 | Ribosomal protein S15, bacterial-type | 1 |
| IPR031100 | LOG family | 1 |
| IPR007400 | PrpF protein | 1 |
| IPR018828 | Protein of unknown function DUF2034 | 1 |
| IPR000292 | Formate/nitrite transporter | 1 |
| IPR002344 | Lupus La protein | 1 |
| IPR014381 | DNA-directed RNA polymerase RPB5 subunit, eukaryote/virus | 1 |
| IPR028386 | Centromere protein C/Mif2/cnp3 | 1 |
| IPR004769 | Adenylosuccinate lyase | 1 |
| IPR003096 | Smooth muscle protein/calponin | 1 |
| IPR018811 | MIOREX complex component 11 | 1 |
| IPR012471 | Protein of unknown function DUF1690 | 1 |
| IPR004530 | Phenylalanyl-tRNA synthetase, class IIc, mitochondrial | 1 |
| IPR015157 | Translation machinery associated TMA7 | 1 |
| IPR027100 | Mitochondrial import inner membrane translocase subunit Tim10 | 1 |
| IPR004557 | Eukaryotic/archaeal PrmC-related | 1 |
| IPR000969 | Structure-specific recognition protein | 1 |
| IPR030294 | Palmitoyl-protein thioesterase 1 | 1 |
| IPR021848 | Protein of unknown function DUF3445 | 1 |
| IPR017789 | Frataxin | 1 |
| IPR016005 | Phosphomevalonate kinase Erg8 | 1 |
| IPR006424 | Glyceraldehyde-3-phosphate dehydrogenase, type I | 1 |
| IPR016391 | Coatomer alpha subunit | 1 |
| IPR027281 | Saccharopine dehydrogenase [NAD(+), L-lysine-forming] | 1 |
| IPR004738 | Phosphate permease | 1 |
| IPR002794 | Protein of unknown function DUF92, TMEM19 | 1 |
| IPR021627 | Mediator complex, subunit Med27 | 1 |
| IPR013262 | Outer membrane protein, MIM1/TOM13, mitochondrial | 1 |
| IPR013945 | V-type ATPase assembly factor Pkr1 | 1 |
| IPR012715 | T-complex protein 1, alpha subunit | 1 |
| IPR011344 | Single-stranded DNA-binding protein | 1 |
| IPR013940 | Meiosis specific protein Spo22/ZIP4/TEX11 | 1 |
| IPR023468 | Riboflavin kinase | 1 |
| IPR026610 | 3'-RNA ribose 2'-O-methyltransferase, Hen1 | 1 |
| IPR002995 | Surfeit locus 4 | 1 |
| IPR013947 | Mediator complex, subunit Med14 | 1 |
| IPR005549 | Kinetochore protein Nuf2 | 1 |
| IPR022697 | Homoserine dehydrogenase lacking ACT domain | 1 |
| IPR030177 | Vacuolar calcium ion transporter, fungi | 1 |
| IPR033338 | Spc105/Spc7 | 1 |
| IPR010492 | GINS complex, subunit Psf3 | 1 |
| IPR013538 | Activator of Hsp90 ATPase homologue 1-like | 1 |
| IPR007515 | Mss4 | 1 |
| IPR008555 | Suppressor of IKBKE 1 | 1 |
| IPR005680 | Ribosomal protein S23, eukaryotic/archaeal | 1 |
| IPR027157 | Nuclear cap-binding protein subunit 2 | 1 |
| IPR006258 | Dihydrolipoamide dehydrogenase | 1 |
| IPR005606 | Sec20 | 1 |
| IPR019434 | Uncharacterised protein family UPF0642 | 1 |
| IPR022450 | tRNA N6-adenosine threonylcarbamoyltransferase, TsaD | 1 |
| IPR012394 | Aldehyde dehydrogenase NAD(P)-dependent | 1 |
| IPR008501 | THO complex subunit 7/Mft1 | 1 |
| IPR002672 | Ribosomal protein L28e | 1 |
| IPR012866 | Protein of unknown function DUF1644 | 1 |
| IPR010061 | Methylmalonate-semialdehyde dehydrogenase | 1 |
| IPR005651 | Uncharacterised protein family UPF0434/Trm112 | 1 |
| IPR013870 | Ribosomal protein L37, mitochondrial | 1 |
| IPR029146 | CST complex subunit Ten1, animal and plant type | 1 |
| IPR007070 | GPI ethanolamine phosphate transferase 1 | 1 |
| IPR013176 | Protein of unknown function DUF1712, fungi | 1 |
| IPR018482 | Zinc finger, C4H2-type | 1 |
| IPR008181 | Deoxyuridine triphosphate nucleotidohydrolase | 1 |
| IPR030185 | Malic acid transport protein | 1 |
| IPR007216 | Rcd1/Caf40 | 1 |
| IPR011940 | Meiotic recombinase Dmc1 | 1 |
| IPR016966 | Thiamin pyrophosphokinase, eukaryotic | 1 |
| IPR032704 | Protein Cms1 | 1 |
| IPR013255 | Chromosome segregation protein Spc25 | 1 |
| IPR013949 | U3 small nucleolar RNA-associated protein 6 | 1 |
| IPR006746 | 26S proteasome non-ATPase regulatory subunit Rpn12 | 1 |
| IPR009991 | Dynactin subunit p22 | 1 |
| IPR027040 | Proteasome subunit Rpn10 | 1 |
| IPR026270 | Signal recognition particle, SRP72 subunit | 1 |
| IPR014388 | 3-oxoacid CoA-transferase | 1 |
| IPR007715 | Ubiquinone biosynthesis protein Coq4 | 1 |
| IPR006966 | Peroxin-3 | 1 |
| IPR021110 | DNA replication/checkpoint protein | 1 |
| IPR005352 | Erg28 | 1 |
| IPR033661 | Phosphatidylserine decarboxylase, eukaryotic type 1 | 1 |
| IPR033494 | NUDE family | 1 |
| IPR031057 | DOCK1 homologue | 1 |
| IPR030667 | NEDD8-activating enzyme E1 regulatory subunit APP-BP1 | 1 |
| IPR019165 | Peptidase M76, ATP23 | 1 |
| IPR013868 | Tethering factor for nuclear proteasome Cut8/Sts1 | 1 |
| IPR017364 | Gem-associated protein 2 | 1 |
| IPR009582 | Signal peptidase complex subunit 2 | 1 |
| IPR029636 | Protein Csf1 | 1 |
| IPR023270 | tRNA (C5-cytosine) methyltransferase, NCL1 | 1 |
| IPR029525 | INO80 complex, subunit Ies6 | 1 |
| IPR028211 | Pre-mRNA-splicing factor Ntr2 | 1 |
| IPR013745 | TORC2 component Bit61/PRR5 | 1 |
| IPR022158 | Inositol phosphatase | 1 |
| IPR025792 | tRNA (guanine(37)-N(1))-methyltransferase, eukaryotic | 1 |
| IPR024546 | Ribosome biogenesis protein RLP24 | 1 |
| IPR005478 | Transketolase, bacterial-like | 1 |
| IPR025638 | Protein of unknown function DUF4336 | 1 |
| IPR006205 | Mevalonate kinase | 1 |
| IPR006255 | Dihydrolipoamide succinyltransferase | 1 |
| IPR028673 | Syntaxin-16 | 1 |
| IPR032862 | Alpha-ketoglutarate-dependent dioxygenase alkB homologue 6 | 1 |
| IPR006325 | Signal recognition particle, SRP54 subunit, eukaryotic | 1 |
| IPR002218 | tRNA uridine 5-carboxymethylaminomethyl modification enzyme MnmG-related | 1 |
| IPR026873 | Geranylgeranyl transferase type-2 subunit beta | 1 |
| IPR022893 | Shikimate dehydrogenase family | 1 |
| IPR025784 | Nicotinamide N-methyltransferase, putative | 1 |
| IPR005823 | Ribosomal protein L13, bacterial-type | 1 |
| IPR013959 | DASH complex subunit Dad4 | 1 |
| IPR028896 | Aminomethyltransferase/Dimethylsulfonioproprionate demethylase DmdA | 1 |
| IPR032847 | Pre-mRNA-processing factor 17 | 1 |
| IPR005683 | Mitochondrial import receptor subunit Tom22 | 1 |
| IPR008217 | Ccc1 family | 1 |
| IPR012220 | Glutamate synthase, eukaryotic | 1 |
| IPR001348 | ATP phosphoribosyltransferase HisG | 1 |
| IPR008000 | Rhamnose/fucose mutarotase | 1 |
| IPR013960 | DASH complex subunit Duo1 | 1 |
| IPR019148 | Nuclear protein DGCR14 | 1 |
| IPR007019 | Surfeit locus 6 | 1 |
| IPR027081 | CyclinH/Ccl1 | 1 |
| IPR001055 | Adrenodoxin | 1 |
| IPR016443 | RNA 3'-terminal phosphate cyclase type 2 | 1 |
| IPR016657 | Phosphoacetylglucosamine mutase | 1 |
| IPR000374 | Phosphatidate cytidylyltransferase | 1 |
| IPR005725 | ATPase, V1 complex, subunit A | 1 |
| IPR012476 | GLE1-like | 1 |
| IPR023617 | Tyrosine-tRNA ligase, archaeal/eukaryotic-type | 1 |
| IPR010775 | Protein of unknown function DUF1365 | 1 |
| IPR028012 | Protein of unknown function DUF4451 | 1 |
| IPR015324 | Ribosomal protein Rsm22, bacterial-type | 1 |
| IPR027500 | 40S ribosomal protein S1/3, eukaryotes | 1 |
| IPR006735 | Replication termination factor 2 | 1 |
| IPR013048 | Meiotic recombination, Spo11 | 1 |
| IPR028217 | Ribosome-assembly protein 3 | 1 |
| IPR003386 | Lecithin:cholesterol/phospholipid:diacylglycerol acyltransferase | 1 |
| IPR026705 | Hid-1/Ecm30 | 1 |
| IPR006015 | Universal stress protein A | 1 |
| IPR027668 | Actin-related protein 8/Plant actin-related protein 9 | 1 |
| IPR028846 | Recoverin family | 1 |
| IPR007583 | GRASP55/65 | 1 |
| IPR014314 | Succinate dehydrogenase, cytochrome b556 subunit | 1 |
| IPR031102 | RNA polymerase-associated protein Rtf1 | 1 |
| IPR007736 | Caleosin-related | 1 |
| IPR030445 | Histone H3-K79 methyltransferase | 1 |
| IPR019185 | Integral membrane protein SYS1-related | 1 |
| IPR007849 | ATPase assembly factor ATP10 | 1 |
| IPR022278 | Phosphoserine aminotransferase | 1 |
| IPR013875 | Mitochondrial import protein Pam17 | 1 |
| IPR021013 | ATPase, vacuolar ER assembly factor, Vma12 | 1 |
| IPR006518 | Trypanosome RHS | 1 |
| IPR029015 | D-3-phosphoglycerate dehydrogenase, type2 | 1 |
| IPR029887 | ORM protein, fungi | 1 |
| IPR016580 | Cell cycle checkpoint, Hus1 | 1 |
| IPR016295 | Proteasome endopeptidase complex, beta subunit | 1 |
| IPR013289 | CBFA2T family | 1 |
| IPR004773 | Potassium/sodium transporter Trk/HKT | 1 |
| IPR006806 | ETC complex I subunit | 1 |
| IPR018819 | Protein of unknown function DUF2418 | 1 |
| IPR001907 | ATP-dependent Clp protease proteolytic subunit | 1 |
| IPR028614 | GDP-L-fucose synthase/GDP-L-colitose synthase | 1 |
| IPR026874 | Glucosidase 2 subunit beta | 1 |
| IPR024645 | Mitochondrial export protein Som1 | 1 |
| IPR031121 | KH domain containing protein | 1 |
| IPR003285 | Eukaryotic peptide chain release factor GTP-binding subunit | 1 |
| IPR027229 | E3 SUMO protein ligase | 1 |
| IPR018553 | Protein of unknown function DUF2009 | 1 |
| IPR033179 | Phosphatidylserine decarboxylase, prokaryotic type 2 | 1 |
| IPR023811 | Conserved hypothetical protein CHP04076 | 1 |
| IPR027532 | Mitochondrial distribution and morphology protein 12 | 1 |
| IPR023031 | Orotate phosphoribosyltransferase | 1 |
| IPR017359 | Uncharacterised conserved protein UCP038021, RWD | 1 |
| IPR026730 | Mitochondrial inner membrane protease subunit 1 | 1 |
| IPR001911 | Ribosomal protein S21 | 1 |
| IPR019136 | Transcription factor IIIC, subunit 5 | 1 |
| IPR005998 | Ribosomal protein L7, eukaryotic | 1 |
| IPR014183 | Alcohol dehydrogenase class III/S-(hydroxymethyl)glutathione dehydrogenase | 1 |
| IPR007681 | Ran-interacting Mog1 protein | 1 |
| IPR031924 | Glycosyl hydrolase family 115 | 1 |
| IPR017065 | NFU1 iron-sulfur cluster scaffold homologue | 1 |
| IPR028268 | Pianissimo family | 1 |
| IPR028885 | Adenylyltransferase and sulfurtransferase MOCS3/Uba4 | 1 |
| IPR033322 | DNA damage-inducible protein 1 | 1 |
| IPR024711 | Catalase, mono-functional, haem-containing, clades 1 and 3 | 1 |
| IPR033107 | Eukaryotic translation initiation factor 4B | 1 |
| IPR006239 | 3(2),5 -bisphosphate nucleotidase HAL2 | 1 |
| IPR024882 | Nucleoporin p58/p45 | 1 |
| IPR006046 | Alpha amylase | 1 |
| IPR014445 | Glutamine-dependent NAD(+) synthetase | 1 |
| IPR031776 | Splicing factor 3A subunit 3 | 1 |
| IPR007130 | Diacylglycerol acyltransferase | 1 |
| IPR011281 | Succinate dehydrogenase, flavoprotein subunit | 1 |
| IPR030228 | GPN-loop GTPase 3 | 1 |
| IPR006282 | Thiamin pyrophosphokinase | 1 |
| IPR003846 | Uncharacterised protein family UPF0061 | 1 |
| IPR000876 | Ribosomal protein S4e | 1 |
| IPR006905 | Tryptophan halogenase | 1 |
| IPR005269 | Cytokinin riboside 5'-monophosphate phosphoribohydrolase LOG | 1 |
| IPR018786 | Protein of unknown function DUF2343 | 1 |
| IPR006760 | Endosulphine | 1 |
| IPR008047 | Mini-chromosome maintenance complex protein 4 | 1 |
| IPR003692 | Hydantoinase B/oxoprolinase | 1 |
| IPR015505 | Coronin | 1 |
| IPR019313 | Mediator complex, subunit Med17 | 1 |
| IPR005787 | Threonine dehydratase, biosynthetic | 1 |
| IPR017904 | ADF/Cofilin/Destrin | 1 |
| IPR018082 | AmbAllergen | 1 |
| IPR000577 | Carbohydrate kinase, FGGY | 1 |
| IPR006426 | Asparagine synthase, glutamine-hydrolyzing | 1 |
| IPR026532 | Ribosome biogenesis protein BRX1 | 1 |
| IPR005373 | Uncharacterised protein family UPF0183 | 1 |
| IPR023026 | Tryptophan synthase beta chain/beta chain-like | 1 |
| IPR029472 | Gag-polypeptide of LTR copia-type | 1 |
| IPR017510 | Release factor H-coupled RctB family protein | 1 |
| IPR017593 | Allantoinase | 1 |
| IPR001925 | Porin, eukaryotic type | 1 |
| IPR018017 | Nucleoside phosphorylase | 1 |
| IPR018618 | Vacuolar import/degradation protein Vid24 | 1 |
| IPR005344 | Per33/Pom33 family | 1 |
| IPR004607 | Phosphoribosylglycinamide formyltransferase | 1 |
| IPR001533 | Transcriptional coactivator/pterin dehydratase | 1 |
| IPR016453 | Coatomer beta' subunit (COPB2) | 1 |
| IPR028458 | Twinfilin | 1 |
| IPR027786 | Nse4/EID family | 1 |
| IPR006406 | Nicotinate phosphoribosyltransferase | 1 |
| IPR002030 | Mitochondrial brown fat uncoupling protein | 1 |
| IPR006183 | 6-phosphogluconate dehydrogenase | 1 |
| IPR031355 | Protein of unknown function DUF5102 | 1 |
| IPR019906 | Ribosomal protein L6, bacterial-type | 1 |
| IPR019369 | Protein-lysine N-methyltransferase Efm5 | 1 |
| IPR019189 | Ribosomal protein L27/L41, mitochondrial | 1 |
| IPR011425 | Mediator of RNA polymerase II transcription subunit 9 | 1 |
| IPR027193 | Nucleolar complex protein 4 | 1 |
| IPR016563 | Nuclear protein localization protein 4 | 1 |
| IPR023603 | Threonine aldolase | 1 |
| IPR005873 | Density-regulated protein DRP1 | 1 |
| IPR019163 | THO complex, subunit 5 | 1 |
| IPR018814 | Maintenance of telomere capping protein 1, Mtc1 | 1 |
| IPR030142 | Putative calcium-activated chloride channel protein, ciliates | 1 |
| IPR018607 | Chromosome transmission fidelity protein 8 | 1 |
| IPR013219 | Ribosomal protein S27/S33, mitochondrial | 1 |
| IPR013169 | mRNA splicing factor, Cwf18 | 1 |
| IPR001308 | Electron transfer flavoprotein alpha subunit/FixB | 1 |
| IPR019999 | Anthranilate synthase component I-like | 1 |
| IPR018870 | Tti2 family | 1 |
| IPR000801 | Putative esterase | 1 |
| IPR027777 | Dynactin subunit 6 | 1 |
| IPR027247 | Mitochondrial import inner membrane translocase subunit Tim10/Tim12 | 1 |
| IPR016656 | Transcription initiation factor TFIIE, beta subunit | 1 |
| IPR033181 | MICOS subunit Mic26, fungi | 1 |
| IPR027230 | SUMO-conjugating enzyme Ubc9 | 1 |
| IPR017117 | Ribonuclease Nob1, eukaryote | 1 |
| IPR032259 | Enoyl-CoA hydratase/isomerase, HIBYL-CoA-H type | 1 |
| IPR027124 | SWR1-complex protein 5/Craniofacial development protein | 1 |
| IPR002139 | Ribokinase/fructokinase | 1 |
| IPR014306 | Hydroxyisourate hydrolase | 1 |
| IPR004556 | Modification methylase HemK | 1 |
| IPR032567 | LDOC1-related | 1 |
| IPR027120 | Structural maintenance of chromosomes Smc2 | 1 |
| IPR006885 | NADH dehydrogenase ubiquinone Fe-S protein 4, mitochondrial | 1 |
| IPR033196 | Exosome complex component Rrp43 | 1 |
| IPR002759 | Ribonuclease P/MRP protein subunit | 1 |
| IPR019024 | Ribonuclease H2, subunit B | 1 |
| IPR006519 | Ribosomal protein L11, bacterial-type | 1 |
| IPR002639 | Urease accessory protein UreF | 1 |
| IPR032157 | Proteasome assembly chaperone 4 | 1 |
| IPR019398 | Pre-rRNA-processing protein TSR2 | 1 |
| IPR015672 | The Golgi pH regulator/GPCR-type G protein | 1 |
| IPR029922 | Sla1 | 1 |
| IPR015374 | Chs5p-Arf1p binding | 1 |
| IPR019496 | Nuclear fragile X mental retardation-interacting protein 1, conserved domain | 1 |
| IPR013919 | Peroxisome membrane protein, Pex16 | 1 |
| IPR004526 | Glutamyl-tRNA synthetase, archaeal/eukaryotic cytosolic | 1 |
| IPR006993 | SH3-binding, glutamic acid-rich protein | 1 |
| IPR018465 | Centromere protein Scm3/HJURP | 1 |
| IPR003462 | Ornithine cyclodeaminase/mu-crystallin | 1 |
| IPR019152 | Protein of unknown function DUF2046 | 1 |
| IPR028598 | WD repeat BOP1/Erb1 | 1 |
| IPR028971 | NAD-glutamate dehydrogenase | 1 |
| IPR030557 | Vacuolar protein sorting-associated protein 1 | 1 |
| IPR013964 | DASH complex subunit Ask1 | 1 |
| IPR003703 | Acyl-CoA thioesterase | 1 |
| IPR030231 | GPN-loop GTPase 2 | 1 |
| IPR013946 | Nuclear control of ATP synthase 2 | 1 |
| IPR019129 | Folate-sensitive fragile site protein Fra10Ac1 | 1 |
| IPR016341 | Clathrin, heavy chain | 1 |
| IPR027031 | Glycyl-tRNA synthetase/DNA polymerase subunit gamma-2 | 1 |
| IPR006676 | tRNA-splicing endonuclease | 1 |
| IPR011941 | DNA recombination/repair protein Rad51 | 1 |
| IPR018839 | Transcription-silencing protein Clr2 | 1 |
| IPR004450 | Threonine synthase-like | 1 |
| IPR016691 | tRNA guanosine-2'-O-methyltransferase, TRM11 | 1 |
| IPR011082 | Exosome-associated factor Rrp47/DNA strand repair C1D | 1 |
| IPR028879 | NADPH-dependent diflavin oxidoreductase 1 | 1 |
| IPR009446 | Mitochondrial genome maintenance Mgm101 | 1 |
| IPR009163 | ATP adenylyltransferase | 1 |
| IPR008895 | Vps72/YL1 family | 1 |
| IPR028587 | Adenylate kinase 2 | 1 |
| IPR001945 | RAD3/XPD family | 1 |
| IPR008180 | Deoxyuridine triphosphate nucleotidohydrolase/Deoxycytidine triphosphate deaminase | 1 |
| IPR017109 | Adaptor protein complex AP-4, epsilon subunit | 1 |
| IPR007149 | Leo1-like protein | 1 |
| IPR018333 | Squalene cyclase | 1 |
| IPR016527 | Origin recognition complex subunit 4 | 1 |
| IPR004472 | Dethiobiotin synthase BioD | 1 |
| IPR033684 | Protein-lysine N-methyltransferase | 1 |
| IPR001837 | Adenylate cyclase-associated CAP | 1 |
| IPR006257 | Dihydrolipoyllysine-residue acetyltransferase component of pyruvate dehydrogenase complex | 1 |
| IPR022226 | Protein of unknown function DUF3752 | 1 |
| IPR013240 | DNA-directed RNA polymerase I, subunit RPA34.5 | 1 |
| IPR004514 | Glutamine-tRNA synthetase | 1 |
| IPR005716 | Ribosomal protein S5/S7, eukaryotic/archaeal | 1 |
| IPR007224 | Transcription initiation factor Rrn11 | 1 |
| IPR027106 | U4/U6 small nuclear ribonucleoprotein Prp4 | 1 |
| IPR026019 | Ribulose-phosphate 3-epimerase | 1 |
| IPR017106 | Coatomer gamma subunit | 1 |
| IPR014640 | Imidazole glycerol phosphate synthase HisHF | 1 |
| IPR007292 | Nuclear fusion protein, KAR5 | 1 |
| IPR010228 | NADH:ubiquinone oxidoreductase, subunit G | 1 |
| IPR015429 | Cyclin L/T | 1 |
| IPR007356 | tRNA (guanine-N1-)-methyltransferase, eukaryotic | 1 |
| IPR005256 | Anthranilate synthase component I, PabB-like | 1 |
| IPR019171 | Caffeine-induced death protein 2 | 1 |
| IPR030468 | NEDD8-activating enzyme E1 catalytic subunit | 1 |
| IPR016685 | RNA-induced silencing complex, nuclease component Tudor-SN | 1 |
| IPR006225 | Pseudouridine synthase, RluC/RluD | 1 |
| IPR012882 | Protein of unknown function DUF1687, fungi | 1 |
| IPR021276 | Protein of unknown function DUF2855 | 1 |
| IPR012080 | Aspartate-semialdehyde dehydrogenase | 1 |
| IPR004815 | Lon protease, bacterial/eukaryotic-type | 1 |
| IPR021384 | Mediator complex, subunit Med21 | 1 |
| IPR007720 | N-acetylglucosaminyl transferase component | 1 |
| IPR033034 | NADH dehydrogenase [ubiquinone] 1 beta subcomplex subunit 9 | 1 |
| IPR024933 | Septin and tuftelin interacting protein | 1 |
| IPR012890 | GC-rich sequence DNA-binding factor | 1 |
| IPR016219 | Phosphatidylethanolamine N-methyltransferase, fungi | 1 |
| IPR012506 | YhhN-like | 1 |
| IPR030701 | Transcription factor TFIIIB component B'' | 1 |
| IPR029703 | DNA polymerase epsilon catalytic subunit | 1 |
| IPR019560 | Mitochondrial 18kDa protein | 1 |
| IPR027004 | Dolichyl-phosphate-mannose-protein mannosyltransferase 1/5 | 1 |
| IPR004361 | Glyoxalase I | 1 |
| IPR021757 | Ribosomal protein L46 | 1 |
| IPR022233 | TRAPP II complex, TRAPPC10 | 1 |
| IPR019166 | MICOS complex subunit MIC26/MIC27 | 1 |
| IPR026841 | Inositolphosphotransferase Aur1/Ipt1 | 1 |
| IPR005854 | Amidophosphoribosyltransferase | 1 |
| IPR012718 | T-complex protein 1, epsilon subunit | 1 |
| IPR021772 | Protein of unknown function DUF3337 | 1 |
| IPR027525 | Eukaryotic translation initiation factor 3 subunit I | 1 |
| IPR004493 | Leucyl-tRNA synthetase, class Ia, archaeal/eukaryotic cytosolic | 1 |
| IPR028934 | Vacuolar protein sorting protein 26 related | 1 |
| IPR028830 | DNA mismatch repair protein Mlh3 | 1 |
| IPR016558 | DNA primase, large subunit, eukaryotic | 1 |
| IPR026777 | Plasma membrane fusion protein PRM1 | 1 |
| IPR025655 | Peroxisomal membrane protein 14 | 1 |
| IPR009316 | COG complex component, COG2 | 1 |
| IPR012136 | NADP transhydrogenase, beta subunit | 1 |
| IPR019026 | Peptidase M64, IgA | 1 |
| IPR027512 | Eukaryotic translation initiation factor 3 subunit A | 1 |
| IPR027684 | Tubulin-specific chaperone C | 1 |
| IPR009563 | Sjoegren syndrome/scleroderma autoantigen 1 | 1 |
| IPR027243 | Mitochondrial chaperone BCS1/plant AAA ATPases | 1 |
| IPR007303 | TIP41-like protein | 1 |
| IPR004662 | Acetylglutamate kinase | 1 |
| IPR019049 | Nucleoporin protein Ndc1-Nup | 1 |
| IPR006413 | P-type ATPase, subfamily IIA, PMR1-type | 1 |
| IPR024738 | Transcriptional coactivator Hfi1/Transcriptional adapter 1 | 1 |
| IPR033511 | Rho guanine nucleotide exchange factor Cdc24/Scd1 | 1 |
| IPR007064 | NMD3 | 1 |
| IPR024388 | Ribosomal protein L20, mitochondrial | 1 |
| IPR031318 | OPI10 family | 1 |
| IPR004582 | Checkpoint protein Rad17/Rad24 | 1 |
| IPR027921 | Protein of unknown function DUF4598 | 1 |
| IPR031238 | Peroxisomal long-chain fatty acid import protein 1 | 1 |
| IPR016711 | Sds23 | 1 |
| IPR006361 | Uroporphyrinogen decarboxylase HemE | 1 |
| IPR019009 | Signal recognition particle receptor, beta subunit | 1 |
| IPR018628 | Cytochrome c oxidase assembly factor 3, mitochondrial | 1 |
| IPR018559 | Protein of unknown function DUF2015 | 1 |
| IPR016543 | Mitochondria fission 1 protein | 1 |
| IPR019128 | Sister chromatid cohesion protein Dcc1 | 1 |
| IPR002661 | Ribosome recycling factor | 1 |
| IPR016562 | Proteasome assembly chaperone 2, eukaryotic | 1 |
| IPR032053 | Mitochondrial 28S ribosomal protein S34 | 1 |
| IPR021056 | Mitochondrial import inner membrane translocase subunit Tim54 | 1 |
| IPR023426 | Flap structure-specific endonuclease | 1 |
| IPR014006 | Succinate dehydrogenase/fumarate reductase, flavoprotein subunit | 1 |
| IPR006449 | Farnesyl-diphosphate farnesyltransferase | 1 |
| IPR016840 | Glycoside hydrolase, family 43, endo-1, 5-alpha-L-arabinosidase | 1 |
| IPR001154 | DNA topoisomerase II, eukaryotic-type | 1 |
| IPR008901 | Ceramidase | 1 |
| IPR014401 | Ribosomal protein S6, eukaryotic | 1 |
| IPR007727 | Spo12 | 1 |
| IPR028877 | 50S ribosomal protein L18Ae/60S ribosomal protein L20 and L18a | 1 |
| IPR030217 | Nuclear RNA export factor | 1 |
| IPR006331 | Adenosine deaminase-related growth factor | 1 |
| IPR002594 | Glycoside hydrolase family 12 | 1 |
| IPR024338 | Stretch-activated cation channel Mid1 | 1 |
| IPR003691 | Putative fluoride ion transporter CrcB | 1 |
| IPR014807 | Cytochrome oxidase assembly protein 1 | 1 |
| IPR027145 | Periodic tryptophan protein 2 | 1 |
| IPR019310 | rRNA-processing protein Efg1 | 1 |
| IPR013877 | YAP-binding/ALF4/Glomulin | 1 |
| IPR011858 | Phosphoribosylformimino-5-aminoimidazole carboxamide ribotide isomerase, eukaryotic | 1 |
| IPR003780 | COX15/CtaA family | 1 |
| IPR003674 | Oligosaccharyl transferase, STT3 subunit | 1 |
| IPR004638 | Drug resistance transporter EmrB/QacA subfamily | 1 |
| IPR032852 | DNA oxidative demethylase ALKBH2 | 1 |
| IPR017182 | S-adenosyl-L-methionine dependent methyltransferase, Mett10D, predicted | 1 |
| IPR019460 | Autophagy-related protein 11 | 1 |
| IPR004886 | Glucanosyltransferase | 1 |
| IPR016939 | Mitochondrial ribosomal protein S25 | 1 |
| IPR023033 | Alanine-tRNA ligase, eukaryota/bacteria | 1 |
| IPR025187 | Protein of unknown function DUF4112 | 1 |
| IPR025279 | Stress response protein NST1 | 1 |
| IPR019318 | Guanine nucleotide exchange factor, Ric8 | 1 |
| IPR022209 | Pre-mRNA splicing factor | 1 |
| IPR013633 | siRNA-mediated silencing protein NRDE-2 | 1 |
| IPR018626 | Protein of unknown function DUF2347 | 1 |
| IPR027306 | Actin-related protein 2 | 1 |
| IPR012721 | T-complex protein 1, theta subunit | 1 |
| IPR001613 | Flavin amine oxidase | 1 |
| IPR004533 | CDP-diacylglycerol--serine O-phosphatidyltransferase | 1 |
| IPR018864 | Nucleoporin Nup188 | 1 |
| IPR031150 | BBP/SF1 family | 1 |
| IPR021149 | Oligosaccharyl transferase complex, subunit OST3/OST6 | 1 |
| IPR027664 | Actin-related protein 5 | 1 |
| IPR030230 | GPN-loop GTPase 1 | 1 |
| IPR021100 | N-glycosylation protein EOS1 | 1 |
| IPR024888 | U1 small nuclear ribonucleoprotein A/U2 small nuclear ribonucleoprotein B'' | 1 |
| IPR033487 | Transcriptional regulatory protein Dep1 | 1 |
| IPR029704 | UPF0428 family | 1 |
| IPR013471 | Ribonuclease Z/BN | 1 |
| IPR005679 | Ribosomal protein S12, bacterial-type | 1 |
| IPR031606 | Potassium transporter Kch | 1 |
| IPR013963 | DASH complex subunit Dad2 | 1 |
| IPR024864 | Nucleoporin Nup54/Nup57/Nup44 | 1 |
| IPR000741 | Fructose-bisphosphate aldolase, class-I | 1 |
| IPR027110 | Pyruvate dehydrogenase E1 component subunit beta | 1 |
| IPR027170 | Transcriptional activator NFYC/HAP5 subunit | 1 |
| IPR013883 | Transcription factor Iwr1 | 1 |
| IPR022780 | Dynein family light intermediate chain | 1 |
| IPR004554 | Hydroxymethylglutaryl-CoA reductase, eukaryotic/archaeal type | 1 |
| IPR010487 | Neugrin/Rrg9 | 1 |
| IPR027535 | Sulfate adenylyltransferase | 1 |
| IPR027048 | Secretion-regulating guanine nucleotide exchange factor | 1 |
| IPR027235 | Prefoldin subunit 2 | 1 |
| IPR004489 | Succinate dehydrogenase/fumarate reductase iron-sulphur protein | 1 |
| IPR016652 | Ubiquitinyl hydrolase | 1 |
| IPR019321 | Nucleoporin Nup88 | 1 |
| IPR016298 | Histidine biosynthesis trifunctional-protein | 1 |
| IPR004393 | Nicotinate-nucleotide pyrophosphorylase | 1 |
| IPR006941 | Ribonuclease CAF1 | 1 |
| IPR016827 | Transcriptional adaptor 2 | 1 |
| IPR010031 | L-gulonolactone/D-arabinono-1,4-lactone oxidase | 1 |
| IPR025763 | tRNA (guanine-N-7) methyltransferase catalytic subunit Trm8, eukaryote | 1 |
| IPR003370 | Chromate transporter | 1 |
| IPR006509 | Splicing factor, RBM39-like | 1 |
| IPR027059 | Coatomer delta subunit | 1 |
| IPR016266 | DNA polymerase epsilon, subunit B | 1 |
| IPR006238 | Cystathionine beta-lyase, eukaryotic | 1 |
| IPR016494 | 5'-3' exoribonuclease 1 | 1 |
| IPR001465 | Malate synthase | 1 |
| IPR032870 | Alpha-ketoglutarate-dependent dioxygenase alkB homologue 7 | 1 |
| IPR012171 | Fatty acid desaturase | 1 |
| IPR016053 | Haem oxygenase-like | 1 |
| IPR005874 | Eukaryotic translation initiation factor SUI1 | 1 |
| IPR017303 | Mitochondrial import inner membrane translocase subunit Tim44 | 1 |
| IPR027079 | TFIIH subunit Tfb1/p62 | 1 |
| IPR002143 | Ribosomal protein L1 | 1 |
| IPR013863 | Vacuolar import/degradation, Vid27-related | 1 |
| IPR007857 | Protein arginine N-methyltransferase PRMT5 | 1 |
| IPR024662 | Trafficking protein particle complex II-specific subunit 65 | 1 |
| IPR019495 | Exosome complex component CSL4 | 1 |
| IPR021950 | Transcription factor Spt20 | 1 |
| IPR004531 | Phenylalanyl-tRNA synthetase, class IIc, beta subunit, archae/euk cytosolic | 1 |
| IPR010044 | Methylthioadenosine phosphorylase (MTAP) | 1 |
| IPR005782 | P-type ATPase, subfamily IIA, SERCA-type | 1 |
| IPR008591 | GINS complex subunit Sld5 | 1 |
| IPR017862 | SKI-interacting protein, SKIP | 1 |
| IPR005727 | Ribosomal protein L22, bacterial/chloroplast-type | 1 |
| IPR005710 | Ribosomal protein S4/S9, eukaryotic/archaeal | 1 |
| IPR006289 | Transcription elongation factor, TFIIS | 1 |
| IPR003521 | ICln | 1 |
| IPR032672 | TmcA/NAT10/Kre33 | 1 |
| IPR018609 | Bud13 | 1 |
| IPR013862 | Protein of unknown function DUF1753, Golgi | 1 |
| IPR026963 | Aprataxin | 1 |
| IPR025207 | Kinetochore Sim4 complex subunit Fta4 | 1 |
| IPR001631 | DNA topoisomerase I | 1 |
| IPR023273 | RNA (C5-cytosine) methyltransferase, NOP2 | 1 |
| IPR018613 | Protein of unknown function DUF2052, coiled-coil | 1 |
| IPR022896 | Triosephosphate isomerase, bacterial/eukaryotic | 1 |
| IPR024337 | tRNA-splicing endonuclease, subunit Sen54 | 1 |
| IPR007062 | Protein phosphatase inhibitor 2 (IPP-2) | 1 |
| IPR007992 | Succinate dehydrogenase [ubiquinone] cytochrome b small subunit, CybS | 1 |
| IPR024943 | Enhancer of polycomb protein | 1 |
| IPR017149 | Glutathione degradosome, DUG2 | 1 |
| IPR012089 | 2-thiocytidine tRNA biosynthesis protein, TtcA | 1 |
| IPR016267 | UTP--glucose-1-phosphate uridylyltransferase | 1 |
| IPR022771 | Wings apart-like protein | 1 |
| IPR022234 | Protein of unknown function DUF3759 | 1 |
| IPR022968 | Ribosome biogenesis protein | 1 |
| IPR014801 | Mediator complex, subunit Med5, fungi | 1 |
| IPR024747 | Pyridoxamine 5'-phosphate oxidase-related | 1 |
| IPR019329 | NADH:ubiquinone oxidoreductase, ESSS subunit | 1 |
| IPR023610 | Phosphatidylinositol-4-phosphate 5-kinase | 1 |
| IPR028333 | Ribosomal protein S17, archaeal/eukaryotic | 1 |
| IPR027503 | Lon protease homolog, chloroplastic/mitochondrial | 1 |
| IPR001469 | ATPase, F1 complex, delta/epsilon subunit | 1 |
| IPR005711 | Ribosomal protein S5, eukaryotic/archaeal | 1 |
| IPR005339 | GINS complex, subunit Psf1 | 1 |
| IPR003566 | T-cell surface glycoprotein CD5 | 1 |
| IPR031322 | Shikimate kinase/gluconokinase | 1 |
| IPR025795 | tRNA (uracil-5-)-methyltransferase | 1 |
| IPR021641 | Protein of unknown function DUF3245 | 1 |
| IPR024926 | Nucleolar GTP-binding protein 1 | 1 |
| IPR021933 | Protein of unknown function DUF3546 | 1 |
| IPR019002 | Ribosome biogenesis protein Nop16 | 1 |
| IPR018810 | Uncharacterised protein family UPF0662 | 1 |
| IPR018422 | Cation/H+ exchanger, CPA1 family | 1 |
| IPR023251 | Pre-mRNA-splicing factor BRR1 | 1 |
| IPR022185 | Protein of unknown function DUF3712 | 1 |
| IPR027248 | Small nuclear ribonucleoprotein Sm D2 | 1 |
| IPR028945 | WRB/Get1 family | 1 |
| IPR029008 | Rab5-interacting protein family | 1 |
| IPR027027 | GOSR2/Membrin/Bos1 | 1 |
| IPR007967 | Protein of unknown function DUF727 | 1 |
| IPR033599 | RNA polymerase I transcription initiation factor TAF1B/Rrn7 | 1 |
| IPR018574 | Structure-specific endonuclease subunit Slx4 | 1 |
| IPR026847 | Vacuolar protein sorting-associated protein 13 | 1 |
| IPR010971 | Ubiquinone biosynthesis hydroxylase UbiH/COQ6 | 1 |
| IPR026122 | Putative helicase MOV-10 | 1 |
| IPR021181 | Mitochondrial Rho GTPase | 1 |
| IPR005317 | Dipeptidyl-peptidase 3 | 1 |
| IPR006005 | Glutamate synthase, NADH/NADPH, small subunit 1 | 1 |
| IPR022127 | Store-operated calcium entry regulator STIMATE/YPL162C | 1 |
| IPR016492 | Transcription elongation factor, TFIIS-related | 1 |
| IPR007014 | FUN14 | 1 |
| IPR033008 | COP9 signalosome complex subunit 1 | 1 |
| IPR007483 | Hamartin | 1 |
| IPR015688 | Elongation Factor 3 | 1 |
| IPR013246 | SAGA complex, Sgf11 subunit | 1 |
| IPR024109 | Tryptophan-tRNA ligase, bacterial-type | 1 |
| IPR000751 | M-phase inducer phosphatase | 1 |
| IPR026849 | Autophagy-related protein 2 | 1 |
| IPR016712 | Mitochondrial ribosomal protein MRP51, fungi | 1 |
| IPR007724 | Poly(ADP-ribose) glycohydrolase | 1 |
| IPR026832 | Asteroid | 1 |
| IPR005605 | Sporulation/nuclear morphology, Spo7 | 1 |
| IPR033173 | Translational activator Gcn1 | 1 |
| IPR029793 | MBF transcription factor complex subunit Mbp1/Res1/Res2 | 1 |
| IPR019007 | WW domain binding protein 11 | 1 |
| IPR026258 | Signal recognition particle subunit SRP68 | 1 |
| IPR027498 | Ribosomal protein S2, eukaryotic | 1 |
| IPR032955 | Geranylgeranyl transferase type-2 subunit alpha | 1 |
| IPR007128 | Polyamine-modulated factor 1/Kinetochore protein NNF1 | 1 |
| IPR003710 | Ketopantoate reductase ApbA/PanE | 1 |
| IPR014980 | Dopa 4,5-dioxygenase | 1 |
| IPR013251 | DASH complex subunit Spc19 | 1 |
| IPR000806 | Rab GDI protein | 1 |
| IPR027706 | Mitochondrial PGP phosphatase | 1 |
| IPR003694 | NAD(+) synthetase | 1 |
| IPR008688 | ATPase, F0 complex, B chain/subunit B/MI25 | 1 |
| IPR010117 | Para-aminobenzoate synthase | 1 |
| IPR032976 | YjeF N-terminal domain-containing protein, eukaryotes | 1 |
| IPR017075 | mRNA capping enzyme, alpha subunit | 1 |
| IPR005341 | Mitochondrial import inner membrane translocase subunit Tim16 | 1 |
| IPR004789 | Acetolactate synthase, small subunit | 1 |
| IPR024789 | Anaphase-promoting complex subunit 4 | 1 |
| IPR000183 | Ornithine/DAP/Arg decarboxylase | 1 |
| IPR017453 | Glycine cleavage system H-protein, subgroup | 1 |
| IPR001044 | XPG/Rad2 endonuclease, eukaryotes | 1 |
| IPR024169 | Serine-pyruvate aminotransferase/2-aminoethylphosphonate-pyruvate transaminase | 1 |
| IPR021261 | Protein of unknown function DUF2838 | 1 |
| IPR018858 | Protein of unknown function DUF2458 | 1 |
| IPR004562 | Lipoyltransferase/lipoate-protein ligase | 1 |
| IPR020618 | Adenylate kinase isoenzyme 6 | 1 |
| IPR027531 | Eukaryotic translation initiation factor 3 subunit F | 1 |
| IPR005999 | Glycerol kinase | 1 |
| IPR011872 | Homocitrate synthase, fungi/archaea | 1 |
| IPR013837 | ATPase, F0 complex, B chain/subunit B | 1 |
| IPR004698 | Zinc/iron permease, fungal/plant | 1 |
| IPR015525 | Breast cancer type 2 susceptibility protein | 1 |
| IPR021622 | Afadin/alpha-actinin-binding | 1 |
| IPR019012 | RNA cap guanine-N2 methyltransferase | 1 |
| IPR029765 | Diphosphomevalonate decarboxylase | 1 |
| IPR013943 | Mitochondrial protein Pet127 | 1 |
| IPR026895 | ER membrane protein complex subunit 1 | 1 |
| IPR011833 | Glycogen/starch/alpha-glucan phosphorylase | 1 |
| IPR011502 | Nucleoporin Nup85-like | 1 |
| IPR024095 | Vesicle tethering protein p115-like | 1 |
| IPR026827 | Proteasome component Ecm29/Translational activator Gcn1 | 1 |
| IPR004119 | Protein of unknown function DUF227 | 1 |
| IPR027496 | 1,2-dihydroxy-3-keto-5-methylthiopentene dioxygenase, eukaryotes | 1 |
| IPR011387 | Translation initiation factor 2A | 1 |
| IPR033315 | Fanconi-associated nuclease 1-like | 1 |
| IPR033601 | Nuclear receptor 2C2-associated protein | 1 |
| IPR012178 | Replication factor C subunit 1 | 1 |
| IPR019258 | Mediator complex, subunit Med4 | 1 |
| IPR001071 | Cellular retinaldehyde binding/alpha-tocopherol transport | 1 |
| IPR013859 | Protein of unknown function DUF1750, fungi | 1 |
| IPR001816 | Translation elongation factor EFTs/EF1B | 1 |
| IPR028361 | GPI-anchor transamidase | 1 |
| IPR033370 | Conserved oligomeric Golgi complex subunit 1 | 1 |
| IPR030559 | DNA polymerase zeta catalytic subunit | 1 |
| IPR027502 | Inosine triphosphate pyrophosphatase | 1 |
| IPR014430 | Sterol desaturase Scs7 | 1 |
| IPR011877 | Ribokinase | 1 |
| IPR008046 | DNA replication licensing factor Mcm3 | 1 |
| IPR020581 | Glycine cleavage system P protein | 1 |
| IPR005251 | Methylthioribose-1-phosphate isomerase | 1 |
| IPR019384 | Retinoic acid induced 16-like protein | 1 |
| IPR016216 | Monophenol monooxygenase, fungi | 1 |
| IPR021833 | Protein of unknown function DUF3425 | 1 |
| IPR018731 | Autophagy-related protein 13 | 1 |
| IPR027948 | Protein of unknown function (DUF4436) | 1 |
| IPR007714 | Cilia/flagella-associated protein 20/WDR90/C3orf67 | 1 |
| IPR007832 | RNA polymerase Rpc34 | 1 |
| IPR019312 | Protein of unknown function DUF2363 | 1 |
| IPR009287 | Transcription initiation Spt4 | 1 |
| IPR025654 | Peroxisome biogenesis factor 10 | 1 |
| IPR008179 | Phosphoribosyl-ATP pyrophosphohydrolase | 1 |
| IPR022155 | Protein of unknown function DUF3684 | 1 |
| IPR004404 | Dihydroxy-acid dehydratase | 1 |
| IPR016302 | Anthranilate synthase component 2 | 1 |
| IPR030670 | 60S acidic ribosomal protein P0 | 1 |
| IPR013861 | Transmembrane protein DUF1751, eukaryotic | 1 |
| IPR005729 | Ribosomal protein S10, eukaryotic/archaeal | 1 |
| IPR030662 | Diphthine--ammonia ligase/Uncharacterised protein MJ0570 | 1 |
| IPR003162 | Transcription initiation factor TAFII31 | 1 |
| IPR025532 | Glucose-6-phosphate 1-epimerase | 1 |
| IPR003675 | CAAX amino terminal protease | 1 |
| IPR016315 | Protohaem IX farnesyltransferase, mitochondria | 1 |
| IPR016488 | NADH dehydrogenase [ubiquinone] (complex I), alpha subcomplex, subunit 6 | 1 |
| IPR024826 | DNA polymerase delta/II small subunit family | 1 |
| IPR028599 | WD repeat WDR12/Ytm1 | 1 |
| IPR024661 | DNA-directed RNA polymerase III, subunit Rpc31 | 1 |
| IPR005886 | UDP-glucose 4-epimerase GalE | 1 |
| IPR004803 | Queuine tRNA-ribosyltransferase | 1 |
| IPR013893 | Ribonuclease P, Rpp40 | 1 |
| IPR030484 | Serine/threonine-protein kinase Rio2 | 1 |
| IPR004572 | Protoporphyrinogen oxidase | 1 |
| IPR014485 | Predicted phosphoesterase, C1039.02 type | 1 |
| IPR013927 | Transcription factor Opi1 | 1 |
| IPR027667 | Actin-related protein 4 | 1 |
| IPR007587 | SIT4 phosphatase-associated protein family | 1 |
| IPR016477 | Fructosamine/Ketosamine-3-kinase | 1 |
| IPR013218 | Kinetochore-associated protein Dsn1/Mis13 | 1 |
| IPR005982 | Thioredoxin reductase | 1 |
| IPR016680 | NADH dehydrogenase [ubiquinone] (complex I), alpha subcomplex, subunit 8 | 1 |
| IPR014387 | CDP-diacylglycerol-inositol 3-phosphatidyltransferase, eukaryote | 1 |
| IPR032465 | Amino-carboxymuconate-semialdehyde decarboxylase | 1 |
| IPR000231 | Ribosomal protein L30e | 1 |
| IPR016696 | TRAPP I complex, subunit 5 | 1 |
| IPR024982 | Rax2 | 1 |
| IPR013319 | Glycoside hydrolase family 11/12 | 1 |
| IPR023676 | Ribosomal protein S14, type Z, archaeal | 1 |
| IPR033316 | DNA endonuclease RBBP8-like | 1 |
| IPR030481 | Staphylococcal nuclease domain-containing protein 1 | 1 |
| IPR022125 | U3 small nucleolar RNA-associated protein 10 | 1 |
| IPR014732 | Orotidine 5'-phosphate decarboxylase | 1 |
| IPR022878 | V-type ATP synthase catalytic alpha chain | 1 |
