# Supplementary material for: The genome sequence of the commercially cultivated mushroom Agrocybe aegerita reveals a conserved repertoire of fruiting-related genes and a versatile suite of biopolymer-degrading enzymes
Source: BMC Genomics. 2018 Jan 15;19:48. doi: 10.1186/s12864-017-4430-y (PMC5769442; doi:10.1186/s12864-017-4430-y)
Supplement: Supplementary file 6 — Proteins encoded by fruiting-related genes from different agaricomycetes species. (DOCX 34 kb) [file 12864_2017_4430_MOESM6_ESM.docx]

**Table S5** Proteins encoded by fruiting-related genes from different agaricomycetes species^a^

| **Protein** | **Organism** | **Protein-ID** | **Genome** | **Accession number^b^** |
| --- | --- | --- | --- | --- |
| Bri1 | *S. commune* | 2609901 | Schizophyllum commune H4-8 v3.0 | XP_003038897 |
| Bwc2 | *S. commune* | 2608172 | Schizophyllum commune H4-8 v3.0 | XP_003035694 |
| C2H2 | *S. commune* | 1194000 | Schizophyllum commune H4-8 v3.0 | XP_003026630 |
| Exp1 | *C. cinerea* | BAG24407 | Coprinopsis cinerea okayama7#130 | BAG24407 |
| Fst3 | *S. commune* | 2629275 | Schizophyllum commune H4-8 v3.0 | XP_003031320 |
| Fst4 | *S. commune* | 2616096 | Schizophyllum commune H4-8 v3.0 | XP_003034563 |
| Gat1 | *S. commune* | 2622228 | Schizophyllum commune H4-8 v3.0 | XP_003036589 |
| Hom1 | *S. commune* | 2632356 | Schizophyllum commune H4-8 v3.0 | XP_003030056 |
| Hom2 | *S. commune* | 1034289 | Schizophyllum commune H4-8 v2.0 | XP_003029756 |
| Pcc1 | *C. cinerea* | EAU91357 | Coprinopsis cinerea okayama7#130 | EAU91357 |
| Csf1 | *C. cinerea* | AAL73238 | Coprinopsis cinerea AmutBmut pab1-1 v1.0 | AAL73238 |
| Dst1 | *C. cinerea* | EAU89202 | Coprinopsis cinerea okayama7#130 | EAU89202 |
| Dst2 | *C. cinerea* | BAI67190 | Coprinopsis cinerea okayama7#130 | BAI67190 |
| Eln3 | *C. cinerea* | BAC77594 | Coprinopsis cinerea okayama7#130 | BAC77594 |
| Ich1 | *C. cinerea* | BAA32788 | Coprinopsis cinerea okayama7#130 | BAA32788 |
| Aa-Pri1 | *A. aegerita* | AF004297 | - | AF004297 |
| Aa-Pri2 | *A. aegerita* | AAD41222 | - | AAD41222 |
| Aa-Pri3 | *A. aegerita* | AAM00381 | - | AAM00381 |
| Aa-Pri4 | *A. aegerita* | AAP49844 | - | AAP49844 |

^a^Referring to *Agrocybe aegerita* SM51 (=WT-1), *Coprinopsis cinerea* okayama7#130, *C. cinerea* AmutBmut pab1-1 and *Schizophyllum commune* H4-8

^b^Referring to accession numbers within NCBI GenBank
